# Beyond mammals: the evolution of chewing and other forms of oropharyngeal food processing in vertebrates

**DOI:** 10.1002/brv.70129

**Published:** 2026-01-20

**Authors:** Daniel Schwarz, Maja Mielke, Stephan Handschuh, Anthony Herrel, Patrick Lemell, Léa Da Cunha, Nicolai Konow

**Affiliations:** ^1^ Department of Palaeontology State Museum of Natural History Stuttgart (SMNS) Rosenstein 1 Stuttgart 70191 Germany; ^2^ Friedrich Schiller University Jena (FSU), Institute of Zoology and Evolutionary Research Erbertstraße 1 Jena 07743 Germany; ^3^ Laboratory of Functional Morphology, Department of Biology, Faculty of Sciences University of Antwerp Wilrijk 2610 Belgium; ^4^ VetCore Facility for Research/Imaging Unit University of Veterinary Medicine Vienna Veterinaerplatz 1 Vienna 1210 Austria; ^5^ Département Adaptations du Vivant UMR 7179 C.N.R.S/M.N.H.N Bâtiment d'Anatomie Comparée, 55 rue Buffon Paris 75005 France; ^6^ Department of Biology, Evolutionary Morphology of Vertebrates Ghent University Ghent 9000 Belgium; ^7^ Department of Biology University of Antwerp Wilrijk 2610 Belgium; ^8^ Naturhistorisches Museum Bern Bern 3005 Switzerland; ^9^ Department of Evolutionary Biology, Unit for Integrative Zoology University of Vienna Djerassiplatz 1 Vienna 1030 Austria; ^10^ Institut des Sciences de l'Évolution de Montpellier, Université de Montpellier, CNRS, IRD c. c. 64 Montpellier France; ^11^ Department of Biological Sciences University of Massachusetts Lowell 198 Riverside Street Lowell MA 01854 USA

**Keywords:** feeding, oropharyngeal food processing, mastication, chewing, vertebrate, feeding apparatus, morphology, behaviour, form–function

## Abstract

Oropharyngeal food processing exhibits a remarkable diversity among vertebrates, reflecting the evolution of specialised ‘processing centres’ associated with the mandibular, hyoid, and branchial arches. Although studies have detailed various food‐processing strategies and mechanisms across vertebrates, a coherent and comprehensive terminology is lacking. Here, we provide a synthesis, including a unified terminology for the intricate complexity of vertebrate oropharyngeal processing. Among gnathostomes, mandibular food processing predominates, ranging from discrete bites to rhythmic, cyclic chewing facilitated by precise tongue mechanics in aquatic and terrestrial environments alike. By contrast, some taxa have abandoned oropharyngeal processing entirely, relying instead on post‐oesophageal strategies such as gastric milling and chemical digestion. Interestingly, teleost (bony) fishes illustrate the evolutionary trade‐off between increased jaw protrusion for prey capture and reduced mandibular processing capacity. They compensated for this trade‐off by developing derived processing behaviours early in their evolutionary development. Through the re‐evolution of mandibular chewing, they succeeded in utilising all three known processing centres. Mastication is a specialised, dimensionally complex form of unignathic mandibular chewing (i.e. chewing restricted to the lower jaw) exclusive to mammals. However, our findings demonstrate that dimensionally complex forms of mandibular chewing have arisen independently multiple times and are widespread among gnathostomes. Notably, diverse taxa, including elasmobranch stingrays, Australian lungfish, sirenid salamanders, various songbirds, herbivorous turtles, and the tuatara, exhibit complex jaw movements combining arcuate, longitudinal, and sometimes transverse components enabled by specialised jaw joints, suspensions, and intracranial motions (‘cranial kinesis’). From a comparative, functional–morphological perspective, mammalian mastication may best be characterised as dimensionally complex chewing mediated by the secondary or temporomandibular joint. By contrast, analogous dimensionally complex non‐mammalian chewing involving motions confined to the primary or quadrate–articular jaw joint qualifies as pseudomastication. Both mastication and pseudomastication resemble functional masticatory behaviours, while those incorporating intracranial motions and movements of the jaw suspension belong to distinct categories. Our anatomical analysis highlights the convergent evolution of dimensionally complex chewing among gnathostomes and emphasises the importance of comprehensive studies on jaw development and function to deepen our understanding of the evolution of oropharyngeal processing.

## I. INTRODUCTION

Feeding, the fundamental process by which animals, as heterotrophic organisms, acquire food, is pivotal for obtaining the nutrients containing the energy, vitamins, minerals, and trace elements essential for survival. From an evolutionary perspective, feeding plays a critical role as it directly impacts the viability and potential reproductive success of heterotrophs. The digestion of food constitutes a crucial stage within vertebrate feeding. Digestion is the biological process by which food is broken down into smaller, absorbable components, often within the gastrointestinal tract. The resulting nutrients are subsequently absorbed into the bloodstream and utilised by the body of heterotrophs for all life processes.

Vertebrate digestion represents a complex and highly evolved process that reflects their diverse ecological and dietary strategies. Modern vertebrates exhibit a remarkable array of specialised digestive structures. For example, many herbivores, such as ruminants, evolved the means to facilitate the fermentation and breakdown of plant material (e.g. complex, multi‐chambered stomachs). Carnivorous vertebrates, including many reptiles and mammals, often possess powerful jaws and specialised dentition, such as carnassial teeth, for processing meat and bones. Omnivores, like humans, exhibit a combination of these traits, with versatile dentition and a relatively differentiated gastrointestinal tract. In addition, the evolution of accessory digestive organs, such as the liver and pancreas, significantly enhanced the efficiency of nutrient absorption and metabolism in vertebrates.

The vast morphological diversity of vertebrate skulls, tongues, stomachs, and intestines, as well as all the other associated structures of their feeding apparatus, reflects the evolution of various feeding and digestive mechanisms in that lineage. These mechanisms have been the subject of many studies and discussed in several books and reviews. Despite comprehensive and detailed compilations on vertebrate feeding (Schwenk, 2000b; Reilly, McBrayer & White, 2001; Starck & Wang, 2005; Bels & Whishaw, 2019), efforts to integrate and clarify the diverse, historically influenced, and occasionally ambiguous terminology associated with vertebrate feeding and digestion remain scarce and inadequate.

**Fig. 1:**
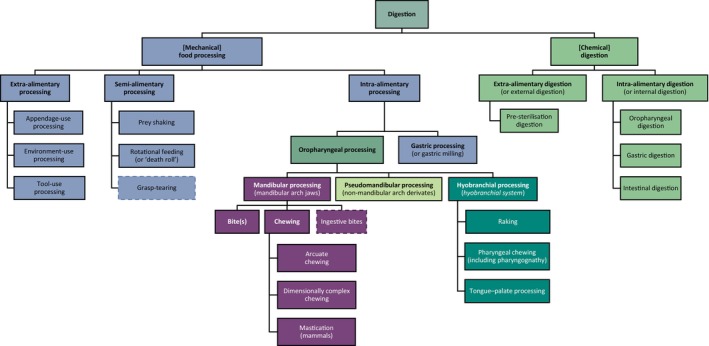
Traditional tree of vertebrate digestive strategies. Vertebrates utilise various mechanisms to digest their food, which is essential for obtaining energy and nutrients vital for life. Digestion describes both (mechanical) food processing and (chemical) digestion. Note that dashed boxes represent mixed behaviours involving ingestion and food processing. This review predominantly examines the form and function of oropharyngeal food processing. Digestive strategies apart from oropharyngeal processing are displayed mainly to enable a more comprehensive understanding and may be incomplete. For detailed definitions of each mechanism, see Table 1.

From a functional perspective, digestion can involve chemical processes, including enzymatic breakdown, and mechanical actions, such as chewing. Hence, ‘digestion’ is often used as an overarching term to refer to both chemical digestion (i.e. chemical processes involved in food reduction) and mechanical food processing (i.e. mechanical breakdown of food) (see Fig. 1 and Table 1). This review primarily focuses on mechanical food processing.

**Table 1:** Description and examples of the distinct traditional types of vertebrate digestion.

Digestion behaviour	Definition	Example(s)
**Digestion**	refers to the breakdown of large, insoluble food compounds through mechanical and/or chemical means, known as comminution or food reduction, into smaller, water‐soluble components for absorption into the blood plasma.	All animals, thus, all vertebrates (at least during certain ontogenetic stages)
**Chemical digestion**	is the breakdown and digestion of food involving chemical processes. While most animals combine mechanical and chemical food processing (a), many species have eliminated mechanical food processing entirely and rely solely on chemical digestion (b).	(a) all animals that do not belong to group b (b) some tadpoles, certain fishes, and most metamorphic frogs
**Extra‐alimentary digestion** (*external digestion*)	**(combining Latin *extra*‐ ‘outside’, and *alimentary* ‘pertaining to the alimentary tract’)** is the process of chemical reduction and digestion of food outside of the alimentary tract. Digestive enzymes are released into the environment surrounding the organism to break down organic material or prey to absorb (some of) the resulting products.	Raptors, such as vultures
Pre‐sterilisation digestion	involves digestive juices being regurgitated onto carcasses, mainly to kill bacteria, making the food safer to eat. The partially digested food is subsequently ingested for further digestion.	Raptors, such as vultures
**Intra‐alimentary digestion** (*internal digestion*)	**(combining Latin *intra*‐ ‘within’ and *alimentary* ‘pertaining to the alimentary tract’)** involves the chemical reduction and breakdown of food in the body.	All animals, thus, all vertebrates (at least during certain ontogenetic stages)
Oropharyngeal digestion	**(connecting *oro*‐ from Latin *ōs* ‘mouth’ and pharyngeal from Late Latin *pharyngeus* for ‘throat’)** refers to the enzymatic breakdown of molecules in the oral or pharyngeal cavities (e.g. amylase initiates the breakdown of carbohydrates into simpler sugars).	Most mammals, many birds, and certain lizards like the green iguana
Gastric digestion	**(gastric, from Greek *gastēr* ‘pertaining to the stomach’)** uses gastric (or stomach) juices to break down molecules in the stomach (e.g. hydrochloric acid and enzymes like pepsin work together to break down proteins into smaller peptides).	Synapomorphy of gnathostomes
Intestinal digestion	**(intestinal, from Latin *intestinum* pertaining to the bowel or gut)** is the enzymatic reduction of molecules in the gut (e.g. enzymes from the pancreas and bile from the liver breakdown carbohydrates, proteins, and fats to be absorbed by the body).	Most mammals and birds, as well as snakes and lizards
**Mechanical food processing**	refers to the physical reduction or preparation of food to aid chemical processing.	Widespread across vertebrates
**Extra‐alimentary processing**	**(combining Latin *extra*‐ ‘outside’, and *alimentary* ‘pertaining to the alimentary tract’)** is the physical processing of food occurring outside of the digestive tract.	Various primates, sea otters, and many birds
Appendage‐use processing	refers to the use of appendages, including hands, claws, and talons, to alter and reduce food mechanically.	Clawed frogs, turtles, raptors (e.g. eagles, hawks, and owls), and various carnivorous mammals like big cats and canids
Environment‐use processing	the environment is used either by combining gravitational acceleration (*via* Earth's gravity field) of deliberately dropped foods or muscular acceleration of held foods with hard environmental surfaces to alter and reduce food mechanically.	Bearded vultures, golden eagles, seagulls, oystercatchers, and California scrub‐jays
Tool‐use processing	involves utilising non‐corporeal resources (items) to alter and reduce food mechanically (e.g. the use of rocks and other hard objects to break or crack open diverse foods).	Chimpanzees, sea otters, and Egyptian vultures
**Semi‐alimentary processing**	**(combining Latin *semi*‐ ‘partially’ and *alimentary* ‘pertaining to the alimentary tract’)** refers to the physical processing of food that takes place both externally and internally, inherently linking these two spheres.	Ancestral vertebrate condition
Prey shaking	involves the predator rapidly shaking or jolting its head or body while holding partially ingested prey with its mouth and teeth. This action generates powerful shear and torsional forces, which can dismember or incapacitate the prey, making it easier for the predator to consume.	Widespread among vertebrates
Rotational feeding (‘*death roll*’)	involves the predator rotating its entire body while holding partially ingested prey with its mouth. This behaviour uses the drag and hydrodynamic resistance of the fluid environment to create a significant momentum difference between predator and prey. Generates powerful shear, torsional, and rotational forces that work together to incapacitate and potentially tear the prey apart.	Hagfish, certain teleost fish, caecilians, salamanders, and crocodilians
Grasp‐tearing	refers to oral grabbing or biting behaviours (i.e. grasping), where food is secured, and force from body or head and neck movements is directly applied to the bitten part. This action results in portions of the food item being ripped away or torn off.	Hagfish, certain eel‐shaped teleosts, snakes, birds of prey, and various tetrapods
**Intra‐alimentary processing**	**(combining Latin *intra*‐ ‘within’ and *alimentary* ‘pertaining to the alimentary tract’)** involves physical processing of the food within the digestive tract.	Widespread among vertebrates
**Gastric processing** (*gastric milling*)	**(gastric, from the Greek *gastēr* ‘pertaining to the stomach’)** is the process of grinding foods in specialised “chewing stomachs” (gizzards).	Birds, many crocodilians, certain actinopterygians, and pangolins use gizzards to process food
**Oropharyngeal processing**	**(connecting *oro*‐ from Latin *ōs* ‘mouth’ and pharyngeal’ from Late Latin *pharyngeus* for the pharynx or throat but also indicating the pharyngeal arches)** is mechanical food processing that occurs in the mouth and throat, usually using pharyngeal arch derivates.	Widespread among vertebrates
**Hyobranchial processing** → hyobranchial system	**(connecting Greek *hyoeides* ‘shaped like the letter upsilon (Υ/υ)’, with Greek *brankhia* ‘gills’, to signify an association with structures related to hyoid and gill arches)** a form of mechanical, oropharyngeal food processing that is powered by derivates of the hyoid and branchial arches (i.e. hyobranchial motions)	Numerous teleosts, certain salamanders, and possibly echidnas and platypuses
Raking	is characterised by physical food processing powered by relatively inflexible ‘tongue‐like’ hyobranchial derivatives (mainly restricted to hyoid arch, or ‘hyoid’) that pull prey backwards across the palatal dentation during the oropharyngeal phase.	Certain teleosts (e.g. osteoglossomorphs)
Pharyngeal chewing (including pharyngognathy)	denotes physical, oropharyngeal food processing driven by uniquely remodelled ‘gill jaws’, typically called pharyngeal jaws – derivatives of the branchial arches – that triturate and manipulate the food.	Certain teleosts (e.g. cyprinids)
Tongue–palate processing	is powered by a relatively flexible and mobile (hyo)lingual system (tongue) that pushes the food against and across the palate (and, if present, its dentition) during the oropharyngeal phase.	Certain salamanders, turtles, and mammals
Pseudomandibular processing	**(pseudo from Greek *pseudes* ‘false’)** is powered by non‐jaw derivates of the mandibular arch in non‐gnathostomes or jaws‐like structures in non‐vertebrates (and may include pseudobites and pseudochewing).	Cyclostomes
**Mandibular processing → jaws**	refers to food processing using mandibular lower and upper jaws (dorsal and ventral mandibular arch derivatives).	Synapomorphy of gnathostomes
Bite(s)	describes one or a few forceful, non‐rhythmic and non‐cyclical contacts between the occlusal surfaces of the jaws (gnathal unit) and the food, which may cause puncturing, crushing, or slicing.	Synapomorphy of gnathostomes
Ingestive bites	refers to a mixture of food ingestion and mandibular processing bites, interspersed with food transport and potentially even swallowing; these behaviours are usually less rhythmic, and cyclical compared to chewing.	Most gnathostomes feeding on foods exceeding their oral cavity or lacking flexible tongues for food transport (e.g. snakes)
**Chewing**	refers to the rhythmic and cyclical mandibular processing using various occlusal surfaces, often resulting in relatively increased food breakdown compared to simple bite(s).	Synapomorphy of gnathostomes
Arcuate chewing	**(from Latin *arcuate* ‘arc‐shaped’, or ‘curved like a bow’)** refers to the vertical, arc‐shaped rotational opening and closing movements of the lower jaw, wherein the jaw moves along a segment of a circle – a path defined by rotation around a relatively fixed jaw joint (*also referred to as arcilinear, arcilineal, orthal, or shearing*).	Certain actinopterygians, most lungfish, and many tetrapods
Dimensionally complex chewing	refers to more than simple arcuate movements of the jaw(s) in which various occlusal surfaces (e.g. beaks or teeth) help break down food.	Certain lungfish, salamanders, many turtles, and the tuatara
Mastication	is the dimensionally complex chewing of mammals that displays pronounced food comminution, combining a flexible tongue that helps position food for efficient breakdown, and a mobile jaw joint that allows for complex movements of the mandible and precise occlusion of their specialised teeth, which together result in a saliva‐mixed bolus.	Certain mammals (absent in myrmecophagous mammals, platypus and certain arcuate chewing hypercarnivores)

Digestion behaviours presented in bold font denote overarching categories, while non‐bold entries listed beneath each signify more specific subcategories or examples that fall within these broader behavioural types.

Food processing is a crucial stage of feeding that is broadly acknowledged for its role in potentially killing prey and in the salivation and comminution of food prior to swallowing across many vertebrates (Hiiemae & Crompton, 1985; Schwenk, 2000a; Schwenk & Rubega, 2005).

Food processing not only helps incapacitate prey but also facilitates and improves subsequent chemical digestion by breaking down the food's structural integrity, piercing its integument, and/or reducing its size, thereby enhancing the efficiency of digestive fluids and increasing the surface area for microorganisms to convert non‐digestible elements like cellulose into usable energy (Lucas *et al*., 2002; Chen, 2009; Witt & Stokes, 2015). Food processing may be categorised based on its location relative to the digestive tract. Extra‐alimentary processing encompasses activities outside the alimentary system, such as using tools, the environment, or appendages. Semi‐alimentary processing involves mechanical manipulation of food both inside and outside the alimentary system, often with environmental support, as seen in prey shaking and rotational feeding. Intra‐alimentary processing happens mainly within the alimentary tract without external assistance and can be subdivided into oropharyngeal and gastric processes, reflecting the structure of the alimentary system.

The principal focus of this review is vertebrate oropharyngeal food processing, which can be divided into hyobranchial, pseudomandibular, and mandibular processing (see Fig. 1 and Table 1). Hyobranchial processing requires specialised pharyngeal jaws derived from the hyoid and often branchial arches, or tongues or ‘tongue‐like’ structures. Pseudomandibular processing can involve the action of non‐mandible derivatives with mandibular arch contributions, exclusive to non‐gnathostome vertebrates like cyclostomes or jaw‐like structures in non‐vertebrates. Mandibular processing functionally includes non‐cyclic bites, ingestive bites, and chewing behaviours. Despite its familiarity, the term ‘chewing’ remains ambiguous. Scientifically, it can be viewed in two major ways: (*i*) as synonymous with ‘mastication’, thus restricted to mammals, and (*ii*) as encompassing any consecutive and rhythmic open–close movements of the jaw aiding intraoral food processing.

## II. CHEWING EQUALS MASTICATION: A HISTORICAL MISCONCEPTION

There seems to be a shared conceptual understanding of the term ‘chewing’, and most non‐specialists would likely attribute it to repetitive (and rhythmic) jaw‐based processing of food – some may also include the presence of teeth if unreflective of many edentulous animals. Conversely, within most dictionaries and occasionally the field of mammalian feeding, the consensus supports the notion that chewing is synonymous with mastication and is regarded as a distinctive trait exclusively observed in mammals (Lubosch, 1907; Hiiemae & Crompton, 1985; Dorland, 2012; McIntosh, 2013). Despite the numerous definitions of mastication that exist today, many scientists might agree on the following: *mastication is the cyclic mechanical breakdown of food into small pieces (pronounced comminution), which are mixed with saliva often to form a fine paste known as the bolus. This process involves the precise occlusion of specialised teeth set in mandibular jaws connected by a mobile jaw joint, allowing for more than simple open–close (arcuate) movements. Additionally, the tongue plays a crucial role in positioning the food to aid its breakdown*. The combination of these characteristics is restricted to mammals, and hence, chewing, when considered synonymous with mammalian mastication, would be intrinsically limited to mammals *via* this definition. However, who was responsible for integrating these mammalian anatomical adaptations into the concept of chewing, especially considering the contrasting collective understanding among non‐specialists?

This question may initially appear irrelevant within the confines of the scientific realm. Nevertheless, given the contemporary inclusion of diverse non‐mammalian taxa such as sauropsids (Throckmorton, 1980; Kraklau, 1991; Herrel & De Vree, 1999, 2009; Herrel, Verstappen & De Vree, 1999a; Ross *et al*., 2010; Mielke & Van Wassenbergh, 2022), lissamphibians (Rull, Solomon & Konow, 2020; Schwarz *et al*., 2020b,c, 2021, 2023; Spence *et al*., 2023; Richard *et al*., 2023), lungfish (Perkins, 1972; Bemis & Lauder, 1986), ray‐finned fish (Gintof *et al*., 2010; Lomax & Brainerd, 2020, 2021), cartilaginous fish (Kolmann, 2016; Kolmann *et al*., 2016; Laurence‐Chasen, Ramsay & Brainerd, 2019; Rutledge, Summers & Kolmann, 2019), as well as cyclostomes (Clark & Summers, 2007; Zintzen *et al*., 2011) into the study of feeding and food processing, it becomes evident that this field has already transcended its historically mammal‐centric terminological framework. Consequently, the inadequate and exclusionary nature of the current terminology within this domain requires reconsideration and revision.

The prevailing interchangeable usage of the terms ‘chewing’ and ‘mastication’ in certain literature may be attributed to several historical factors, including (*i*) the disregard or overlooking of the comparative aspect of the ancient Greek fundamental conceptualisations on mastication, (*ii*) the initial adoption of these terms and their scientific application primarily within the context of human medicine in western countries, thus exhibiting a human‐centric bias (Quincy, 1719; Hunter, 1771), and (*iii*) the initial examination of the functional anatomy and kinematics of chewing primarily focused on humans (Hunter, 1771) and other mammals (Blumenbach, 1795; Lubosch, 1907). As a result, the terms ‘chewing’ and ‘mastication’ are commonly cross‐referenced in dictionaries. Nevertheless, the interchangeable use of these two terms is neither necessary nor helpful in accurately describing the specific behaviour within the scientific realm.

### (1) The origin and initial conception of mastication and chewing

The term ‘mastication’ can be traced back to the Latin *masticare*, which in turn may have its roots in the Greek *mastikhan*, ‘to gnash the teeth’. Therefore, ‘mastication’ may be highlighting the significance of teeth. The earliest recorded scientific discussions on mastication date back to around 350 BCE in Greece. In his *Περὶ ζῴων μορίων* (*On the Parts of Animals*), Aristotle associated it with the prolonged action of (flat) oral teeth or grinding surfaces that may act as teeth, while the presence of a tongue to orient food was considered advantageous but not essential (Ogle, 1911; Barnes, 1995). Two centuries later, Galen introduced the idea that the tongue played a pivotal role in orienting food during mastication in his *Περὶ χρείας μορίων* (*On the usefulness of the parts of the body*) (May, 1968). The conceptual differences suggest that even the ancient Greek academics may have applied divergent interpretations and been engaged in discussions about the term ‘mastication’. Regardless, Aristotle and Galen conceptualised mastication in terms of (flat) oral grinding surfaces, such as teeth or beaks that help with enhanced food reduction. While largely excluding many non‐mammalian species with sharp teeth and lacking grinding surfaces, this definition resulted in a broader application of the term by focusing solely on the fundamental functional prerequisites: using occlusal surfaces for repetitive oral processing in a crushing and grinding manner (including an element of horizontal movement). The term occlusion is often restricted to a dental context, where occlusal surfaces refer to the surfaces of the teeth that come into contact with those of the opposite jaw during biting or chewing. However, we propose using occlusion in a broader, comparative perspective of feeding, referring to the contact of opposing surfaces capable of processing food. Hence, occlusal surfaces can include all forms of mandibular, hyoid, or branchial arch surfaces, such as beak edges, keratinous cusps, pads, rugal folds, and teeth.

Indeed, Aristotle applied the term ‘mastication’ very broadly. He noted that both *Scarus*, a genus of parrotfish with teeth that form a structure resembling a parrot's beak, and spiny crayfish, which possess specialised chewing mouthparts, engage in mastication to process their food. This underscores the point that mastication was initially not exclusive to mammals or limited to the use of true teeth; rather, it involves comprehensive food processing with oral teeth or tooth‐like structures across various species that may lead to enhanced food reduction.

The extensive body of recent literature and the common scientific use of the term ‘mastication’ being restricted to mammalian chewing and the fact that most mammals broadly exhibit chewing behaviours that align with the original definition suggest that we should not revert to its original, broader definition. Therefore, we uphold the convention of confining the term ‘mastication’ to mammalian chewing behaviours.

Conversely, the modern English term ‘chewing’ originates from various Eurasian languages, often implying a primary emphasis on the jaws. Therefore, it may be inferred that ‘chewing’ emphasises the function of the jaws, with no explicit indication of a direct link to (flat grinding) teeth evident in its etymology. There are few references on how the concept of chewing might have been perceived in its early linguistic and biological origins. Nevertheless, given its prevalent use and fundamental etymological meaning, we argue that ‘chewing’ should be broadly defined as *the cyclical and rhythmical biting action of mandibular jaws*. This definition does not include the presence of particular teeth. Consequently, mandibular food processing can involve singular or a few non‐cyclic, non‐rhythmic contacts between the jaws and the food, referred to as bites. Alternatively, it may involve consecutive, rhythmic contacts known as chewing. Both bites and chewing can result in the food being punctured, crushed, sliced, or ground by various occlusal surfaces such as teeth or beaks.

Schwenk (2000a), Reilly, McBrayer & White (2001) and Schwenk & Rubega (2005) were among the first authors to examine vertebrate food‐processing behaviours comparatively and comprehensively and proposed a more functional interpretation of chewing. Their conceptualisations of chewing align closely with the broader definition proposed above. However, the approach of Reilly *et al*. (2001) differed in that they primarily used the main functions of mammalian mastication proposed by Hiiemae & Crompton (1985): first, to reduce material to a condition suitable for swallowing, and second, to facilitate the penetration of digestive enzymes and hence to expedite chemical breakdown. Thus, Reilly *et al*. (2001)'s chewing included every form of mechanical processing, from jaw‐based processing to, e.g. avian gastric milling using gizzards. Conversely, Schwenk & Rubega (2005)'s definition of chewing highlighted the presence of oral teeth that perform repeated, cyclical bites to prepare food for swallowing. This action can result in crushing, puncturing, shearing, and/or grinding, which serve to soften the food and introduce salivary enzymes, but it does not require significant fragmentation or true comminution. As a result, their definition excluded non‐dental processing behaviours, like those observed in turtles and some birds, which they categorised more broadly as oral food processing (Schwenk & Rubega, 2005). Here, we introduce an approach that bridges the gap between these two conceptions, while also allowing for a clear differentiation between ‘chewing’ and ‘mastication’. This approach further allows us to trace chewing behaviours back to their gnathostome origins as proposed by Richard *et al*. (2023).

Both chewing and mastication hinge on mandibular motions. Over time, the definition of mastication has expanded to include a variety of anatomical traits that distinguish it from other forms of oropharyngeal food processing. Traditionally, one of the primary differences has been in the outcome: chewing generally results in relatively little food breakdown, whereas mastication involves a thorough mechanical reduction of food into smaller pieces – known as (pronounced) comminution – which are then mixed with saliva to form a bolus (Schwenk, 2000a; Schwenk & Rubega, 2005). However, defining mastication based solely on these outcomes may not be adequate. For example, non‐mammals that achieve similar degrees of comminution could also be argued to masticate, leading to questions about how to measure ‘enhanced comminution’ and where to establish thresholds for classification. It is also difficult to confirm with certainty if fossil species utilised their capacity for food breakdown to a degree that qualifies as mastication.

Furthermore, not all characteristics traditionally associated with mastication and enhanced comminution serve to differentiate mammalian chewing clearly from that of non‐mammalian species. Instead, the mammalian temporomandibular joint (TMJ), which facilitates their distinctive dimensionally complex chewing, represents the key unique feature of Mammalia and arguably justifies a distinct terminology. This secondary jaw joint replaced the primary quadrate–articular jaw joint (QAJ) seen in non‐mammalian taxa, leading to the development of a ‘new bite’ in mammals (Crompton, 1963). The original QAJ was subsequently repurposed as part of the middle ear, specifically forming the articulation between the auditory ossicles – the malleus and incus – during early mammalian evolution. Alongside the development of the masseter muscle, the TMJ has been suggested to have supported the rise of more sophisticated jaw movements (Schwenk & Rubega, 2005). Consequently, we propose defining mastication as *dimensionally complex chewing mediated by a secondary, temporomandibular joint*. Additional functional or outcome‐related details may be relevant only when discussing or comparing specific masticatory types.

Regardless, both chewing and mastication rely on the motion of the mandibular jaws. Thus, understanding the evolution of mandibular jaws and how they operate may aid in clarifying the presence, absence, and evolutionary trajectory of mandible‐based processing behaviours.

## III. THE RISE OF JAWS AND CHEWING

Different theories exist concerning the evolution of the vertebrate jaw. The two best known are the (neo)classical ventilation theory (Mallatt, 1996, 2008) and the developmental heterotopy theory (Kuratani *et al*., 2001, 2013; Shigetani *et al*., 2002; Shigetani, Sugahara & Kuratani, 2005; Kuratani, 2012, 2021). Despite the ongoing debate on their evolutionary origin, the rise of jaws in gnathostomes was a key innovation in vertebrate evolution, permitting novel feeding behaviours. Oral grasping and biting‐like behaviours likely were present in conodonts and, hence, evolved before the rise of jaws and ‘true’ teeth (Dzik, 1976; Aldridge *et al*., 1995; Purnell, 1995; Aldridge & Purnell, 1996). While teeth preceded the evolution of jaws and initially had a sensory function (Haridy *et al*., 2025), they eventually evolved on opposing processing surfaces such as jaws to support feeding behaviours. The amplification of velocity conferred by the gnathostome lower jaw likely led to the rise of more effective grasping methods, either by jaw prehension (grasping bites) or rapid aquatic suction‐based ingestion (suction feeding), that allowed exploiting more elusive prey (Clark & Summers, 2007). Further, the hyoid‐ and branchial arches could have already supported the suction generation necessary for ingestion and may have served as a bellow for powering intraoral repositioning of the food early during gnathostome evolution.

During aquatic suction‐based ingestion of food (i.e. suction feeding), a powerful opening of the mouth is often combined with oropharyngeal expansion, generating water currents that draw potential food items like smaller animals (i.e. prey) into the mouth (Muller, Osse & Verhagen, 1982; Muller, 1983; Hildebrand *et al*., 1985; Lauder & Shaffer, 1986; Frazzetta, 1994). While divergent suction‐feeding mechanisms were suggested to have been present in some agnathans (Heintz, 1939; Denison, 1961; Janvier, 1995), jaws are commonly interpreted as being essential for the powerful aquatic suction predation observed among most extant fish lineages (Lauder, 1980c; Muller, 1983; Westneat, 2001; Van Wassenbergh *et al*., 2007; Camp & Brainerd, 2015) as well as among many other aquatic gnathostomes (Cundall, Lorenz‐Elwood & Groves, 1987; Deban & Wake, 2000; Lemell *et al*., 2002; Marshall, 2009; Johnston & Berta, 2011; Heiss *et al*., 2013; Marshall & Pyenson, 2019). Thus, it has been suggested that jaws were the basis for the switch from microphagous suspension‐feeding (i.e. slow pumping of highly concentrated suspensions of food‐carrying water into the mouth) to macrophagous particulate feeders (using fast and powerful engulfing of few or singular food objects *via* suction feeding or by using mandibular grasping) (Mallatt, 1984, 1985). While ‘macrophagy’ allowed for the exploration of new food sources, it also posed challenges to the digestive system. Larger food items require proper incapacitation and efficient digestion within a sufficient timeframe to prevent internal damage from struggling prey or waste. Fortunately, the rise of the jaws and with it the increased gape velocity of jawed mouths simultaneously gave rise to novel bite‐based methods of grasping, incapacitation, and processing, including jaw prehension, non‐cyclic processing bites, ingestive bites, and chewing (cyclic bites) which, in turn, may be argued to have facilitated overcoming and consuming these larger, more challenging‐to‐digest prey. Consequently, the shift from micro‐ to macrophagy may have necessitated the development of increased mechanical food‐processing methods, such as chewing, to aid in effective chemical digestion.

Since we define chewing as cyclic intraoral processing using mandibular jaws (cyclic consecutive bites), early gnathostomes were, by definition, the first vertebrates theoretically equipped to chew their food. Indeed, chewing is present in chondrichthyans (Kolmann *et al*., 2016; Rutledge *et al*., 2019), early‐diverging actinopterygians (Sataeva & Kasumyan, 2022), as well as many sarcopterygians (Perkins, 1972; Bemis & Lauder, 1986), including tetrapods (Throckmorton, 1980; Crompton & Parker, 1987; Schwarz *et al*., 2020c). Since chewing appears to be a fundamental gnathostome trait, this review mainly focuses on this group, which contains approximately 99% of all extant vertebrate species.

However, extant cyclostomes (i.e. agnathans or pre‐gnathostome vertebrates) use non‐jaw derivatives of the mandibular arch cyclically to ‘bite’, shake, rasp, and suck up tissue from carrion or live prey (Shelton, 1978; Clark & Summers, 2007; Zintzen *et al*., 2011). Both hagfish and lampreys can cyclically protract and retract their eversible cartilaginous tooth plates, adorned with keratin toothlets, to tear and swallow pieces of food (Clark & Uyeno, 2019). This process can reduce food into tiny pieces or create a slurry paste. Further, the paired medio‐rostral parts of the basal plate work medio‐laterally in hagfish to power the transverse ‘bulging bites’ of their jawless maw (see online Supporting Information, Table S1). Thus, food processing in cyclostomes exhibits characteristics reminiscent of chewing. However, unlike gnathostome chewing, cyclostome oropharyngeal processing differs in two significant ways: it lacks the action of true mandibular jaws, and the primary movement does not feature arcuate shearing. Since the tooth‐bearing region of the cyclostome mouth arises from the mandibular arch (Kuratani, Oisi & Ota, 2016), like jaws in gnathostomes, it is reasonable to categorise cyclostome food processing as *pseudomandibular processing* (including pseudobites and pseudochewing). This terminology emphasises its functional similarity to biting and chewing, while not meeting the criteria for true chewing. This classification is based on the morpho‐functional approach of this review, designed to encompass and address significant evolutionary morphological changes. However, if this review was purely functional, the processing behaviour of cyclostomes could indeed qualify as chewing.

Regardless, while the emergence of jaws in gnathostomes gave rise to true biting and chewing, the origin and diversity of more complex forms of chewing – those involving jaw movements beyond simple open‐close motions, such as longitudinal or transverse movements – remain poorly understood. Chewing relies on the anterior cranial musculature, which might best be referred to as the *jaw musculature*. The term ‘jaw musculature’ is encouraged over the term ‘masticatory muscles’, as mastication is a definitive mammalian characteristic. For complex chewing behaviours to occur, jaw muscles must connect the mandible to various cranial structures in multiple planes, rather than being confined to orientations that are merely perpendicular to the primary axis of jaw movement. Muscles, including those of the jaw, are rarely oriented exclusively perpendicular to the working axis and early gnathostomes may already have been preadapted to perform dimensionally complex jaw movements (Olson, 1961; Mallatt, 1997; Anderson, 2008). The preadapted state for dimensionally complex jaw movements would then have persisted throughout large parts of early vertebrate evolution (Allis Jr, 1922; Lauder, 1980a; Mallatt, 1997; Anderson, 2008). Subsequent shifts in adductor positions and complexity to increase the arcuate jaw closing forces in many puncture crushers (i.e. arcuate chewers with pointed teeth), coincidently amplified the theoretical capability for longitudinal or propalinal jaw movements and, thus, dimensionally complex chewing in tetrapods (Olson, 1961; Reilly *et al*., 2001). However, dimensionally complex chewing only becomes possible when certain articulations of the feeding apparatus allow for multi‐dimensional movements or if the skeletal system can flexibly bend along areas with weakly or unossified bones. This consideration prompts questions about the functional morphology of the vertebrate feeding apparatus, with a particular focus on the skull and its joints.

## IV. FORM AND FUNCTION OF FOOD PROCESSING

**Fig. 2:**
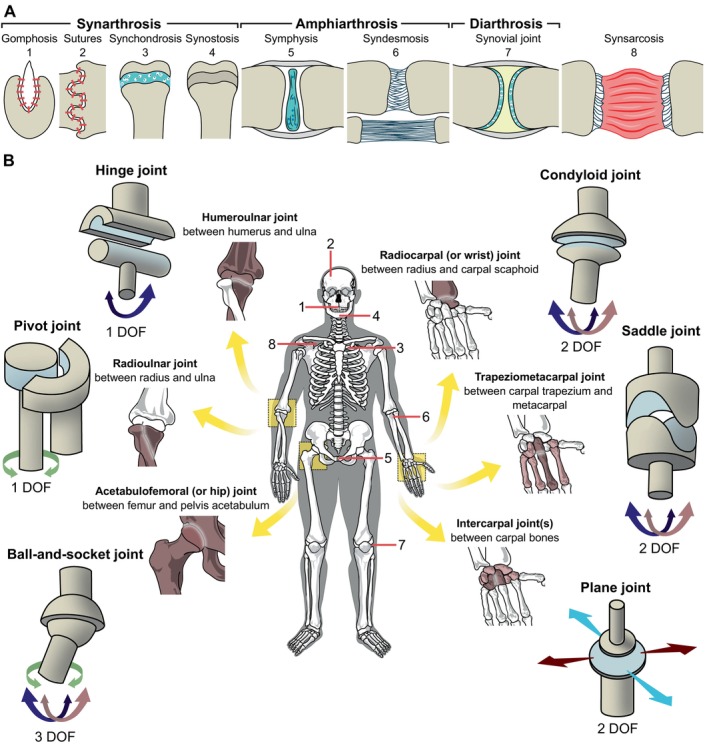
General classification of vertebrate joints. (A) Outline of joint types according to the functional classification. *Synarthroses*: *gomphosis*, fibrous connection between bone and (root of a) tooth; *suture*, fibrous connection between skull bones; *synchondrosis*, hyaline cartilage connecting bones; *synostosis*, fusion of bones. *Amphiarthroses*: *symphysis*, fibrocartilage joins bones which are further connected *via* ligaments; *syndesmosis*, fibrous connective tissue (ligaments) connects bones [tight, upper or loose, lower illustration]. *Diarthrosis*: *synovial joint*, bones with articular cartilage connected *via* fluid‐filled fibrous capsule (i.e. lubricated). *Synsarcosis*, a muscular connection between bones. (B) Conventional synovial joint types, their degrees of freedom and location in the human body. The colouring of the degrees of freedom (DOF) only serves as a simple distinction, the joints do not possess uniform orientation and mechanics. The interpretation of the DOFs depends upon the underlying anatomy and can differ if regarded either from the joints or the organismal perspective. The pivot joint resembles a particular form of the hinge joint with a perpendicular articulation.

The evolution of chewing, particularly in its dimensionally complex forms, required specialised joints within the feeding apparatus. Throughout vertebrate history, a wide array of morphological adaptations enabling intricate jaw motions has emerged. Functionally, joints can be (*i*) largely immobile or fixed (synarthroses), (*ii*) relatively fixed, allowing comparatively slight movements (amphiarthroses), or (*iii*) generally moveable or mobile (diarthroses) (see Fig. 2A). The immobile synarthroses, which include gomphoses, sutures, synchondroses, and synostoses, mainly provide stability and permit only little or no movement. The only slightly moveable amphiarthroses, which include the symphysis and the syndesmosis, often provide both mobility and stability. The mobile diarthroses are used synonymously with lubricated synovial joints and are often argued primarily to facilitate flexibility and movement. However, the general functional classification of joint types encounters exceptions, such as the loose syndesmoses connecting the hemimandibles of certain snakes. While syndesmoses are usually categorised as relatively fixed joints, the syndesmoses in certain snakes can become remarkably loose and flexible, exceeding the motions of any synovial joint (see Fig. 2A, lower illustration in 6). Similar motion potential may only be permitted by the highly moveable and flexible synsarcosis, in which bones are merely linked *via* muscles (e.g. how the mammalian scapula is fixed to the torso or how hyobranchial elements are held in place). However, despite joining two or multiple bones, the synsarcosis is usually not considered to be a joint.

The internal movement potential or mobility of the skull is often attributed to diarthrotic (or synovial) joints, which provide moveable connections between its elements. Synovial joints come in many forms and shapes, including hinge (or ginglymoid), ball‐and‐socket (or spheroid), plane (or arthrodial), condyloid (or ellipsoid), saddle (or selleroid), and pivot (or trochoid) joints (see Fig. 2B for an overview of these joint types and their functionality). However, this basic classification often fails to capture the complexity of biological joints and linkages, which include many functionally diverse types (Radinsky, 1987; Muller, 1996; Wooley, Grimm & Radin, 2005). In fact, various combinations of joint types exist, and these mixed forms can offer more degrees of freedom than traditional joint types, as discussed in Section IV.1.*c*.*ii*.

Regardless, movements of the skull can be highly complex, and contemporary 3D computational approaches are being applied to predict, study, and display their interconnections (Olsen & Westneat, 2016; Olsen, Hernandez & Brainerd, 2020). From an integrative evolutionary–developmental perspective, the skull comprises the cranium, mandible and its suspension, hyobranchial skeleton, and, when present, the hyoid bone and ossicles due to their shared developmental origins, functional interdependence, and modular evolutionary trajectories (Hanken & Hall, 1993a; Hanken & Thorogood, 1993). As all these structures are incorporated into the definition of the skull, it moves as a single unit only when the movement of the entire body alters the position of the head and its skeletal framework. Most skull motions are internal, which means parts of the skull move relative to one other, even during cranial displacements *via* neck motions. This is due to the hyobranchial skeleton typically being connected to both the cranium and the postcranial skeleton. As a result, the hyobranchial skeleton usually changes its orientation relative to the cranium whenever the cranium moves *via* neck motions. Internal motions can occur within or between individual bones or groups of bones in the skull. Some bones or bone groups within the vertebrate skull perform unique functions, which often result in their classification as functionally divergent units. Despite this, a universally accepted terminology for these distinct units is lacking. We opted to divide the skull into functional units to highlight their different roles and tasks, rather than dwelling on their complex evolutionary or developmental origins. This approach reveals the intricate interactions and overlapping functions stemming from the integration and fusion of skull components, potentially offering valuable insights into their adaptability and functionality across various vertebrate groups.

### (1) Functional units of the head and skull

**Fig. 3:**
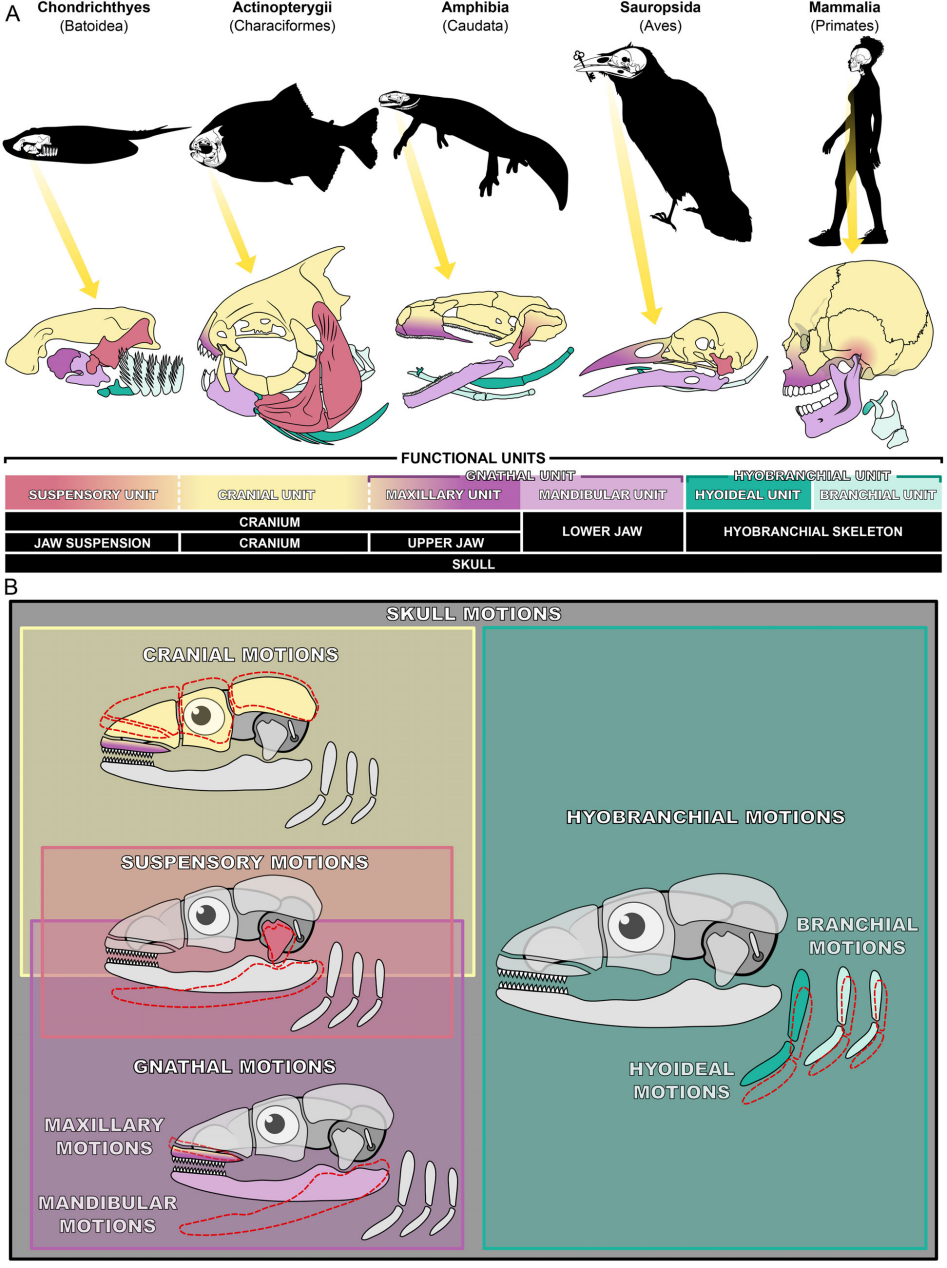
Form and function of skull motions. (A) Functional units of the gnathostome head skeleton (or skull). (B) A novel classification for skull motions based only on the functional units depicted in A. The functional unit motions of the skull can be broadly categorised as internal movements – those occurring between components within a unit – and holistic movements, defined by the unit's relative movement as a whole with respect to other units. These are denoted by the prefixes intra‐ (internal) and uni‐ (holistic), respectively. Skull motions include unicranial‐ and intracranial motions or ‘cranial kinesis’ (i.e. movements between elements of the neurocranium including the facial skeleton). Suspensory motions include unisuspensory‐ and intrasuspensory motions and represent movements along the jaw suspension that carries the jaw(s). Gnathal motions refer to motions of the upper and lower jaw skeletons (maxillary and mandibular motions, respectively) and include jaw‐internal movements (intramandibular motions). Lastly, hyobranchial motions refer to movements of the hyobranchial apparatus or its elements or derivatives relative to one another (intrahyobranchial motions) or to the cranium (unihyobranchial motions).

This section aims to provide an updated vocabulary on the functional units of the head and skull and their motions, consistent with the functional morphology of the vertebrate skull (see Fig. 3). While developing this updated vocabulary, we followed general rules and considerations (see Appendix S1). We combined the widely accepted integrative evolutionary–developmental perspective on the skull (Hanken & Hall, 1993a; Hanken & Thorogood, 1993; Depew & Simpson, 2006) with our morpho‐functional considerations to explore the functions of the head's skeletal elements across their interconnected evolutionary and developmental descent.

The skull bones may be categorised into four functionally distinct main units (see Fig. 3A): (1) *cranial unit*: protecting the brain and the primary sensory organs; (2) *suspensory unit*: connecting the jaw(s) to the cranial unit; (3) *gnathal unit*: mainly involved in feeding and aiding respiration, which contains the (*a*) *maxillary unit*: functional upper jaw, opposing the actions of the lower jaw, and (*b*) *mandibular unit*: functional lower jaw, opposing the actions of the upper jaw; (4) *hyobranchial unit*: mainly facilitating respiration and feeding, containing the (*a*) *hyoideal unit*: aiding food transport and processing, and (*b*) *branchial unit*: supporting the gills and respiration but also food handling.

These functional units relate to anatomically and developmentally defined skeletal structures. The skull includes all units, while the anatomical cranium may comprise the cranial, suspensory, and maxillary units among many extant vertebrates. When fused, these units fulfil divergent roles – protecting the brain and sense organs alongside their eponymous functions – and are illustrated with colour gradients in Fig. 3 to indicate their dual roles. In hyostylic chondrichthyans, many actinopterygians, snakes, and birds, the suspension is independent of the cranium and functions separately, leading to the suspensory unit being represented in Fig. 3 in a single colour (orange). A similar condition may be observed along the maxillary unit (upper jaw). In some hyostylic taxa, such as various chondrichthyans, the maxillary unit might consist solely of the palatoquadrate (epimandibula) which is not fused with the cranial unit. Conversely, in many other groups, the maxillary unit may comprise the maxillary, premaxillary, and palatal bones (e.g. vomer and parasphenoid), with occlusal surfaces opposing the lower jaw. In these groups, these bones attach to and form part of the cranium. Consequently, for hyostylic chondrichthyans, the cranial and maxillary units are shown as distinct – cranial (yellow) and maxillary (purple) – whereas, in most other groups, they are displayed as a continuum with a functional and colour gradient from cranial (yellow) to maxillary (purple). Our new systematic approach identifies four distinct main types of motions as well: *cranial*, *suspensory*, *gnathic*, and *hyobranchial motions* (see Fig. 3B). It is essential to consider that multiple different forms of skull motions may be integrated into one behaviour.

#### (a) Cranial motions

Cranial motions include movements of the entire cranium (i.e. unicranial motions, the Latin prefix *uni*‐ indicating uniform movements) and motions of elements within the cranium to one another (i.e. intracranial motions or ‘cranial kinesis’, the Latin prefix *intra*‐ alluding to internal movements).

##### (i) Unicranial motions

Unicranial motions, or movements of the entire cranium, may be driven actively by muscles anchoring the cranium to the first few cervical vertebrae or passively *via* neck motions arising between cervical vertebrae. These motions encompass flexion (ventral bending), extension (dorsal bending), lateral flexion (side bending), rotation (long‐axis rotation), or combinations thereof. Neck motions may also be attributed to species that do not possess anatomical necks, but potentially exhibit a functional neck, as seen in certain actinopterygians (Camp, 2019, 2021). Although unicranial motions generally exert limited influence on occlusal action, certain non‐hyostylic taxa characterised by elongated skulls and mandibles close to the ground, like crocodiles, evolved pronounced unicranial motions facilitating increased cranial elevation that, together with lower jaw depression, generates gape (Cleuren & De Vree, 1992). Where the maxillary unit is rigidly fused to the cranium, upper jaw movement is effectively integrated with unicranial motions.

##### (ii) Intracranial motions: cranial kinesis

Historically, movements between elements of the cranium (i.e. intracranial motions) were referred to as *cranial kinesis* (from the Greek word: *kinesis*, ‘movement’ or ‘motion’). Versluys (1910, 1912, 1936) provided the first comprehensive theoretical and functional account on the topic of vertebrate cranium‐internal movements. He coined the terms *kinetisch* (kinetic) and *akinetisch* (akinetic) with reference to skulls that permit displacements and movements of different segments of the cranium against each other, regardless of the extent of movement, and those that do not, respectively. Versluys provided the first detailed description of movements in the cranium of fossil and extant tetrapods. The concept was later expanded by including forms of intracranial motions in fishes (Hofer, 1945) and birds (Hofer, 1949). Hofer (1945) further expanded the general terminology of the concept by introducing the terms ‘branchiokinesis’, ‘splanchnokinesis’, and ‘neurokinesis’. Hofer's “neurokinesis” includes intracranial motions (cranial kinesis), while “branchiokinesis” refers to hyobranchial motions and “splanchnokinesis” refers mainly to suspensory motions (i.e. lateral abduction of the palatoquadrate and associated bones against the cranium). Although his endeavour represented an interesting stride towards a comparative applicable system, it did not garner widespread attention.

Intracranial motions are often argued to include, among others, the following sub‐forms. (*i*) *Metakinesis* (Versluys, 1910, 1912), which refers to movements between the ossified chondrocranium and the dermatocranium about a transverse axis that runs through the paroccipital processes of the braincase. (*ii*) *Mesokinesis* (Versluys, 1910, 1912) refers to the dorsal and ventral flexion of the snout around a transverse axis in the skull roof located behind the eye sockets. Mesokinetic movements of the snout are only known from certain squamates (Frazzetta, 1962; Patchell & Shine, 1986; Herrel *et al*., 1999b; Montuelle & Williams, 2015; Handschuh *et al*., 2019) and are always accompanied by some degree of flexion and extension around the transverse axis of the palate [*i.e*. hypokinesis (Metzger, 2002; Jones *et al*., 2011)], which is a prerequisite for permitting dorsoventral flexion and extension of the snout. (*iii*) *Prokinesis* (Hofer, 1949), which refers to a dorsal and ventral flexion of the upper jaw around a transverse axis in front of the eye sockets in birds (Bock, 1964; Van Den Heuvel, 1991; Hoese & Westneat, 1996; Gussekloo, Vosselman & Bout, 2001) and snakes (Cundall & Shardo, 1995; Rieppel & Maisano, 2007). (*iv*) *Rhynchokinesis* (Hofer, 1949), which refers to the flexion of the upper beak of certain birds rostral to the prokinetic hinge (Zusi, 1984; Gussekloo *et al*., 2001; Gussekloo & Bout, 2005; Estrella & Masero, 2007). Two additional motions are (*v*) kinetic movements along the intracranial joint in coelacanths (Dutel *et al*., 2015a) and (*vi*) the various less‐defined and highly complex intracranial movements in teleosts (Westneat, 1990; Westneat & Olsen, 2015; Olsen *et al*., 2020).

#### (b) Suspensory motions

**Fig. 4:**
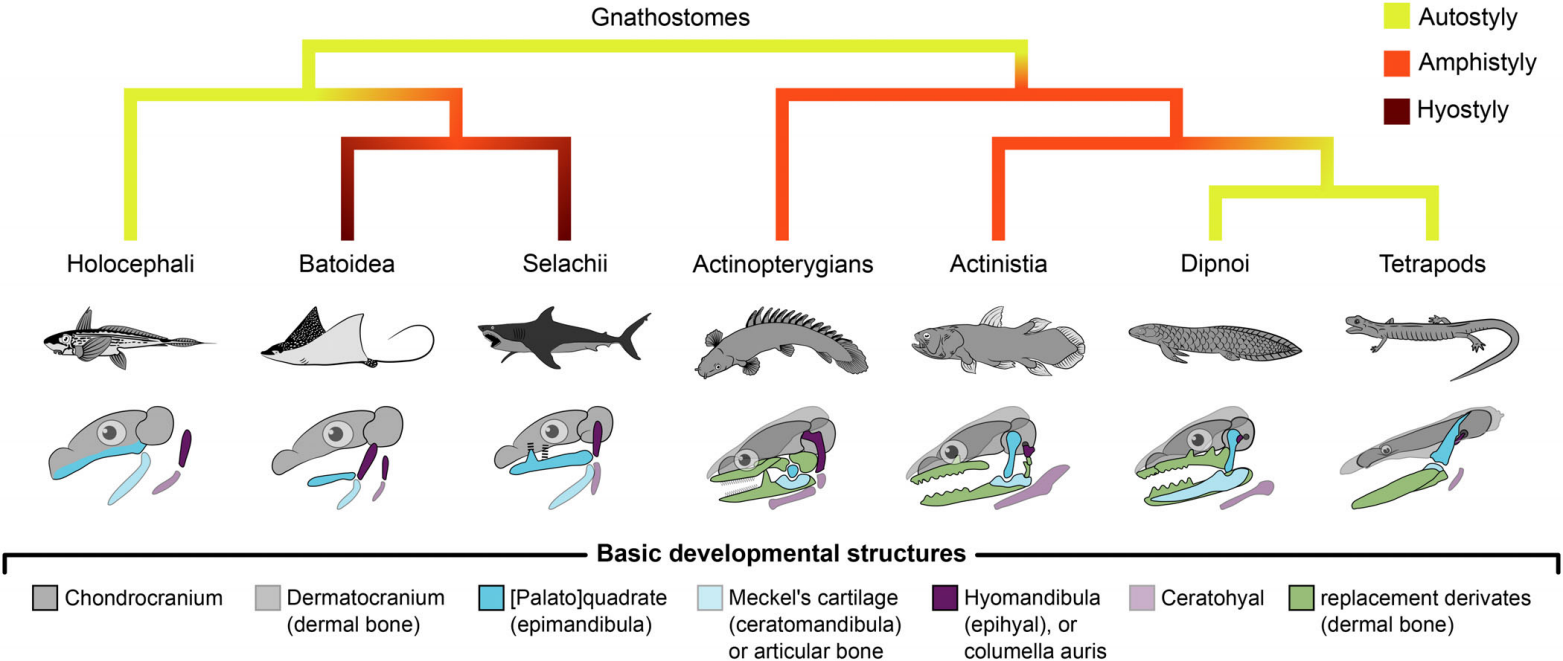
Basic jaw suspension types after Huxley (1876) plotted on a simplified vertebrate phylogeny. Note that, contrary to the assumptions in many historic studies of the evolution of vertebrate jaw suspensions, there is no overarching trend toward any specific form. This is exemplified by the fact that, among Rhipidistia, also known as Dipnotetrapodomorpha, the ancetrally amphistylic condition has been replaced by autostyly.

Jaw suspensions, the cranium–jaw interfaces, are the structures that mount or merge the mandibular jaws and the cranium. Precisely 150 years ago, Huxley (1876, p. 40) studied “[…] the manner in which the mandibular arch is connected with the skull” and defined the three primary forms of how jaws can be suspended from the cranium (see Fig. 4): (*i*) *autostyly*, in which the first pharyngeal arch, the mandibular arch (i.e. the jaws), articulates directly with the neurocranium; (*ii*) *hyostyly*, in which the dorsal element of the second pharyngeal or hyoid arch (i.e. the epihyal or hyomandibula), becomes the primary suspension for the jaws, which may be loosely connected to the cranium *via* ligaments; and (*iii*) *amphistyly*, which resembles a combination of these two (Huxley, 1876).

All vertebrate suspension forms can be classified into these three broad categories. Nevertheless, the diversity of morphologically different conditions cannot be phylogenetically or anatomically captured solely through this basic distinction. Hence, this fundamental terminology remains insufficient and imperfect. An urge to expand and complete the picture has persisted since Huxley's work (Pollard, 1895; Gregory, 1904; De Beer, 1937; Hofer, 1945; Meinel, 1968; Starck, 1979; Kardong, 1997; Wilga, 2002). This led to the creation of various updated terms designed to describe novel forms of jaw suspension. However, even if these terms were correctly to capture the different conditions of vertebrate jaw suspension, the sheer amount of new terminology necessary to account for the diverse vertebrate conditions that evolved (~ 30), meant that their broad, general application was predestined to fail. Consequently, we revert herein to the simpler, albeit outdated, classification of auto‐, hyo‐, and amphistyly, which adequately meets the needs of this review. Moreover, our findings reveal that jaw suspensions often are not useful when evaluating jaw function, as identical suspension forms can result in differently functioning jaw systems. This discrepancy arises because the defined jaw suspension forms are primarily rooted in topology, focusing on the spatial arrangement and connections between anatomical structures rather than their functional morphology. Therefore, as previously suggested (Wilga, 1997; Motta & Wilga, 2001), our considerations indicate that jaw suspensions do not serve as reliable indicators of jaw movement potential.

Regardless, hyostylic as well as amphistylic jaw suspensions often feature relatively free and moveable jaws in relation to the (neuro‐)cranium. In certain species, these jaw suspensions appear to promote suspensory motions, which in turn may facilitate unique, dimensionally complex chewing mechanisms. Suspensory motions encompass all movements of the jaw suspension (or ‘suspensorium’) that are inherently connected with the mandible and result in adjustments to the position of the jaw joint and, consequently, the individual mandibular rami or the entire mandible. The amalgamation of the cranial, suspensory, and maxillary elements in various auto‐ and amphistylic animals cause a functional connection among these skull components. Due to this integration, suspensory motions may trigger corresponding intracranial‐ and/ or maxillary movements. Such induced motions are characteristic of certain actinopterygians, caecilians, lizards, snakes, and birds and contrast with independent motions. Suspensory motions potentially add additional complexity to the action of the occlusal surfaces and can be categorised into two main types (unisuspensory and intrasuspensory) that might function independently or in combination with intracranial movements.

##### (i) Unisuspensory motions

During unisuspensory motions, the jaw suspension, as a single functional element, moves the jaw. Certain chondrichthyans, including sharks and rays, as well as Acipenseriformes (sturgeons and paddlefishes), exhibit unisuspensory jaw movements facilitated by a stick‐like mobile hyomandibula. This anatomical arrangement, characteristic of these taxa, is known as *hyostyly*. Although jaw suspension types are intended to classify anatomical (topological) configurations, there is a common assumption that hyostyly also encapsulates the functional movement potential of these suspensions. Consequently, ‘hyostyly’ may be applied ambiguously to refer to both the general form and the functional capacity of this type of jaw suspension in certain taxa.

In a similar way, certain tetrapods display *streptostyly*, which involves movements of the quadrate resembling a simple, unbranched, stick‐like jaw suspension about the cranial region, typically the braincase (Stannius, 1856). This arrangement enables the jaw(s) to move forwards, backwards, and possibly laterally, with these motions conveyed to the jaw through a chain‐like articulation between bones – hence the term ‘strepto‐’, derived from the Greek *streptós* (*στρεπτός*), meaning ‘chain‐like’. As with the term ‘hyostyly’, ‘streptostyly’ has been used ambiguously, referring to both the form and function of such jaw suspensions (Stannius, 1856; Versluys, 1912). While many forms of intracranial motions involve induced or coupled streptostylic suspension movements, there are also independent instances of such streptostylic motions (Throckmorton, 1976, 1978; Herrel & De Vree, 1999).

*Pleurokinesis* refers to unisuspensory motions where the jaw suspension is formed by several bones of the cheek (including the quadrate) that move as a single functional unit (Pfannenstiel, 1932; Iordansky, 1989, 2000). During jaw opening, this unit is abducted, while during jaw closing, it is adducted, resulting in lateral movement of the jaw joint relative to the braincase, leading to lateral (wish‐boning) movements of the mandibular rami. Because the functional unit involved in pleurokinesis includes several bones that are typically considered as part of the cranium, pleurokinesis represents a combination of suspensory motions and intracranial motions (‘cranial kinesis’).

##### (ii) Intrasuspensory motions

Intrasuspensory motions are present across many actinopterygians possessing complex jaw suspensions containing multiple movably connected skeletal elements that are often oriented in a row (i.e. chain‐like), potentially providing relatively complex movements compared to unisuspensory motions. Due to the numerous bones involved, actinopterygian jaw suspensions exhibit significant variation. As a result, attempts to introduce a comparative terminology that accounts for these divergent forms have largely failed, likely due to the overwhelming number of terms (Hofer, 1945; Meinel, 1968). Although such complex, detailed anatomical terminology may prove useful for categorising behaviours by their general location in the skull, the emergence of computational modelling for studying skull motions in detail (Olsen & Westneat, 2016; Olsen *et al*., 2020) enables any skull joint to be explored within a network of linkages *in silico*. This development may be argued to have resolved the need for an extensive and complex terminological system of intrasuspensory motions.

#### (c) Gnathal motions

Gnathal motions comprise all movements of the primary functional jaws, the mandible or lower jaw (*mandibular motions*) and the functional upper jaw (*maxillary motions*), which carry the opposing occlusal surfaces and are directly involved in, and critical for, various gnathostome behaviours, including feeding. Gnathal motions are usually the primary mechanism driving mandibular processing and may involve movements of both the maxillary and mandibular unit in hyostylic species.

##### (i) Maxillary motions

**Fig. 5:**
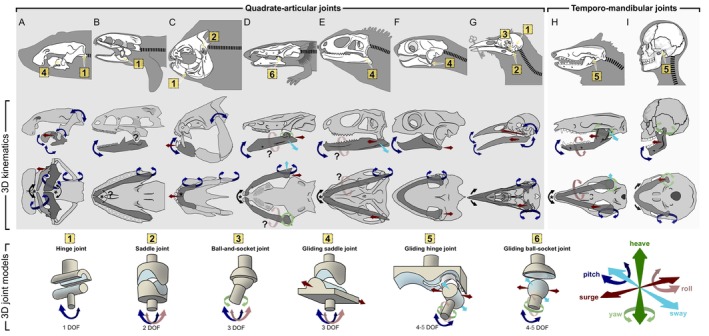
Functional morphology of vertebrate jaw mobility. The top row displays the outline and skull of the considered taxa: (A) chondrichthyans, batoids, *Fluvitrygon signifer* (UF:Fish:166816, MorphoSource ID (ms‐id): 000443225); (B) non‐teleost actinopterygians, bichirs, *Polypterus senegalus* (UMMZ:FISH:195008, ms‐id: 000483259); (C) teleosts, pacu, *Piaractus brachypomus* (ANSP:FISH:166685, ms‐id: 000027533); (D) lissamphibians, salamander, *Siren intermedia nettingi* (SMNS 15426, ms‐id: 000364316); (E) reptiles, rhynchocephalians, *Sphenodon punctatus* (UF:Herp:14110, ms‐id: 000011065); (F) reptiles, testudines, *Cuora mouhotii* (Blüml, 2019); (G) Aves, corvids, *Corvus albus* (l‐jz:JZ:55, ms‐id: 000167314); (H) mammals, opossum, *Didelphis sp*. (du:ea:204, ms‐id: 000029046); and (I) mammals, hominids, *Homo sapiens* (nlm:NLM:2, ms‐id: 000359364). The second and third rows present, respectively, the lateral and ventral perspectives of the functional morphology for the taxa under consideration. The mobility of vertebrate jaws arises from a variety of structural configurations, including distinct jaw joints, flexible interramal joints, forms of intracranial motions, or mobility of the jaw suspension. Detailed descriptions of the degrees of freedom associated with these structures and their relation to chewing terminology are provided in the bottom row, Fig. S1, and Table S2. Note that some joints are depicted in a disarticulated state to enhance structural comprehensibility. Additionally, arrows indicating the direction of operation and degrees of freedom may vary in orientation to reflect the different three‐dimensional orientations of the jaw joint models. In mammals, the temporomandibular joint (TMJ) can accommodate up to five degrees of freedom (DOF) when a flexible mandibular symphysis is present. In species with fused hemimandibles, the TMJ allows for four DOF. The gliding ball‐and‐socket joint found in sirenid salamanders is theoretically capable of providing equivalent degrees of freedom. Nevertheless, biplane kinematic analyses have thus far confirmed only four degrees of freedom: pitch, yaw, sway, and surge. An asterisk denotes the presence of a flexible interramal joint, and when coupled with a black arcuate side‐to‐side arrow, it indicates the presence of mandibular ‘wish‐boning’. A question mark indicates that a movement, which may be specified with semi‐translucent arrows, is considered possible, but lacks experimental confirmation at present.

Maxillary motions describe the movements of the maxillary unit relative to the mandibular unit. Maxillary motions can function independently or be triggered by intracranial movements. In hyostylic taxa, such as many sharks, rays, and sturgeons (see Figs 3, 4, and 5A), maxillary motions are independent because the upper jaw is not fixed to the cranium, allowing it to move freely. By contrast, most auto‐ and amphistylic species typically lack maxillary motions, except for species displaying certain forms of intracranial or unicranial motions. In such auto‐ and amphistylic species, the motion of the functional upper jaw (maxillary unit), which largely derives from dermal cranial bones in osteichthyans, may be part of the intracranial motion capacity, as illustrated by the overlap of cranial and gnathal motions in Fig. 3B. Comparative studies on such maxillary motions among gnathostomes displaying ‘cranial kinesis’ remain insufficient to support definitive sub‐category classifications. However, given the complexity of the maxillary unit among gnathostomes, it is plausible that the range of possible motions would surpass a *modus vivendi* in terminological classification, as seen with intrasuspensory motions. Alternatively, motions of the entire cranium, may power part of the gnathal motions as seen in crocodiles (see Section IV.1.*a*.*i*).

##### (ii) Mandibular motions

Mandibular motions describe the movements of the mandibular unit relative to the maxillary unit. To understand better the potential motions of the mandibular unit, a detailed consideration of its functional morphology is essential.

###### (ii.1) The ‘jaw joint’

Despite multiple joints being related to the vertebrate jaws, the term ‘jaw joint’ is widely used to refer to the joints that connect the maxillary and mandibular unit or connect the mandibular unit to the cranium. The jaw joint in gnathostomes was ancestrally synovial (diarthrotic) (Haines, 1942; Bemis, 1986; Askary *et al*., 2016; DeLaurier, 2019; Sharma, Haridy & Shubin, 2025) and illustrates, together with the jaw, definitive gnathostome characteristics. The gnathostome jaw represents a functional unit comprising bilaterally articulated hemimandibles, which may be connected flexibly *via* ligaments or fibrocartilage or rigidly fused at their anterior meeting point. Within this anatomical framework, none of the conventional synovial joint types (see Fig. 2B), including the ball‐and‐socket joint known for its considerable (three) degrees of freedom (DOF), could theoretically accommodate the dimensional complexity observed during mammalian mastication.

However, the mammalian jaw joint exhibits up to five DOF during mastication: *pitch* (arcuate open–close movement), *surge* (longitudinal fore–aft translations, often referred to as propalinal or proal) (Hiiemae & Crompton, 1985; Hylander, 1985; Hiiemae, 2000; Herring, 2007), *sway* (transverse, left and right translation) (Stilson *et al*., 2023), *yaw* (transverse, rotational left and right excursions) (Weijs, 1994; Hiiemae, 2000; Grossnickle, 2017), and *roll* (hemimandibular long‐axis rotation) (Kay & Hiiemae, 1974; Oron & Crompton, 1985; Zazhigin & Voyta, 2019; Bhullar *et al*., 2019; Stilson *et al*., 2023). For more information, see Fig. S1 and Table S2.

Indeed, the structure of the mammalian TMJ does not conform to a simple mechanical model. Instead, the TMJ often resembles a ‘ginglymoarthrodial joint’, which combines features of both a hinge (or ginglymoid) joint and a plane (or arthrodial) joint (see Fig. 5H and I). Notably, mouth opening in mammals is not solely a result of arcuate mandibular rotation. Instead, it entails a two‐stage behaviour whereby rotation along the hinge‐like area (pitch) initiates the first phase of mouth opening. The subsequent degrees of opening involve a combination of rotation and sliding within the TMJ (Zhang, Gersdorff & Frahm, 2011) (see, for example, https://youtu.be/4LabpJ50kIM, or animated https://youtu.be/WU8zq2iwRS4?si). Without delving excessively into the intricacies of mammalian mastication in this context, it becomes apparent that synovial joints, particularly those of the jaw, frequently necessitate complexity beyond conventional classifications.

Interestingly, jaw joints develop their particular form based on genetics and biomechanical strains occurring during usage, which regulate cell orientation (Brunt *et al*., 2015; Miyashita *et al*., 2020). Since the jaw joint seems to form partially due to how it is used during the early developmental stages, a very early start of oropharyngeal food processing might actively shape the future functionality of the gnathostome jaw joint during ontogeny – just as it can change the feeding behaviour and kinematics (Montuelle & Williams, 2024). However, it could be argued that such plastic developmental changes in morphology are more likely to occur in species that begin feeding while the skull and large parts of the feeding apparatus are still cartilaginous, as is the case with many amphibians or certain fishes. Irrespective of the developmental intricacies of jaw joints, it is clear that they play a crucial role in enabling dimensionally complex jaw movements and our findings indicate that multiple vertebrate lineages have independently evolved jaw joint configurations that enable such complex movements (see Fig. 5A,D,E,F,H,I).

###### (ii.2) Intramandibular joints

Aside from the main axes of mandibular motions around the jaw joint, there can also be *intramandibular motions*. We recommend using ‘intramandibular motions’ as an overarching term that encompasses both intra‐ and interramal motions, which may also be referred to as intra‐ and interhemimandibular motions for added clarity.

*Intraramal joints* are articulations within the imandible, that enable intraramal motions (Lubosch, 1916, syn. streptognathy or streptognathism) which typically involve movements between the distal, tooth‐bearing dentary bone and the more proximal articular bone or other ‘postdentary’ bones, specifically within each lateral arm or ramus of the mandible (also known as the hemimandibles). Intraramal joints and motions are evolutionarily widespread and found in teleosts (Konow *et al*., 2008; Gibb *et al*., 2015), certain birds (Lubosch, 1929; Bühler, 1970; Yanega & Rubega, 2004; Meyers & Myers, 2005; Dawson *et al*., 2011), rorqual whales (Field *et al*., 2010), and various squamates (Callison, 1967; Kley & Brainerd, 1999; Rieppel & Zaher, 2000; Kley, 2001). Where they are found, intraramal joints have been demonstrated to be functionally important and often permit novel and diverse feeding behaviours (Konow & Bellwood, 2005; Konow *et al*., 2008; Ferry‐Graham & Konow, 2010; Gibb *et al*., 2015; Martinez, Tovar & Wainwright, 2022), However, given the lack of detailed 3D functional analyses across these groups, detailed considerations of these joints are omitted here. The general structure of intraramal joints suggests that they can often be functionally regarded as an additional set of hinge‐like jaw joints that increase the range of motion and potentially the DOF in the mandibular system. Hence, in terms of movement potential, they typically extend the gape or mouth opening angle but can also function as a mechanism for gape closing (Konow *et al*., 2008). Nonetheless, there is still a lack of comprehensive functional studies on the role of intraramal joints in intraoral food processing.

*Interramal joints* refer to the articulations connecting both hemimandibles, or mandibular rami, at the anterior midline that may enable interramal motions. Distinctive interramal motions are present across batoids (Kolmann, 2016; Laurence‐Chasen *et al*., 2019), salamanders (Cundall *et al*., 1987; Matsumoto, Fujiwara & Evans, 2024), potentially *Sphenodon* (Jones *et al*., 2012), snakes (Kardong, 1977; Cundall & Greene, 2000; Moon *et al*., 2019), and certain mammals (Lieberman & Crompton, 2000; Bhullar *et al*., 2019). The widespread occurrence of interramal flexion raises the question of how such motions are facilitated by the anatomical structure of these joints.

Interramal joints are commonly referred to as ‘mandibular symphyses’ regardless of the kind of connection between the bones. However, in conventional anatomy, a symphysis as a type of amphiarthrosis is understood as a fibrocartilaginous connection between two bones (see Fig. 2A, part 5). This type seems to be present between the hemimandibles of certain, if not most, gnathostomes. However, there are three major types of interramal joints: a fused interramal joint (‘mandibular synostosis’) rigidly connecting both hemimandibles (Lieberman & Crompton, 2000), the common fibrocartilage connection (‘mandibular symphysis’), usually enabling relatively limited movements (Bhullar *et al*., 2019; Stilson *et al*., 2023), and the rare condition in which only loose interosseous membranes (fibrous connective tissue) link both mandibular rami (‘mandibular syndesmosis’), enabling distinctive movement potential (Lee, Bell & Caldwell, 1999). Therefore, we suggest using the term ‘mandibular symphysis’ only when referring to the fibrocartilaginous connection between the individual hemimandibles.

Interramal joints of most gnathostomes take the form of mandibular symphyses, providing flexibility and stability for a variety of diets and feeding habits (Lieberman & Crompton, 2000; Stilson *et al*., 2023; Matsumoto *et al*., 2024). Certain gnathostomes developed even greater hemimandibular mobility relying on syndesmotic interramal joints (Lee *et al*., 1999; Laurence‐Chasen *et al*., 2019). Relatively flexible interramal joints, both symphysis and syndesmosis, enable complex and/or asymmetrical hemimandibular movements in batoids (Laurence‐Chasen *et al*., 2019), giant salamanders (Cundall *et al*., 1987; Matsumoto *et al*., 2024), mammals (Stilson *et al*., 2023), and potentially *Sphenodon* (Jones *et al*., 2012). However, the relationship between mobility and the general type of the interramal joint is not universal (Scott, Hogue & Ravosa, 2012). Crocodiles serve as notable examples of this disconnect, as they evolved bony crests and grooves that interlock and reinforce the cartilaginous interramal joint, providing a functional alternative to complete fusion of the hemimandibles (Holliday & Nesbitt, 2013; Lessner *et al*., 2019). This suggests that certain symphyses can also be relatively stiff and immobile and that the detailed anatomy must be considered.

Regardless, divergent feeding mechanics and behaviours appear to benefit from the different hemimandibular connections that provide diverging movement potentials (Lee *et al*., 1999; Lieberman & Crompton, 2000; Hogue & Ravosa, 2001; Laurence‐Chasen *et al*., 2019; Lessner *et al*., 2019; Bhullar *et al*., 2020). In summary, both intra‐ and interramal joints significantly impact the motion potential of the lower jaw and, hence, must be considered when investigating the mechanics of chewing and other mandibular motions.

#### (d) Hyobranchial motions

Hyobranchial motions are introduced as a parental category to refer to movements of the hyobranchial unit, relative to the cranium and usually also to its parts to one another. The hyobranchial unit arises from the pharyngeal arches and includes the bones of the hyoid arch, sometimes referred to as ‘hyoid’, and the bones of the branchial arches, which usually form the branchial basket that carries the gills in most fish and salamanders of a larval aquatic morphotype. The hyobranchial unit, characterised by its complexity and interconnectedness, rarely allows independent movement of its parts due to intricate and flexible interactions. This complexity results in hyobranchial motions that combine internal and holistic movements (i.e. intra‐ and unihyobranchial motions). However, the potential range of motions across all element combinations in the hyobranchial unit surpasses a terminological framework, much like with intrasuspensory complexity seen across actinopterygians. Despite this, these motions may still be grossly categorised into hyoideal and branchial types to pinpoint their origin and main characteristics.

##### (i) Hyoideal motions

Hyoideal motions involve the movements of the hyoideal unit, composed of bony hyoid arch elements relative to one another and the cranium. These structures often aid in feeding processes like suction feeding, processing, and hydrodynamic transport in aquatic species. Combinations of mandibular‐ and hyoideal motions might be referred to as *hyomandibular motions*. One example of hyomandibular motions results from a functional link between the mandible and ceratohyal (*via* the hyomandibular ligament) that is present in many aquatic vertebrates and enables transmitting powerful hyoid movements onto the jaw to aid suction feeding (Lauder, 1985; Lauder & Shaffer, 1985; Reilly & Lauder, 1990; Olivier *et al*., 2014; Dutel *et al*., 2015b; Ramsay & Wilga, 2017).

##### (ii) Branchial motions

Branchial motions are movements of and within the branchial unit, composed of bones of the branchial arches, and are fundamental to the respiratory and feeding mechanisms of many aquatic species. In various fish and larval‐stage salamanders, these movements enable efficient gill ventilation. Additionally, branchial motions are integral to food‐handling strategies like suction feeding, hydrodynamic transport, and pharyngeal processing, demonstrating their vital role in aquatic species.

The evolutionary innovations that developed to fulfil the primary roles of the original hyobranchial system from aquatic gnathostomes in both amphibious and, eventually, terrestrial tetrapodomorphs are especially intriguing. The skeletal elements supporting the tongue in tetrapods may be seen as homologous to (parts of) the hyobranchial unit, given their shared function, development, and positional relationship. In species with tongues, certain hyoid arch elements along with branchial arch contributions usually form the tongue skeleton. As a result, it may be argued that aspects of the hyobranchial unit's motility or motion capacity have evolved to be integrated into lingual (skeletal) motions in taxa possessing ‘true tongues’. Lingual motions frequently facilitate feeding processes such as suction feeding, hydrodynamic transport, tongue prehension, lingual transport, and tongue–palate processing, while also aiding in respiration and occasionally contributing to sound production (vocalisation).

The primary sound‐producing organ, the larynx, is traditionally regarded as part of the neck or throat but maintains functional and developmental connections to the hyobranchial system. Hence, aspects of the hyobranchial unit's motility have evolved to become *laryngeal motions* in taxa displaying laryngeal structures or a true larynx (Russell & Bauer, 2021; Gutjahr *et al*., 2024). By contrast, the avian syrinx originates independently from the hyobranchial unit (Kingsley *et al*., 2018) and does not fit within the framework of either hyobranchial or laryngeal motion.

### (2) Evolutionary novelty: auditory motions

Given our functional approach to compare the units of the skull, the novel purpose of certain derived structures may be argued to necessitate individual assessment. The primary jaw suspension, the hyomandibula or epihyal, evolved into the first auditory bone known as the columella in tetrapods (or stapes in taxa with more than one ossicle). Subsequently, the bones forming the primary jaw joint evolved into the incus, derived from the palatoquadrate or epimandibula, and the malleus, originating from Meckel's cartilage or ceratomandibula, collectively forming the three mammalian auditory ossicles. The auditory ossicle(s) represent functionally novel structures while being topologically and developmentally part of the skull, contributing to its motion capacity.

*Auditory motions* involve the movement of the auditory bone(s) or ossicles during sound conduction. In pre‐mammalian tetrapods, auditory motions are restricted to movements of the columella auris (which became the stapes in mammals) about the cranium. By contrast, auditory motions in mammals describe the highly derived movements of mammalian auditory ossicles (malleus, incus, and stapes) with respect to one another and the cranium. In extant species, food processing is not directly linked to auditory motions, although the opposite may sometimes be true. The evolutionary connections between the columella in stem tetrapods or the auditory ossicles in mammals, along with the jaw and its suspension in their ancestral taxa, make the feeding and auditory capabilities of these groups evolutionarily intriguing. This is especially the case because developing effective sound sensing and feeding mechanisms was critical amid substantial jaw restructuring. At present, considerable research is focused on understanding the evolutionary changes that have shaped the auditory and feeding mechanisms in mammals (Navarro‐Díaz, Esteve‐Altava & Rasskin‐Gutman, 2019; Schultz, 2020; Tseng *et al*., 2023; Meng & Mao, 2024). However, the detailed evolution of the columella and how it changed feeding and sound sensing capabilities during stem and early tetrapod evolution remains underexplored and seems to warrant further study.

## V. OROPHARYNGEAL FOOD PROCESSING: A SYSTEMATIC APPROACH

**Fig. 6:**
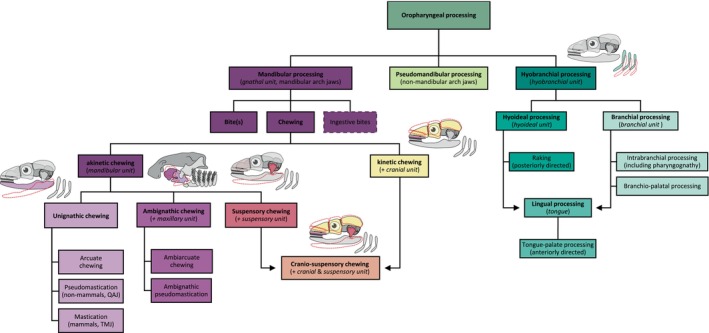
Revised tree of vertebrate oropharyngeal processing strategies. Please note that ingestive bites represent a mixed behaviour involving ingestion and food processing. For detailed definitions, see Table 2. The classification of kinetic chewing and cranio‐suspensory chewing remain largely unresolved, mainly because investigations into the connection between intracranial motions and chewing remain limited. Please also note that all mandibular processing behaviours can (*a*) be functionally ambignathic when substantial unicranial motions – such as cranial elevation and depression – contribute to gape formation and occlusion, or (*b*) encompass intramandibular motions, including intraramal and interramal movements, that expand their range of motion. Intraramal motions along the intraramal (or intrahemimandibular) joints typically increase gape potential, whereas interramal motions occurring along relatively flexible hemimandibular connections – such as symphyses or syndesmoses – permit more complex hemimandibular dynamics. The occurrence of these unicranial or intramandibular motions can be indicated by appending ‘functionally ambignathic’, ‘intraramal’, or ‘interramal’ to the overarching processing strategy, thereby providing greater specificity. Hemimandibles fused by synostosis exhibit considerable rigidity but may afford enhanced stability compared with the more common interramal joint type, the symphysis. QAJ, quadrate–articular joint; TMJ, temporomandibular joint.

This section seeks to integrate the relatively theoretical concepts of Section IV into an updated systematic review of oropharyngeal food‐processing behaviours (see Fig. 6 and Table 2). *Hyobranchial processing* may be broadly classified into hyoideal and branchial processing, delineated by which components of the hyobranchial arch drive the (or most of the) behaviour. Bony fishes exemplify this distinction, utilising not only mandibular jaws but also specialised elements of the hyoid arch and complex branchial arch jaws (pharyngeal jaws) to handle food within the oropharyngeal cavity. *Hyoideal processing* manifests as raking, or ‘tongue‐biting’, where specialised hyoid arch elements serve as bony, toothed “tongues” to scrape food posteriorly across the dentate palate (Konow & Sanford, 2008a).

**Table 2:** Description and examples of distinct types of oropharyngeal processing strategies.

Oropharyngeal processing strategy	Definition	Example(s)
**Oropharyngeal processing**	**(connecting *oro*‐ from Latin *ōs* ‘mouth’ and pharyngeal from Late Latin *pharyngeus* ‘pharynx’ or ‘throat’ but also indicating the pharyngeal arches)** is mechanical food processing in the mouth and throat, usually using pharyngeal arch derivates.	Most vertebrates
**Hyobranchial processing** → hyobranchial unit	**(connecting Greek *hyoeides*, meaning shaped like the letter upsilon (*Υ*/*υ*), with Greek *brankhia* ‘gills’, to signify an association with structures related to hyoid and gill arches)** a form of mechanical, oropharyngeal food processing that is powered by derivates of the hyoid‐ and branchial arches (i.e. hyobranchial motions)	Numerous teleosts, certain salamanders, and possibly echidnas and platypuses
**Hyoideal processing** → hyoideal unit	**(connecting Greek *hyoideus*, meaning shaped like the letter upsilon (*Υ*/*υ*), with the English suffix ‐eal to signify its association with the former)** refers to physical food processing during the oropharyngeal phase facilitated by hyobranchial structures or hyoid arch derivatives.	Certain teleosts
Raking	is characterised by physical food processing powered by relatively inflexible and immobile tongue‐like’ hyobranchial derivatives (mainlyrestricted to the hyoid arch) that pull prey backwards across the palatal dentition during the oropharyngeal phase.	Certain teleosts (e.g. osteoglossomorphs)
**Branchial processing** → branchial unit	**(branchial from Greek *brankhia* ‘gills’, thus pertaining to gills)** involves physical food processing during the oropharyngeal phase powered by peculiarly remodelled ‘gill jaws’, commonly referred to as pharyngeal jaws (branchial arch derivatives).	Certain teleosts
Intrabranchial processing (including *pharyngognathy*)	**(combining Latin *intra*‐ ‘within’ and Greek *brankhia* ‘gills’)** refers to oropharyngeal food processing powered by branchial jaws that triturate the food against one another. Pharyngognathy is a derived form of intrabranchial processing in which the lower pharyngeal jaw consists of the fused fifth ceratobranchials.	Certain teleosts (e.g. *Oreochromis niloticus* and *Astatotilapia burtoni*)
Branchio‐palatal processing	**(combining Greek *brankhia* ‘gills’ and palate ‘roof of the mouth’)** refers to branchial derivates triturating food primarily against the roof of the mouth and pharynx (i.e. the palate). The term ‘pharyngeal mastication’ is occasionally used but is misleading and should be avoided, as mastication is restricted to mammals.	Certain teleosts (e.g. *Cyprinus carpio* and *Ctenopharyngodon idella*)
**Lingual processing** → tongue	**(lingual from Latin *lingua* ‘tongue’, thus pertaining to tongue)** involves physical food processing *via* the tongue, which is a distinctive, highly modified (largely) muscular structure derived from the hyobranchial apparatus, notable for its flexibility and mobility.	Certain salamanders, turtles and possibly echidnas and platypuses
Tongue–palate processing	is powered by a relatively flexible and mobile (hyo)lingual system (or tongue) that pushes the food (anteriorly) against and across the palate (and its dentition) during the oropharyngeal phase.	Certain salamanders, turtles, and mammals
**Pseudomandibular processing** → non‐mandibular arch jaws	**(pseudo from Greek *pseudes* ‘false’)** is powered by non‐jaw derivates of the mandibular arch in non‐gnathostomes or jaws‐like structures in non‐vertebrates (and may include pseudobites and pseudochewing).	Cyclostomes
**Mandibular processing** → gnathal unit, mandibular arch jaws	refers to food processing using the gnathal unit, comprising the lower‐ and the upper jaw. Specifically ‘mandibular’ relates to the mandibular arch jaws (dorsal and ventral mandibular arch derivatives), and thus the gnathal unit. In certain cases, particularly among relatively longirostrine species with snouts close to the ground, unicranial movements may act in concert with lower‐jaw motion to produce gape and occlusion. Consequently, mandibular processing in these instances may additionally be described as ‘functionally ambignathic’ (see also “Ambignathic chewing”), thereby blurring the distinction between ambignathic and non‐ambignathic mechanisms.	Synapomorphy of gnathostomes
Bite(s)	describe one or a few forceful, non‐rhythmic and non‐cyclical contacts between the occlusal surfaces of the jaws (gnathal unit) and potential food objects, which may cause puncturing, crushing, or slicing. Bites may display the functional and morphological diversity of chewing (e.g. ambignathic bites).	Synapomorphy of gnathostomes
Ingestive bites	refers to a mixture of food ingestion and mandibular processing bites, potentially interspersed with food transport and swallowing; these behaviours are usually less rhythmic and cyclical compared to chewing. Ingestive bites may display the functional and morphological diversity of chewing (e.g. ambignathic bites).	Most gnathostomes feeding on foods exceeding their oral cavity or lacking tongues for food transport (e.g. snakes)
**Chewing**	refers to the rhythmic and cyclical processing using mandibular arch‐derived ‘true’ jaws and various occlusal surfaces, often resulting in increased food breakdown compared to simple bite(s).	Synapomorphy of gnathostomes
**Akinetic chewing** → mandibular unit	**(combining a from Greek *an*‐ ‘not’ or ‘without’ and kinetic from Greek *kinēsis* ‘movement’ or ‘motion’, to signify the absence of intracranial motions or ‘cranial kinesis’)** is a form of chewing that lacks kinetic intracranial motions.	Lungfish and many tetrapods
**Unignathic chewing**	**(connecting Latin *uni*‐ ‘one’ and Greek *gnathos* ‘jaw’)** is identified by occlusal action usually being (almost) entirely driven by lower jaw motions. Regardless, pronounced unicranial motions may still render certain unignathic chewing behaviours functionally ambignathic.	Certain actinopterygians, all lungfish, and many tetrapods
Arcuate chewing	**(from Latin *arcuate* ‘bow‐like’)** refers to bow‐shaped, vertical rotational opening–closing movements of the lower jaw (*also referred to as arcilinear, arcilineal, orthal, or shearing*).	Certain actinopterygians, most lungfish, and many tetrapods
Pseudomastication	**(connecting Greek *pseudo‐* ‘false’ or ‘fake’ and mastication for mammalian chewing)** includes dimensionally complex chewing *via* a primary jaw joint, the quadrate‐articular joint (QAJ). Various occlusal surfaces potentially aid food breakdown in taxa that use pseudomastication.	Certain lungfish, salamanders, many turtles, and the tuatara
Mastication	denotes dimensionally complex chewing enabled by a secondary jaw joint, the temporomandibular joint (TMJ). While flat molariform teeth facilitating food breakdown or pronounced comminution are occasionally regarded as essential, pseudomastication and mastication may be functionally indistinguishable, rendering the presence of the TMJ the primary discrete differentiator.	Certain mammals (absent in myrmecophagous mammals, platypus and certain arcuate chewing hypercarnivores)
**Ambignathic chewing** → + maxillary unit	**(connecting Latin *ambi*‐ ‘both sides’ and Greek *gnathos* ‘jaw’)** describes akinetic chewing involving occlusal interactions generated by seperate movements of both the mandibular unit (lower jaw) and the maxillary unit (upper jaw). Thus, true ambignathic chewing is characterised by independent maxillary movements and hence restricted to hyostylic and certain amphistylic species. This distinguishes it from functionally ambignathic behaviours (see also ‘Mandibular processing').	Sharks, rays, crocodiles and certain actinopterygians
Ambiarcuate chewing	**(connecting Latin *ambi*‐ ‘both sides’ and *arcuate* ‘bow‐like’)** is a form of ambignathic chewing that is restricted to arcuate movements of the upper and lower jaws.	Certain sharks and batoids (compressive chewing cycles)
Ambignathic pseudomastication	is a form of ambignathic chewing that includes dimensionally complex chewing movements of the upper and lower jaws.	Batoids (shearing overbite)
**Suspensory chewing** → + suspensory unit	involves additional motions between the jaw suspension and neurocranium. Consequently, these mechanisms produce more complex lower jaw movements and occlusal actions, frequently encompassing longitudinal (antero‐posterior) and lateral (side‐to‐side) motions. These occur either (a) directly *via* a singular, often rod‐like suspension system (unisuspensory motions like ‘hyostyly’ or ‘streptostyly’), or (b) complex motions between a chain‐like suspension (intrasuspensory motions) may be induced by or coupled with intricate intracranial motions known as ‘cranial kinesis’ [see cranio‐suspensory chewing].	(a) certain chondrichthyans and lizards (e.g. *Uromastyx aegyptia*), and (b) various actinopterygians and certain lizards
**Kinetic chewing** → + cranial unit	**(kinetic from Greek kinēsis ‘movement’ or ‘motion’, indicating intracranial motions or ‘cranial kinesis’)** denotes dimensionally complex chewing arising from the integration of intracranial movements in kinetic skulls through moveable joints within the cranial unit, affecting the upper jaws motions (maxillary motions). These shifts alter the orientation of the jaws' occlusal surfaces and are accompanied by either (a) arcuate or (b) dimensionally complex lower jaw movements, producing kinetic chewing or pseudomastication, respectively.	Remains theoretical, as in studied species, intracranial motions are usually linked to suspensory movements (see cranio‐suspensory chewing).
**Cranio‐suspensory chewing** → + cranial & suspensory unit	describes a form of chewing that integrates dimensionally complex intracranial and suspensory movements. It occurs in auto‐ and amphistylic taxa with moveable joints in the cranial unit, including the maxillary unit or upper jaw. This configuration permits intracranial upper jaw movements combined with suspensory lower jaw motions, producing complex occlusal actions.	Certain birds and lizards as well as potentially numerous actinopterygians

The functional–morphological basis, the respective functional unit of the skull (see Section IV.1), that enables the processing behaviour is indicated with an arrow (→). Behaviours presented in bold font denote overarching categories, while the non‐bold entries of the same background colour listed beneath, signify more specific subcategories or examples that fall within these broader behavioural types.

*Branchial processing*, meanwhile, is exemplified by the ‘pharyngeal chewing’ or better pharyngeal processing of bony fishes, involving both ancestral pharyngeal processing and the derived condition known as pharyngognathy (or pharyngognathic processing). Pharyngognathy refers to a suite of morphological specialisations of the pharyngeal jaws – secondary jaw structures in fishes – characteristic of groups such as cichlids and wrasses (Liem, 1973; Wainwright & Longo, 2017). Hallmarks of pharyngognathy include: (*i*) the fusion or close suturing of paired fifth ceratobranchials – the lower pharyngeal jaws – into a single, plate‐like element; (*ii*) suspension of this fused plate by a muscular sling attached to the neurocranium; and (*iii*) the development of robust joints connecting the dorsal surfaces of the upper pharyngeal jaws to a raised protuberance beneath the neurocranium (Kaufman & Liem, 1982; Wainwright *et al*., 2012; Wainwright & Longo, 2017).

Although a more functionally grounded and parsimonious approach has been proposed, utilising the term ‘pharyngognath’ to encompass all teleosts employing pharyngeal jaws to handle and transport food (Vandewalle, Parmentier & Chardon, 2000), the existing widespread understanding of pharyngognathy complicates efforts to redefine it synonymously. Hence, a broader, more inclusive terminology may be necessary. Current distinctions between ancestral pharyngeal processing and pharyngognathy are rooted in anatomical details rather than fundamentally distinct functions. For instance, the fusion of lower pharyngeal jaw bones parallels fusion of the mandibular symphysis, and does not necessarily result in a different general function of chewing by itself. Nevertheless, two fundamentally distinct modes of pharyngeal jaw function exist – *intrabranchial processing*, where pharyngeal jaws operate antagonistically against one another, and *branchio‐palatal processing*, where they work against specialised palatal structures (Vandewalle *et al*., 2000; Wainwright *et al*., 2012; Gidmark, Tarrant & Brainerd, 2014). We suggest pharyngognathy may be considered a special case within the broader category of intrabranchial processing. Branchio‐palatal processing, on the other hand, is noteworthy because the lower pharyngeal jaws do not oppose upper pharyngeal jaws, but rather a novel cranial structure termed the basioccipital pad. The unique evolutionary history, structure, and modified motility of this ‘functional upper pharyngeal jaw’, justifies its distinct classification within pharyngeal processing.

‘True’ tongues, an evolutionary novelty likely associated with the water‐to‐land transition in early tetrapodomorphs, constitutes a remarkable hyobranchial structure. The skeletal elements supporting the tetrapod tongue are homologous with components of the hyobranchial unit. Accordingly, *lingual processing* behaviours such as tongue–palate processing (Heiss, Schwarz & Konow, 2019) form a distinct subset of hyobranchial processing.

Lingual skeletal motions are often functionally linked to chewing, facilitating the precise placement and orientation of food within the oropharyngeal cavity during processing. *Chewing* and *biting per se* only require movements of the mandibular arch, generally through mandibular motions, yet may incorporate independent maxillary movements in hyostylic taxa or induced maxillary movements in auto‐ and amphistylic taxa exhibiting suspensory and/or intracranial motions. Dimensionally complex mandible movements arise either from specialised jaw joints granting multiple DOF (see Section IV.1.*c*.*ii*) or through suspensory motions that introduce longitudinal and transverse components to the baseline arcuate movement (see Section IV.1.*b*). Furthermore, motions occurring at intra‐ and interramal joints can impart additional functional versatility to the jaw apparatus, modifying occlusal interactions. Intracranial motions, often referred to as ‘cranial kinesis’, markedly influence biting and chewing mechanics by altering the spatial position and orientation of the functional upper jaw relative to the lower jaw during processing. These changes affect the occlusal interface between the maxillary and mandibular units, enabling occlusal actions of greater complexity than simple arcuate chewing. Consequently, certain intracranial, suspensory, and mandibular motions – including movements within or between hemimandibles – facilitate jaw dynamics exceeding simple arcuate motions, culminating in dimensionally complex occlusion.

Chewing may be categorised along two distinct frameworks, each offering unique evolutionary insights. The first distinguishes between simple, single‐plane (one‐dimensional) chewing – commonly termed arcuate chewing – and more complex behaviours involving multi‐planar motions, often described as dimensionally complex. This framework has recently gained popularity (Laurence‐Chasen *et al*., 2019; Schwarz *et al*., 2021; Mielke & Van Wassenbergh, 2022). Nevertheless, upon examining the full (known) spectrum of chewing behaviours, this approach appears less advantageous than an alternative strategy focused on the morpho‐functional capacity of gnathostome skulls (see Fig. 6 and Table 2). This new strategy helps understanding the anatomical basis of movements and enables more precise differentiation between functionally similar behaviours that clearly differ in their evolutionary history. Employing this approach, chewing may be broadly categorised into two primary types: *akinetic* and *kinetic chewing*.

Akinetic chewing can be further categorised into *unignathic*, *ambignathic*, and *suspensory chewing*. Unignathic chewing refers to chewing that is (almost) exclusively driven by motions along the mandibular unit or lower jaw and includes three subcategories: (*i*) *Arcuate chewing* is the mechanically and kinematically simplest form, characterised exclusively by vertical rotational opening–closing or ‘elevation–depression’ movements of the lower jaw (also known as arcilinear, arcilineal, orthal, or shearing movements). (*ii*) *Mastication* is restricted to mammals and can be defined as a dimensionally complex form of mandible‐based chewing along the TMJ that results in occlusal surface interactions exceeding simple arcuate motions. The novel definition of mastication presented here remains restricted to mammals but offers a more parsimonious framework for differentiating mammalian chewing from that of non‐mammalian species. (*iii*) *Pseudomastication* shares the same functional definition as mammalian mastication but is observed in non‐mammal species that possess a primary jaw joint (i.e. QAJ). Considering their overlapping functional properties, both mastication and pseudomastication are best regarded as part of the overarching category of functional masticatory behaviours.

Ambignathic chewing refers to chewing in which both lower and upper jaw movements power occlusal action. Ambignathic jaw movements are characteristic of many hyostylic taxa, including rays, sharks, and sturgeons, in which the upper jaw is loosely connected or detached from the neurocranium, allowing it considerable freedom of movement. Ambignathic chewing encompasses *ambiarcuate chewing*, defined by arcuate or arcilinear motions of both the upper and lower jaws, as well as *ambignathic pseudomastication*, which entails upper and lower jaw movements along the primary jaw joint that surpass mere arcuate opening and closing.

It is important to note that in most auto‐ and amphistylic species, the primary movement is generated by the lower jaw, with the upper jaw remaining largely stationary (particularly in tetrapods) or only minimally affecting gape. However, certain species demonstrate functionally ambignathic behaviours, wherein direct (uni)cranial and cervical motions actively move the cranium – and thus the upper jaw – exerting a significant influence on gape. This mode of *functional ambignathic chewing* is well illustrated in crocodiles (Cleuren & De Vree, 1992). By contrast, taxa possessing hyostylic suspensions with ‘true’ ambignathic jaw motions usually largely lack or display less pronounced functional ambignathic chewing.

Suspensory chewing describes chewing arising from the combined action of mandibular movements at the jaw joint and motions of the jaw suspension at its cranial articulation. This dual articulation generates occlusal patterns more complex than those of simple arcuate motions.

Kinetic chewing, unlike its akinetic counterpart, incorporates additional intracranial motions within the cranial unit that houses the functional upper jaw in certain autostylic and amphistylic taxa, resulting in occlusal dynamics that are more intricate than simple arcuate movements. In extant gnathostomes, intracranial and suspensory motions usually coincide because of functional links, giving rise to a hybrid form termed *cranio‐suspensory chewing*. Most taxa that show suspensory motions also exhibit some degree of intracranial movement. Thus, cranio‐suspensory chewing is by far more widespread than suspensory chewing without intracranial movements. Kinetic chewing without suspensory movements has not been documented and thus, to date, remains a theoretical concept. Theoretically, supplementary dimensionally complex mandibular movements along the jaw joint could accompany kinetic, suspensory, or cranio‐suspensory chewing. While such combinations remain undocumented, should they be discovered, ‘‐pseudomastication’ might be adopted to replace ‘‐chewing’.

Favouring the approach of using the kinetic potential of the skull instead of merely differentiating between arcuate and more dimensionally complex ways of chewing does not imply that this broad distinction is of no value. Dimensionally complex chewing behaviours extend beyond simple arcuate movements, resulting in more intricate occlusion patterns that can enhance grinding and food breakdown. This potentially improves digestive performance and allows for the consumption of a greater diversity of foods (Takanobu *et al*., 1998; Reilly *et al*., 2001). Hence, the gross distinction between ‘simple’ arcuate and dimensionally complex chewing behaviours remains an exciting avenue to study. However, it is argued that they are best understood functionally and comparatively when applying the system proposed here.

From this perspective, dimensionally complex chewing can be further divided into suspensory, kinetic, ambignathic, and unignathic types. Unignathic dimensionally complex chewing involving elaborate jaw joint movements is best described as functional masticatory behaviour. As mentioned above, functional masticatory behaviours include both mammalian mastication, facilitated by the TMJ, and pseudomastication, which is mediated by the QAJ in non‐mammals.

## VI. DIVERSITY OF VERTEBRATE FOOD PROCESSING

### (1) Cartilaginous fishes (chondrichthyans)

Despite their considerable taxonomical and ecological diversity and their phylogenetic position as the earliest‐branching extant gnathostomes, functional data on food processing in chondrichthyans, which include sharks (Selachii), rays (Batoidea), and chimaeras (Holocephali), remain scarce. This scarcity is undoubtedly due to their often relatively large body sizes, the difficulty of maintenance in captivity, their scarce or remote distributions, and thus problems in sampling, ultimately resulting in their infrequent availability. Common wisdom dictates that at least sharks do not process their food but rather use bites to incapacitate, subdivide, or dislodge large or complex food items. This notion is likely a contributing factor to the prevailing data scarcity.

From an anatomical perspective, many sharks and rays possess jaw suspensions that enable mobile and protrusible jaws (Frazzetta & Prange, 1987; Wilga & Motta, 1998; Wilga *et al*., 2001; Wilga, 2005). However, as noted above, jaw suspensions often do not form reliable proxies for inferring jaw movement potential. Nevertheless, the hyostylic jaw condition of most chondrichthyans enhances the likelihood of evolving jaws that are moveable with respect to the neurocranium. These individually moveable upper and lower jaws typically work together during feeding. Thus, many rays and sharks can process their food using ambignathic bites. Biting has been hypothesised to be governed to a greater extent by upper jaw movements in sharks and, by contrast, by lower jaw movements in batoids (Dean, Wilga & Summers, 2005); however, exceptions to this proposed dichotomy exist. The extinct *Helicoprion* sp. likely used its lower jaw tooth whorl to ‘saw’ through food (Ramsay *et al*., 2015). Interestingly, some studies hint at fascinating diversity in chondrichthyan food processing. Aside from reports of some sharks repeatedly biting prey or carrion (Tricas & McCosker, 1984), which likely resemble ambignathic ingestive bites, there is growing evidence challenging the common assumption that chondrichthyans do not chew.

In sharks, a distinction has been made, based on electromyography and high‐speed video data, between post‐capture ‘crushing’ biting, ram capture, and suction transport in the durophagous crab‐feeding specialist hammerhead *Sphyrna tiburo* (Wilga & Motta, 2000). This study demonstrated that food processing is part of the behavioural repertoire and showed that jaw‐adductor activity (measured by electromyography, EMG) persisted well beyond visible mouth closure in processing cycles, likely indicative of crushing action. Additionally, it has been shown that white‐spotted bamboo sharks (*Chiloscyllium plagiosum*) process their food and that they, at least during suction feeding, display long‐axis rotation of their hemimandibles, suggesting that chewing may be more dimensionally complex in certain sharks than previously thought (Scott, Brainerd & Wilga, 2022). Implantation of EMG electrodes bilaterally into several jaw closing muscles (adductors) revealed asynchronous muscle activation during food processing across three sharks and one skate species (Gerry *et al*., 2008, 2010). These data suggest that, whereas durophagous species with a fused interramal joint tend to activate muscles on both sides of the jaw synchronously during processing (potentially indicating ambiarcuate chewing), there is more asynchrony in generalist consumers of diverse prey with a flexible intraramal connection (likely indicating interramal motions) (Gerry *et al*., 2008, 2010).

Skates and rays usually possess loose, hyostylic jaw suspensions, flexible mandibular syndesmoses, and relatively flexible jaw joints, which seem to enable a variety of jaw movements (Kolmann *et al*., 2016; Laurence‐Chasen *et al*., 2019). Furthermore, ray food processing appears to be functionally decoupled from food capture, which is governed by suction generated by the ‘anterior disc’, formed by the fused pectoral fins (Kolmann *et al*., 2016). This decoupling is a fascinating analogue to how pharyngeal jaws (see Section VI.2.*c*) decouple the oral jaws from food procurement in many bony fishes (Liem & Sanderson, 1986). In addition, movements of the entire mouth relative to the cranium *via* a loose jaw suspension (unisuspensory motions) can expand the oropharyngeal volume, thereby additionally facilitating fluid movement generation that aids in the transport of food within the oral cavity (Laurence‐Chasen *et al*., 2019).

The little skate (*Leucoraja erinacea*) shows unilateral jaw adductor muscle activation in nearly 50% of its processing cycles, driving the left and right jaw rami independently in a transverse mediolateral motion (Gerry *et al*., 2008), possibly akin to working–balancing side recruitment in herbivorous mammals. An XROMM (X‐ray Reconstruction of Moving Morphology) study of the generalist ocellate river stingray (*Potamotrygon motoro*) highlighted the function of the intricate gliding‐saddle jaw joint (4 in Fig. 5A) during chewing involving arcuate, or ‘compressive’ and longitudinal, or ‘shearing overbite’ jaw movements (see http://movie.biologists.com/video/10.1242/jeb.197681/video‐2 and http://movie.biologists.com/video/10.1242/jeb.197681/video‐3) (Laurence‐Chasen *et al*., 2019). In rays, these distinct compression and overbite shearing cycles of the jaw crush and shear prey alternatingly. This study also demonstrated intraoral transport and manipulation of food using finely controlled water flows, as previously hypothesised (Dean *et al*., 2005) and akin to tongue function in lungfishes (Bemis, 1986; Bemis & Lauder, 1986). The detailed data from freshwater stingrays (Kolmann *et al*., 2016; Laurence‐Chasen *et al*., 2019; Rutledge *et al*., 2019) indicate they apply ambignathic pseudomastication as well as ambiarcuate chewing.

Chimaeras (Holocephali) possess a derived type of autostylic jaw suspension where the upper jaw (palatoquadrate) is firmly fused to the chondrocranium, in theory rendering them excellent subjects for comparative studies on feeding. However, most chimaeras inhabit depths of 200–2500 m, complicating functional studies. Thus, there are currently no functional studies on chimaera feeding. However, our observations from video recordings of chimaeras feeding, combined with the anatomical findings from microcomputed tomography (μCT) scans (see Table S1), suggest that they apply arcuate chewing. Extant chimaeras are often durophagous feeders (Mauchline & Gordon, 1983; Albo‐Puigserver *et al*., 2015) that potentially benefit from strong arcuate chewing bites. Given the apparent hinge‐like jaw joint found in some stem‐group holocephalans (Pradel *et al*., 2021; Dearden, Herrel & Pradel, 2023) and the suggestion that they may be durophagous (Zangerl & Case, 1973), holocephalans may have developed arcuate chewing early in their evolutionary history.

In summary, the available data on chondrichthyans suggest that their food processing involves complex movements of mandibular arch elements (except in chimaeras), the hyobranchial apparatus, the jaw suspension, and the food. Their behavioural repertoire may consequently reflect the condition that arose at base of gnathostomes. Analyses of chewing rhythmicity and the sequencing of chew cycles into distinct fast and slow opening and closing phases also suggest that these traits evolved with jaws and the rise of gnathostomes themselves (Richard *et al*., 2023). Considering that jaws are thought to have arisen from relatively flexible and mobile pharyngeal arches, our findings suggest that dimensionally complex chewing – such as ambignathic pseudomastication – likely represents the ancestral gnathostome processing behaviour.

### (2) Ray‐finned fishes (actinopterygians)

Bony, ray‐finned fishes are exceptional with respect to oropharyngeal processing by having evolved at least three different ‘centres’ for food processing in their oral, buccal, and pharyngeal regions: (*i*) mandibular arch jaws for chewing, (*ii*) specialised hyoid arch elements for raking (often referred to as ‘tongue‐biting’), and (*iii*) complex branchial arch or pharyngeal jaws for pharyngeal processing (‘pharyngeal chewing’) which may involve food grinding, milling, or winnowing.

#### (a) Chewing using mandibular arch jaws

Food processing in early branching actinopterygians, specifi cally in *Polypterus* (bichir), *Lepisosteus* (gar), and *Amia* (bowfin), was described by Lauder (1979, 1980a). Whilst analyses of food processing were secondary to analyses of prey capture, kinematics and EMG recordings were collected to demonstrate that all three taxa use bilaterally asynchronous muscle activation during chewing, in a startling parallel to the above‐mentioned pattern among chondrichthyans. The asynchronous adductor muscle activity and the mobility of the jaw suspension potentially indicate the presence of more complex chewing behaviours in *Polypterus*, *Lepisosteus*, and *Amia*. The relatively simple kinematics approach revealed only arcuate chewing movements, combined with cranial motions along the functional neck. However, the relatively complex skull anatomy of these taxa, which includes multiple moving elements when compared to most tetrapods, as well as unfused interramal joints, suggests the presence of additional subtle motions beyond arcuate chewing. Thus, they might mainly apply arcuate movements with slight intracranial or suspensory contributions, which are likely to be strongest in bowfins, which display highly moveable suspensions and maxillary bones (Lauder, 1979). If present, this would indicate cranio‐suspensory chewing. Recent studies demonstrate that gars exhibit more kinetic skulls than previously anticipated (Lemberg, Shubin & Westneat, 2019), which seems to support the idea of more complex chewing behaviours in early‐branching actinopterygians. However, advanced kinematic studies (e.g. XROMM) are needed to understand their chewing behaviour better. Regardless, recent findings suggest that chewing behaviours enable bichirs to effectively break down food (Sataeva & Kasumyan, 2022; Richard *et al*., 2023). Given the frequent contribution of cranial elevation to suction feeding in fish (Carroll & Wainwright, 2006; Camp & Brainerd, 2013), cranial elevation *via* motions of the functional neck possibly also plays a vital role during chewing in fish. Thus, many chewing osteichthyans may as well be considered functionally ambignathic. The chondrosteans (sturgeons and paddlefish) are of particular interest, having independently developed flexible hyostylic jaw suspensions similar to those found in stingrays and are theoretically capable of true ambignathic chewing.

Teleosts, as the sister taxon of Holostei (gars and bowfins), are commonly known for their complex cranial movements and highly mobile jaws. Contrary to previous assumptions, chewing seems to occur more extensively among teleosts (Gintof *et al*., 2010), and certain species have evolved strong bites capable of reducing food items (Huby *et al*., 2019; Huby, 2021; Cohen *et al*., 2022). EMG recordings from the pinktail caracin (*Chalceus macrolepidotus*) demonstrate that the motor activity pattern of food processing can be highly rhythmic in teleost fishes (Lauder, 1981). More detailed XROMM analyses are ongoing for a particular group of teleosts. Serrasalmids, which include pacu, piranha, and the silver dollar, evolved diverse feeding habits and oral morphologies, including predominantly frugivorous and granivorous pacus (herbivores) with molariform teeth (Cohen *et al*., 2022) and the mainly piscivorous piranhas (carnivores) with caniniform teeth (Huby, 2021). Pacu, for example, exhibit a typical hinge‐like jaw joint that only enables simple open–close movements. However, they also possess a peculiar neurocranial hyomandibular joint (i.e. the joint that attaches their jaw suspension to the neurocranium) with a rounded articular surface at the hyomandibula. Similar to the function of this articulation in biting marine angelfishes (Konow & Bellwood 2005), this type of jaw suspension permits rostro‐caudal rotation of the suspensorium (i.e. suspensory motions), translating into antero‐posterior shifts of the lower jaw (Lomax & Brainerd, 2020, 2021). While more research is needed to confirm if such rostrocaudal suspensory movements occur during pacu chewing (which would indicate suspensory chewing), it may be that antero‐posterior shifts of the lower jaw help adjust the occlusal positioning of the upper and lower jaw to foods with distinct mechanical properties for arcuate chewing (Lomax & Brainerd, 2021).

However, as initially indicated, teleosts differ from ancestral ray‐finned fishes that possess comparatively few mobile cranial elements (Allis Jr, 1922; Lauder, 1980a) in having multiple complex and flexible skull linkage systems (Hildebrand *et al*., 1985; Hanken & Hall, 1993c; Muller, 1996). This remarkable skull complexity and flexibility enables teleosts to apply high‐performance suction feeding due to enhanced volume expansion of the buccal cavity. In addition, teleosts evolved maxillary bones or mouths that could be protracted towards the prey (i.e. jaw protrusion) (Motta & Jay, 1984; Osse, 1985; Westneat & Wainwright, 1989; Bellwood *et al*., 2015), see also https://youtu.be/fqxvd9XAFsI and https://youtu.be/pDU4CQWXaNY. While jaw protrusion aided effective food capture, it usually trades off with mandibular processing, because of conflicting demands on the cranial musculoskeletal system (Barel, 1982; Bouton *et al*., 1999; Sibbing & Nagelkerke, 2000). Thus, the teleostean feeding apparatus usually displays a trade‐off between enhanced jaw protrusion and effective mandibular processing, with powerful ‘biters’ showing limited or no jaw protrusion and extreme ‘protruders’ possessing weaker jaws. To offset diminished mandibular processing, some teleosts seem to have evolved adaptations such as gizzards (Arnette *et al*., 2024), intramandibular joints (Konow *et al*., 2008), “tongue‐bite” apparatuses (Konow & Sanford, 2008a), and pharyngeal jaws (Wainwright *et al*., 2012). By contrast, other teleosts enhanced their suction forces relying largely on hyobranchial depression (Van Wassenbergh *et al*., 2007) instead of jaw protrusion. This enabled these species to combine both powerful suction feeding and effective mandibular processing.

To better understand the evolution of novel processing mechanisms among teleosts, we examined the functional morphology of early‐diverging teleost groups (see Table S1). We determined that osteoglossomorphs generally display hyobranchial processing centres, often lacking protrusible maxillary bones. Conversely, Elopiformes, as early‐diverging elopomorphs, seem to possess protrusible maxillary bones but lack hyobranchial processing centres. For instance, the ladyfish (*Elops saurus*), which consumes small fish, has relatively weak jaws and lacks a hyobranchial processing centre, suggesting that it swallows its food whole and largely unreduced. However, among the early‐branching albuliform elopomorphs, *Albula vulpes* appears to have convergently evolved a hyobranchial processing centre to handle hard‐shelled prey. In early‐branching Otocephala, the presence of protrusible maxillary bones and a remodelled hyobranchial system aids in filter feeding. Combining these findings with the phylogeny of these early‐branching teleosts, it seems likely that maxillary protrusion and either suction‐ or filter feeding, without mandibular processing, may have arisen at the origin of teleosts. The lack of oropharyngeal processing capabilities likely constrained these fish to target smaller prey. This interpretation aligns with the observation that two major innovations central to actinopterygian evolution – protrusible jaws and large teeth – are functionally and evolutionarily incompatible, as the presence of one seemingly trades off against the other. Specifically, highly protrusible jaws occur principally in species with small teeth (Peoples, Mihalitsis & Wainwright, 2025), and small teeth coupled with weak jaws would preclude the effective processing of large prey.

Based on limited fossil evidence, earlier research suggested that the ancestral condition among teleosteomorphs closely resembled that of holosteans (Schaeffer & Rosen, 1961). This suggested a predatory lifestyle characterised by suction feeding combined with the ability to bite and potentially chew prey. However, more recent investigations utilising fossil evidence propose an alternative scenario. These studies indicate that teleosteomorphs may have originated as diminutive suction feeders with a diet predominantly composed of plankton (Arratia & Schultze, 2024). This hypothesis, which aligns with our findings, is intriguing since the rise and diversification of teleosts coincided with a period marked by the decline or extinction of various other aquatic taxa. Notably, many groups experiencing species losses were predators, suggesting that intense competition in the aftermath of the Permian–Triassic extinction event may have contributed to their decline. Teleosts, possibly relying on small invertebrate prey or adopting filter‐feeding strategies during their early evolution, may have initially prospered by exploiting distinct nutritional resources. The evolution of diverse oropharyngeal processing behaviours likely facilitated access to larger and potentially more challenging food sources. This could have significantly fuelled the rapid radiation of early teleosts, ultimately enabling them to emerge as the largest and most diverse vertebrate group. These propositions remain speculative, and further studies on early actinopterygians and other contemporary aquatic species are necessary to develop a comprehensive understanding.

Regardless, certain teleosts have evolved a functional morphology that facilitates both effective suction feeding and the ability to perform chewing bites, thereby circumventing the need to evolve novel oropharyngeal food‐processing behaviours (Van Wassenbergh *et al*., 2007; Ferry‐Graham & Konow, 2010).

#### (b) Raking using the hyoid arch or ‘tongue’ bite

A distinct second set of ‘jaws’, comprising the tooth‐bearing secondary ‘lower jaw’ (the basihyal, resembling a bony tongue) and the opposing dentate secondary ‘upper jaw’ formed by bones of the skull base or palate, has clearly evolved convergently in two clades: the ancient osteoglossomorphs (Sanford & Lauder, 1989, 1990; Frost & Sanford, 1999) and the phylogenetically more derived and geologically younger salmonoids (Sanford, 2001; Camp, Konow & Sanford, 2009). This distinctive set of jaws is commonly known as the ‘tongue bite apparatus’. The ‘tongue bite’ follows initial ingestion of food and involves the tooth‐bearing basihyal moving posteriorly in a raking manner over the food and against the dentition of the neurocranium, hence the designation ‘raking’. This raking behaviour is classified within the broader category of hyoideal processing.

The kinematics of raking are diverse within and among the two groups with varying contributions of neurocranial (epaxial) and pectoral girdle (sternohyoid, hypaxial) input for food processing. The typical kinematics profile of the raking power stroke is perhaps best described as a prey capture strike with the mouth closed around the food (Konow & Sanford, 2008a). Regardless of the kinematic diversity, the motor pattern responsible for driving the raking power stroke in these distantly related groups appears convergently derived from the strike and not from the chewing motor pattern (Konow & Sanford, 2008b). The presence of a third possible origin of raking‐like food processing in anabantoid (labyrinth) fishes has been proposed (Liem, 1963). However, a functional analysis of the Siamese fighting fish (*Betta splendens*) provided evidence of kinematics traits that were homologous (neurocranial elevation), but also some that diverged from raking (protraction instead of retraction of the hyoid during neurocranial elevation) (Konow *et al*., 2013).

#### (c) Branchial processing using branchial arch jaw(s)

The caudal‐most of the three anatomical processing centres in bony fishes consists of modified elements (either fused or unfused) of the branchial basket that form pharyngeal jaws. It has been suggested that the function of more unmodified pharyngeal jaw systems is mainly food transport (Lauder, 1983). By contrast, in more derived systems, the critical function clearly is processing, including pharyngeal grinding and milling. Two major anatomical solutions to branchial processing may be distinguished (Wainwright *et al*., 2012). (*i*) Intrabranchial processing (including pharyngognathy), which involves the fifth ceratobranchials (fused or closely sutured in pharyngognathous species) forming the ‘lower pharyngeal jaw’, which is connected *via* a muscular sling to and opposes the ‘upper pharyngeal jaws’, typically formed by the third pharyngobranchials that often articulate with apophyses of the cranial base. (*ii*) Branchio‐palatal processing (or misleadingly referred to as ‘pharyngeal mastication’), features strong, independently derived fifth ceratobranchials with grinding teeth opposing a postero‐ventral neurocranial plate. Considering that the pharyngeal jaw elements are suspended in a muscular sling, moving the jaws in complex ways during food handling, combined with the deep position of this jaw apparatus means, that kinematics studies conducted without X‐ray video (videofluoroscopy) imaging should be considered with caution and are therefore not included here.

Combined videofluoroscopy and EMG demonstrated that intrabranchial processing involves asymmetrical pharyngeal jaw movements (in haplochromine cichlids) (Liem, 1978), as well as greater upper than lower jaw movements in the amphibious *Periophthalmus* (Sponder & Lauder, 1981). Further, the pharyngognathous dentition varies considerably, with tooth morphology to some extent reflecting diet (Liem, 1978). By contrast, cyprinodontiform fishes exclusively move their unfused lower pharyngeal jaws against a dorsal keratinous ‘chewing pad’ during branchio‐palatal processing (Sibbing, 1982; Gidmark *et al*., 2013), with carp displaying little to no evidence of asymmetric pharyngeal jaw movements (Sibbing, 1982). Pharyngeal jaws in minnows, including carp, lack bony articulations, yet the force production for crushing is limited by muscle length as dictated by food size and pharyngeal gape expansion (Gidmark *et al*., 2013). Carp pharyngeal jaws move highly rhythmically and, in some species, with precise interdigitation of dentition on the two lower jaws, analogous to mandibular movements and teeth in masticating mammalian herbivores (Gidmark *et al*., 2014). Consequently, the term ‘pharyngeal mastication’ has been proposed. Nevertheless, since mastication is strictly defined as a mammalian behaviour, this terminology is potentially misleading and is best avoided.

In summary, current analyses of food processing in bony fishes indicate that rhythmic and repetitive cycles are present in all three recognised processing centres. Among these, arcuate chewing likely reflects the ancestral condition as observed in early‐branching actinopterygians. However, further studies are needed to elucidate the potential presence of more complex cranial movements that could qualify as kinetic, suspensory, or cranio‐suspensory chewing. Regardless, teleosts evolved complex kinetic skulls that significantly enhanced suction feeding capabilities, and early teleosts appear to have shifted away from mandibular chewing due to the trade‐off between kinetic skulls and strong biting force. This trade‐off seems to explain why chewing appears less prevalent among teleosts than many other gnathostome groups. Initially potentially limited to small prey or filter feeding, developing novel processing centres and strategies may have been crucial for these fish, enabling them to subdue larger prey and improving digestion. This potentially led to the emergence of innovative forms of oropharyngeal food processing within this clade, including diverse forms of hyobranchial processing.

### (3) Non‐tetrapod sarcopterygians

Two extant lineages of aquatic‐feeding anamniote representatives of the Sarcopterygii include lungfishes (Dipnoi) and coelacanths (Actinistia).

#### (a) Lungfish (Dipnoi)

Extant lungfishes include one South American (*Lepidosiren paradoxa*), four African (*Protopterus* spp.), and one Australian (*Neoceratodus fosteri*) species, of which only the former two have been subject to peer‐reviewed functional studies (Bemis, 1986; Bemis & Lauder, 1986; Whitlow *et al*., 2022; Richard *et al*., 2023), while the relatively extensive considerations of *Neoceratodus* feeding lack peer review (Perkins, 1972). Given the comprehensive examination of Perkins' (1972) doctoral dissertation, the application of video fluoroscopic studies across all three extant genera, and the combination of Perkins's findings with their own by Bemis & Lauder (1986) to illuminate the evolution of lungfish feeding, we consider the conclusions presented by Perkins (1972) to be valid. The combined findings from these authors suggest that the ancestral condition in lungfish was arcuate chewing (Perkins, 1972; Bemis & Lauder, 1986). However, *Neoceratodus fosteri* seems to have evolved hemimandibular long‐axis rotation and longitudinal jaw movements during occlusion enabled by their flexible jaw‐ and interramal joints (indicating pseudomastication) (Perkins, 1972). Anatomical studies (as well as anatomical observations from μCT scans) confirm that *Neoceratodus fosteri* may use complex (longitudinal, transverse, and/or rolling) jaw movements during food processing (Bemis, 1986). Further anatomical considerations suggest convergent evolution of a jaw depressor muscle analogous to that of tetrapods (Bemis, 1987). Additionally, EMG data suggest an important role of the hyoid muscles by indicating that the hyoid is retracted during gape opening, in contrast to the pattern in chewing mammals of hyoid protraction during gape opening (Konow *et al*., 2011). Recent videofluoroscopy studies show that chewing in *Protopterus* spp. is highly rhythmic and involves prolonged chew sequences of 60–80 cycles, analogous to mammalian mastication (Richard *et al*., 2023). However, lungfishes remain an understudied clade, especially considering their critical phylogenetic position as the closest sisters of tetrapods and their cranial morphological specialisations for food processing.

#### (b) Coelacanths

The extant coelacanths (genus *Latimeria*) are cryptic deep‐water cave dwellers and, hence, poorly studied from an *in vivo* functional perspective. Limited to few interpretations, reports, and observations (Dutel *et al*., 2013, 2015a,b), all that is known is that their feeding may be aided by flexion at the intracranial joint and movements along the jaw suspension (Dutel *et al*., 2015a,b) as well as movement of the hyoid (Lauder, 1980b). While it has been suggested that coelacanth could chew their food (Lauder, 1980b), stomach content analyses indicate that chewing might be limited to a few bites (Uyeno, 1991). Nevertheless, should *Latimeria* engage in chewing behaviours, they likely employ either arcuate‐ or cranio‐suspensory chewing, considering their mobile skull and use of kinetic suction feeding and biting (Dutel *et al*., 2013, 2015a,b).

Despite the targeted development of deep deployable multi‐camera systems with high‐speed recording capabilities for studying coelacanth behaviour (Décamps *et al*., 2017), we lack *in vivo* data for coelacanth food capture and intraoral processing (if present). As outlined above, the sister taxon (Dipnoi) processes food vigorously, and we consider it likely that coelacanths do as well, even if to a limited extent. Baited remote underwater (BRUW) camera traps could perhaps collect functional data on food processing from coelacanths in the wild.

In summary, available data on non‐tetrapod sarcopterygians suggest that food may be processed relying primarily on arcuate chewing or cranio‐suspensory chewing. While the coelacanths seem to have maintained at least one kinetic movement along the intracranial joint as well as suspensory motions, lungfish seem to have lost all mobility in their cranium connected to the simplification of dermal elements of the skull and hence usually apply arcuate chewing. However, the Australian lungfish (*Neoceratodus fosteri*) seems to have evolved jaw‐ and interramal joints, enabling pseudomastication. Given that the dentition of *Neoceratodus fosteri* shares a similar morphology with extinct early‐branching lungfish (Rieppel, 2023), pseudomastication may be argued to have been the ancestral processing strategy in lungfish. However, osteological evidence from the jaw joint does not appear to corroborate this hypothesis (Miles, 1977; Zhu & Yu, 2002), but rather supports arcuate chewing as the ancestral processing behaviour.

### (4) Tetrapods: lissamphibians

#### (a) Caecilians (Gymnophiona)

Modern caecilians feed on small fossorial or aquatic creatures, including earthworms (Loveridge, 1936; Wake, 1980; Maciel *et al*., 2012; Kouete & Blackburn, 2020), termites (Barbour & Loveridge, 1928; Loveridge, 1936; Hebrard, Maloiy & Alliangana, 1992; Nussbaum & Pfrender, 1998), arthropods, their larvae, and shrimps (Wake, 1978; Moodie, 1978), as well as various other small invertebrates (Kupfer, Nabhitabhata & Himstedt, 2005; Kouete & Blackburn, 2020). Their skull is roughly torpedo‐shaped (resembling a circular paraboloid) (Sherratt *et al*., 2014) and often displays relatively large teeth and a reduced number of or generally fused cranial and lower jaw (‘mandible’) elements (Hanken & Hall, 1993b). These skull properties diverge from other extant amphibians and are often attributed to their ancestral burrowing (fossorial) lifestyle (Wake, Hanken & Hall, 1993; O'Reilly, 2000). While their divergent skull morphology renders them excellent candidates for functional morphological feeding studies, their cryptic burrowing lifestyle complicates these studies. Until today, visible‐light video recordings have been primarily used to study caecilian feeding behaviours, with fluoroscopic recordings still being scarce (Herrel & Measey, 2012). Consequently, caecilian feeding remains poorly understood, and only outlandish features of their feeding behaviour have been studied in any detail (Parker, 1956; Wake, 1977; Kupfer *et al*., 2006; Wilkinson *et al*., 2008).

It is well‐known that caecilians evolved powerful rotational feeding, during which they grasp the prey with their jaws and rotate their bodies to dismantle or reduce oversized prey against the substrate (i.e. often the confined walls of their burrows) (Tanner, 1971; Bemis, Schwenk & Wake, 1983; Measey & Herrel, 2006; Herrel & Measey, 2012; Herrel *et al*., 2019). This behaviour reduces food to a manageable size that is easily digested (Measey & Herrel, 2006) and may be considered more effective than the processing behaviours of many other amphibians. However, detailed feeding studies from Bemis *et al*. (1983), which included high‐speed videography, EMG, X‐ray photography, and anatomical considerations, revealed that *Dermophis mexicanus* also performs consecutive arcuate chewing. Hence, chewing may be more common amongst caecilians than previously thought. However, only earthworms were fed, which often resulted in partial ingestion. Therefore, the animals are required to ingest, process, and transport the food either by alternating sequences that prevent more cyclic chewing or by mixed feeding stages that result in ‘gulping bites’, as argued previously (Schwarz *et al*., 2023), which we here refer to as ingestive bites. While considerations of online video references seem to indicate that *Ichthyophis kohtaoensis* may also apply arcuate chewing, *Typhlonectes natans* does not seem to apply oropharyngeal food processing (see Table S1). Therefore, more studies are needed to better understand food processing among caecilians.

In theory, pleurokinesis, which has been suggested to be present in caecilians (Marcus, Stimmelmayr & Porsch, 1935; Wake & Hanken, 1982; Summers & Wake, 2005), could assist rotational feeding and chewing as it has been argued to maintain a steady bite force across a wide range of jaw gapes in caecilians (Kleinteich, Haas & Summers, 2008; Herrel *et al*., 2019). If pleurokinesis were present during caecilian chewing, the behaviour would qualify as cranio‐suspensory chewing. However, the detailed feeding studies from Bemis *et al*. (1983) revealed that *Dermophis mexicanus* does not seem to exhibit pleurokinesis. These results may suggest that only slight movements of the jaw suspension (quadrate/squamosal unit) are possible and that these may be more important for vibrational sound conduction from the mandible to the inner ear *via* the synovial connection between the columella (stapes) and the jaw suspension.

In summary, it could be argued that the ancestral processing condition in caecilians might have involved arcuate chewing, as seen in certain extant caecilians. The absence of observed chewing behaviours in some species might be attributed to their burrowing lifestyle and the fact that only certain foods may trigger chewing. Conversely, because of their lifestyle, preferred prey, and reliance on rotational feeding, caecilians may have discarded chewing behaviours, with only certain species having maintained or re‐evolved cyclical mandibular processing behaviours. Therefore, more studies are needed to determine whether chewing is ancestral to the caecilian clade.

#### (b) Salamanders (Caudata)

Many salamanders display metamorphosis, a developmental strategy in which the aquatic larval stage rapidly develops into (more) terrestrial adults. This shift not only impacts the environments inhabited but also the general morphology of the feeding apparatus (Rose, 2003; Schwarz *et al*., 2020b). These morphological changes, in turn, seem to facilitate or necessitate functional shifts in food‐processing behaviour (Schwarz *et al*., 2020b, 2023). In addition to metamorphosis, salamanders have evolved divergent developmental strategies, referred to as direct development and paedomorphosis (Bonett & Blair, 2017; Bonett *et al*., 2021; Bonett & Ledbetter, 2022). These developmental differences and the resulting diverse yet developmentally somewhat comparable morphotypes are associated with different processing behaviours (Schwarz *et al*., 2023).

Salamanders apply several forms of intraoral food processing, including tongue–palate rasping (a form of hyobranchial processing), arcuate chewing, as well as pseudomastication (Schwenk & Wake, 1993; Heiss *et al*., 2019; Schwarz *et al*., 2020a,b,c, 2021, 2023). Some salamandrid newts, for instance, pass through developmental stages involving all three distinct forms of intraoral food processing, including pseudomastication in early larval morphotypes (Schwarz *et al*., 2020c), arcuate chewing during later larval or neotenic life, and tongue‐palate rasping after metamorphosis (Schwarz *et al*., 2023). Whereas some paedomorphic species display an early larval morphology and dimensionally complex chewing behaviours throughout life (Schwarz *et al*., 2020a, 2021), others may metamorphose to display a post‐metamorphic morphology and apply tongue–palate rasping (Schwarz *et al*., 2023).

Studies on the feeding behaviour of the hellbender (*Cryptobranchus alleganiensis*) and the Japanese giant salamander (*Andrias japonicus*) suggest the presence of more dimensionally complex hemimandibular movements (Cundall *et al*., 1987; Matsumoto *et al*., 2024). The combination of a flexible mandibular symphysis and a saddle‐like jaw joint enables giant salamanders to feed using arcuate open–close movements as well as hemimandibular roll (long‐axis rotation) (Matsumoto *et al*., 2024). Hence, as suggested earlier, giant salamanders may bite or chew using asymmetric mandibular movements, where one side of the lower jaw locks the prey against the upper jaw while the other side bites down (Cundall *et al*., 1987). This behaviour would qualify as a novel form of dimensionally complex bites or pseudomastication based on interramal motions (specifically long‐axis rotation). However, since giant salamander feeding remains relatively poorly studied, more evidence is needed to confirm these ideas. Nevertheless, the presence of such pronounced hemimandibular movements through a flexible symphysis in a family of salamanders may suggest that similar behaviours are more common than previously thought. Most salamanders have unfused hemimandibles, which may allow for similar behaviours that may have gone unnoticed due to the relatively small body size of many salamanders. Interestingly, the complex chewing behaviour in sirenid salamanders, while different from that proposed for giant salamanders, also relies on a flexible symphysis (Schwarz *et al*., 2020c, 2021). In addition to the three main types of prey processing, some salamander species display rotational feeding and prey‐shaking behaviours (Lindquist & Bachmann, 1980; Lukanov *et al*., 2016; Schwarz *et al*., 2023).

In summary, salamanders exhibit chewing behaviours when in their aquatic phase, characterised by a larval morphology featuring a hyobranchial apparatus adapted for gill respiration and suction feeding. These behaviours can be complex (pseudomastication) in early larvae and some paedomorphic forms, transitioning to less complex, arcuate chewing in later larval stages or paedomorphic salamanders with a more developed larval morphology. However, upon metamorphosis, salamanders develop a hyolingual apparatus, or ‘true’ tongue, adapted to assist lung respiration through pulse pumping (Brainerd, 1998; Brainerd & Owerkowicz, 2006), lingual prehension for prey capture (Reilly, 1996), and potentially terrestrial food processing (e.g. tongue–palate processing or chewing) (Richard *et al*., 2023; Schwarz *et al*., 2020b, 2023). Considering that metamorphosis is thought to be ancestral to lissamphibians (Reiss, 2002; Schoch & Witzmann, 2024), tongue–palate rasping may be argued to be the ancestral ‘adult’ food‐processing condition in salamanders, which was subsequently lost in several non‐metamorphosing groups. However, this perspective may be misleading, as the type of metamorphosis involved resulted in adult morphotypes that did not seem to possess true tongues, but rather cartilaginous hyobranchial systems (Ivakhnenko, 1978; Skutschas & Martin, 2011; Rong, 2018; Schoch, Werneburg & Voigt, 2020; Jones *et al*., 2022) incapable of tongue–palate rasping. Given the palaeontological evidence, and the fact that the hyobranchial systems of adult early‐diverging salamanders (cryptobranchoids) appear less adapted for tongue–palate rasping (Schwarz *et al*., 2023), this food‐processing behaviour seems to have either arisen among the stem group of later‐diverging salamanders (salamandroids) or emerged multiple times within this group. Regardless, while more detailed studies are required to pinpoint the emergence of tongue–palate rasping in salamanders, the ancestral aquatic food‐processing condition among salamanders seems to have been chewing, either arcuate or dimensionally complex (pseudomastication).

#### (c) Frogs (Anura)

While most adult frogs use sticky protractible tongues, jaw prehension, or suction feeding to ingest prey, similar to their lissamphibian relatives (salamanders and caecilians) (Sokol, 1969; Nishikawa, 2000; Cundall, Fernandez & Irish, 2017; Herrel *et al*., 2019), the ingestive feeding behaviour of larval frogs (tadpoles) often deviates (Candioti, 2006; Herrel *et al*., 2019). Most tadpoles either passively filter particles from the water column or scrape the substrate to acquire food particles with their keratinised beaks (Wassersug & Yamashita, 2001; Larson & Reilly, 2003; Bonacci *et al*., 2008; Venesky *et al*., 2011, 2013; Sousa *et al*., 2014), while only few species are known to apply suction feeding (Deban & Olson, 2002). Some species have evolved predatory mouthparts (Grosjean, Vences & Dubois, 2004) and carnivorous lifestyles, preying on small aquatic invertebrates and other tadpoles (Haas *et al*., 2014; Sousa *et al*., 2014). While detailed kinematic descriptions are still lacking, observations (Vera Candioti, 2005; Natale *et al*., 2011) and stomach content analyses (Haas *et al*., 2014) revealed that carnivorous tadpoles use their mouthparts for piecemeal feeding when the prey is too big to be engulfed whole. Specifically, they utilise their jaws to bite or scrape parts from their prey or other food sources. Nonetheless, these studies do not address whether subsequent cyclic mandibular processing behaviours accompany these biting actions once pieces of food are detached. Thus, despite the apparent anatomical capability for chewing, it remains unclear if these carnivorous tadpoles engage in cyclic mandibular processing behaviours.

Additionally, certain scraping and grazing behaviours of tadpoles share significant morpho‐functional similarities with chewing and biting. In all four behaviours, mandibular jaws cyclically contact and damage food. However, while scraping and grazing reduce food, they differ in purpose. Scraping and grazing serve both the ingestion and processing of food. Therefore, mandibular scraping and grazing resemble mixed feeding behaviours (ingestive bites), not chewing. Adult frogs, on the other hand, are fascinating in that they seem to have almost universally lost intraoral food‐processing behaviours and instead often swallow food whole and largely unreduced (Lauder & Reilly, 1994; Nishikawa, 2000; Schwenk, 2000b; Schwenk & Rubega, 2005; Herrel *et al*., 2019). This lack of chewing behaviours might explain why many frogs seem to have lost their dentition: frogs have primarily specialised in feeding on small invertebrate prey, which may not require extensive processing (Paluh *et al*., 2021). However, specialised clawed frogs display extra‐alimentary processing behaviours, using their clawed extremities to position and damage their prey (appendage‐use processing) (Sokol, 1969; Anzeraey *et al*., 2017; Fernandez, Irish & Cundall, 2017; Heiss & Lemell, 2023) (see Table S1).

Processing behaviours in frogs are diverse and separated by their ontogenetic development. Tadpoles either apply ingestive bites to scrape or graze algae or other substrates, filter feed on small particles that do not require processing, or suction feed and bite particles or prey. Given the different food sources, the only taxa that seem likely to chew are the carnivorous suction feeders. However, solid evidence for chewing tadpoles is lacking to date. Most adult frogs swallow food whole and unreduced, while others evolved to apply appendage‐use processing.

### (5) Tetrapods: amniotes

Amniotes, a clade encompassing reptiles and mammals, are distinguished from their amphibian ancestors by several key anatomical adaptations that facilitate their terrestrial lifestyle. These adaptations include a range of features that enhance their feeding capabilities on land, such as: (*i*) skulls structured to enable relatively high bite forces, including strong jaws and crania with elevated snouts; (*ii*) flexible and often more freely moveable necks; (*iii*) relatively large and strong teeth that are anatomically differentiated to suit diverse diets; (*iv*) varied forms of intracranial motions, and (*v*) claws that enable gripping, subduing, and processing of prey. One of the most significant innovations is often considered to be (*vi*) a more mobile, flexible, and accurately manoeuvrable tongue. Compared to amphibians, amniotes seem to have evolved more refined and aligned jaw and tongue movements to facilitate terrestrial feeding, including effective oropharyngeal processing of food.

#### (a) Sauropsids: lepidosaurs (‘scaled lizards’)

Lepidosaurs include the rhynchocephalian tuatara *Sphenodon punctatus* and the squamate lizards and snakes, which evolved distinct and intriguing functional anatomies enabling dimensionally complex jaw movements. Several recent reviews describe what is known about these animals to date (Schwenk, 2000c; Bels *et al*., 2019; Moon *et al*., 2019; Segall *et al*., 2023). Accordingly, our focus mainly centres on the existence and functional morphology of dimensionally complex chewing.

##### (i) Rhynchocephalians

Tuatara, the sole extant member of rhynchocephalians, applies longitudinal (antero‐posterior) chewing behaviours facilitated by a peculiar jaw joint (Gorniak, Rosenberg & Gans, 1982; Jones *et al*., 2012) and particularly oriented jaw adductor muscles (Schaerlaeken *et al*., 2008). Notably, the pterygoideus muscle has a relatively pronounced antero‐posterior orientation and is thought to be responsible for the longitudinal translation of the mandible (Gorniak *et al*., 1982). In addition, multibody dynamics analysis (MDA) suggest that a flexible interramal joint may be essential for enabling adjustments in the angle between the hemimandibles, which, in turn, permit antero‐posterior or longitudinal movements of the mandible (Jones *et al*., 2012). These analyses further suggest that in addition to hemimandibular wish‐boning, long‐axis rotation of the hemimandibles may be a component of the jaw movements (Jones *et al*., 2012). Although *in vivo* studies confirming these theoretical findings are currently lacking, the analyses appear robust and well supported. Consequently, it may be argued that *Sphenodon* exhibits highly repetitive, almost mammalian‐like dimensionally complex chewing behaviours (pseudomastication), characterised by jaw cycles more stereotyped than those observed in lizards (Schaerlaeken *et al*., 2008).

##### (ii) Lizards (non‐serpent squamates)

Certain herbivorous (Herrel, Aerts & De Vree, 1998) and insectivorous lizards (Throckmorton & Clarke, 1981; Handschuh *et al*., 2019) display suspensory motions as seen among various squamates (Metzger, 2002; Herrel *et al*., 2007). Lizards sometimes display relatively complex forms of intracranial motions or ‘cranial kinesis’ when compared to many other tetrapods. These intracranial motions often involve the coupling of suspensory and mesokinetic movements (Frazzetta, 1962). In the Egyptian mastigure (*Uromastyx aegyptia*), suspensory motions seem to have evolved to become a crucial part of chewing (suspensory chewing) and may help in cropping plant matter, after which longitudinal jaw movements further help grind food with precisely occluding teeth (Throckmorton, 1976, 1978, 1980; Herrel & De Vree, 2009). Beyond suspensory motions, intracranial motions (or ‘cranial kinesis’) can further affect oropharyngeal food processing in some lizards, likely giving rise to cranio‐suspensory chewing (Handschuh *et al*., 2019). A simulation of the complex intracranial motions in the European snake‐eyed skink (*Ablepharus kitaibelii*) showed how meso‐ and metakinetic movements may alter the orientation of the occlusal surfaces of the upper and lower jaw relative to one another (http://movie.biologists.com/video/10.1242/jeb.198291/video‐1). However, to date, only a single XROMM study has been conducted with the aim of elucidating the three‐dimensional movements of the different cranial elements (Montuelle & Williams, 2015). In this study, the authors showed that the different types of intracranial motions in *Gecko gecko* are not always coupled (potentially indicating the presence of independent kinetic‐ and suspensory chewing as well as coupled cranio‐suspensory chewing behaviours). Moreover, their data suggest that lower jaw retraction plays a vital role during chewing. Unfortunately, the authors did not quantify out‐of‐plane movements (e.g. planar deformation of the palatine) or long‐axis rotation of the lower jaw, which are likely to occur. Current data indicate that retraction of the snout during food‐processing cycles alters the bite force during the slow close/power stroke phase (Herrel *et al*., 1999b; Herrel, Aerts & De Vree, 2000; Montuelle & Williams, 2015). In any case, suspensory and intracranial motions alter the positioning of the upper and lower jaw, which can also result in dimensionally complex occlusal action. Future three‐dimensional studies are clearly needed to understand intracranial movements better in lizards.

In summary, complex kinetic forms of chewing, such as cranio‐suspensory chewing, seem to be present in certain Scincomorpha, Gekkota, and Anguimorpha. By contrast, certain lizards, such as *Uromastyx aegyptia*, seem to have evolved less kinetic skulls that only permit suspensory chewing.

##### (iii) Snakes (Serpentes)

Snakes are generally believed not to chew, and our considerations support this idea despite considerable efforts to find any exception. Although snakes do not chew, they can use other processing strategies to aid chemical digestion. These behaviours may include tearing off parts of large prey and carrion (grasp‐tearing) (Jayne, Voris & Ng, 2002) or mandibular cutting, crushing, or sawing behaviours (Coleman *et al*., 1993; Mullin, 1996; Dove, Reed & Snow, 2012; Kojima *et al*., 2020). While the mandibular crushing and cutting behaviours qualify as non‐cyclic bites, the sawing behaviour is indeed cyclic and rhythmic. For example, snakes of the genus *Aplopeltura* (blunt‐headed slug‐eating snakes) can process and saw off parts of their snail prey during mandibular sawing (Kojima *et al*., 2020). Consecutive ‘streptostylic’ movements of the quadrate bone (the jaw suspension) aid the mandibular sawing between the recurved dentition, which helps pull in and slice through partially ingested snail and slug prey. Thus, this behaviour depicts a mixture of ingestion, processing, and food transport and, hence, ingestive bites *via* (cranio‐)suspensory motions.

Suspensory motions are also crucial for other snake feeding behaviours (Cundall & Greene, 2000), including mandibular raking that aids the ingestion of insect prey (Iordansky, 1989; Kley & Brainerd, 1999; Kley, 2001). In addition, intracranial movements are generally extremely pronounced in snakes and may involve the supratemporal bone, quadrate, pterygoid, ectopterygoid, vomer, palatine, and maxilla (Gans, 1974). Such intracranial motions are critical to the so‐called ‘pterygoid walk’, which many snakes use to ingest prey (Cundall & Greene, 2000). This peculiar ingestion behaviour can allow snakes to swallow prey larger than the resting size of their head without prior mechanical reduction (Gans, 1974; Jayne *et al*., 2022; Jayne, 2023).

In conclusion, snakes may employ semi‐alimentary processing behaviours such as grasp‐tearing and intra‐alimentary processing, including bites or ingestive bites. Given the anatomical constraints of snakes, whose tongues cannot transport food within the oral cavity during successive mandible‐based processing cycles, it is unsurprising that they do not rely on purely intraoral forms of cyclic mandibular food processing. Instead, they seem to adopt alternative mandible‐based food‐processing mechanisms that are either non‐cyclic or not entirely intraoral.

#### (b) Sauropsids: turtles (Testudines)

Testudines are divided into two groups: Pleurodira (side‐necked turtles) and Cryptodira (hidden‐necked turtles), distinguished by their head‐retraction mechanisms. Their feeding habits vary widely, with most turtles being opportunistic omnivores (Ernst & Barbour, 1989). Terrestrial species tend towards herbivory and mainly consume grasses, leaves, and fruits, while aquatic species lean towards carnivory and often feed on fish, snails, or carrion.

It is generally known that the cranial anatomy of turtles deviates from that of other extant amniotes. Robust and rigid crania and mandibles characterise turtle skulls. Their distinctive cranial morphology is further defined by the absence of a temporal fenestra and the presence of keratinous beaks with sharp edges, evident on both the upper and lower jaws. However, within the turtle lineage, unique adaptations have evolved to aid feeding across water and air and facilitate various dietary specialisations. The alligator snapping turtle (*Macrochelys temminckii*), for instance, uses a worm‐like appendage on its tongue to lure fish, rapidly grasping them with its sharp and pointed beak (Spindel, Dobie & Buxton, 1987; Drummond & Gordon, 2010). Further, the exceptional mata mata (*Chelus fimbriata*) employs powerful suction‐feeding behaviours facilitated by its tubular snout and powerful hyobranchial system (Lemell *et al*., 2002). A closer look at the jaw and beak anatomy reveals that herbivorous turtles generally possess comparatively more robust jaws and beaks, while carnivores tend to exhibit comparatively slender jaws and beaks with more pointed tips (Ponstein *et al*., 2024). Unique among reptiles, leatherback sea turtles (*Dermochelys coriacea*) possess caudally oriented keratinous spines (papillae) covering their entire oropharyngeal cavity and oesophagus (Fraher *et al*., 2010), aiding in the ingestion, processing, and swallowing of their soft‐bodied and potentially toxic jellyfish prey. These adaptations underscore the remarkable diversity of feeding strategies and anatomical specialisations within the turtle lineage, enabling them to thrive across various habitats and dietary niches. Note also that individual dietary preferences may vary based on age, sex, season, and geographic location, with many species transitioning from carnivorous juveniles to herbivorous adults (McCauley & Bjorndal, 1999).

The kinematics of feeding in turtles have been insufficiently explored using X‐ray recordings. Consequently, detailed insights into various feeding behaviours, including chewing, have remained largely limited. However, our literature review and behavioural observations from previously recorded high‐speed videos and online video references revealed multiple forms of mechanical food processing. Appendage‐use processing, using claws or ‘pseudoclaws’ to damage food or prey, has been described for the loggerhead sea turtle (*Caretta caretta*), green sea turtle (*Chelonia mydas*), and the leatherback sea turtle (Davenport & Clough, 1985; Bels & Renous, 1992), but our observations suggest it appears to be universally employed across turtles. The forelimbs of turtles are frequently utilised during processing to grasp and disassemble food items. Tongue‐palate processing, facilitating actions such as shell crushing within the oral cavity, has been suggested to be present in certain aquatic snail‐feeding species (e.g. *Pelusios castaneus*) (Lemell & Weisgram, 1996). Apart from tongue–palate interactions, cyclic, mandible‐based processing (chewing) seems to be prevalent among most turtles, with arcuate chewing commonly being identified (Lemell *et al*., 2019). However, evidence suggests that certain turtles, like the Texas tortoise (*Gopherus berlandieri*), common box turtle (*Terrapene carolina*), and the green sea turtle, engage in chewing motions, involving longitudinal movements of the mandible (pseudomastication) (Bramble, 1974; Iordansky, 1996; Bels, Davenport & Delheusy, 1997; Marshall *et al*., 2014).

**Fig. 7:**
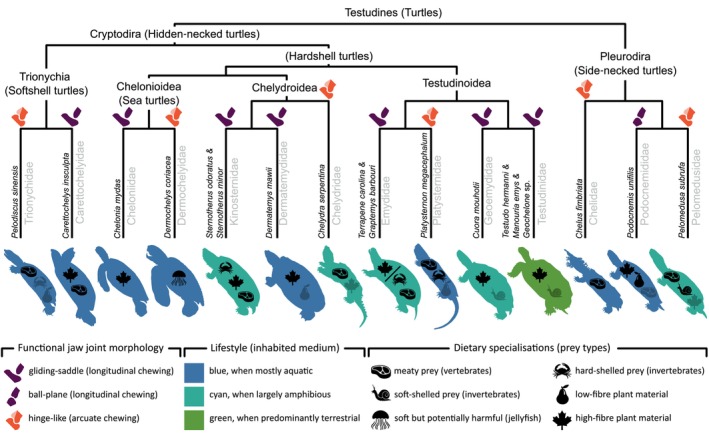
Chewing in turtles. The distribution of the form and function of turtle chewing is displayed in connection to diet and primarily inhabited medium. Primary food sources are arranged from top to bottom within the turtle outlines; the less‐prominent (less‐opaque) items indicate foods that are rarely eaten or are specific to certain regional populations. The slashed primary food items in Emydidae display the difference between the two considered species. To help explain the presence of longitudinal chewing among turtles, we distinguished hard‐shelled prey from soft‐shelled prey, such as insects or softer molluscs like many land snails, in order to separate ‘true’ durophagy from feeding on softer items. Please note that only one or a few exemplary species were considered for each family. The selection of these species was founded on the aim of determining the presence of longitudinal mandible movements across turtle families. Therefore, this approach does not rule out the occurrence of ‘simple’ arcuate chewing in other species belonging to families in which some members have been shown to employ more longitudinal chewing behaviours. Therefore, this overview does not conclusively elucidate the distribution and evolution of chewing behaviours in turtles but instead serves as a preliminary indication. Studied species include **Trionychidae**: *Pelodiscus sinensis* (UF:Herp:52509, MorphoSource ID (ms‐id): 000423509); **Carettochelyidae**: *Carettochelys insculpta* (UF:Field#:49415, ms‐id: 000615515); **Cheloniidae**: *Chelonia mydas* (UF:Herp:51413, ms‐id: 000413162); **Dermochelyidae**: *Dermochelys coriacea* (UF:Herp:84769, ms‐id: 000574728); **Kinosternidae**: *Sternotherus odoratus* (YPM:VZ:YPM HERR 019736.001, ms‐id: 000072565) and *Sternotherus minor* (FMNH:Amphibians and Reptiles:211696, ms‐id: 000042852); **Dermatemydidae**: *Dermatemys mawii* (UF:Herp:84769, ms‐id: 000574728); **Chelydridae**: *Chelydra serpentina* (OUVC:10681, ms‐id: 000076039); **Emydidae**: *Terrapene carolina* (UF:Herp:181844, ms‐id: 000605538), and *Graptemys barbouri* (UF:Herp:61899, ms‐id: 000046483); **Platysternidae**: *Platysternon megacephalum* (UF:herp:191750, ms‐id: 000574697); **Geoemydidae**: *Cuora mouhotii* (Blüml, 2019); **Testudinidae**: *Testudo hermanni* (MVZ:Herp:238087, ms‐id: 000385515), *Manouria emys* (UMMZ:herps:227759, ms‐id: 000070107) and *Geochelone* sp. (MVZ:Herp:233519, ms‐id: 000423820); **Chelidae**: *Chelus fimbriata* (UF:Herp:117469, ms‐id: 000574622); **Podocnemididae**: *Podocnemis unifilis* (UF:Herp:191763, ms‐id: 000605789); and **Pelomedusidae**: *Pelomedusa subrufa* (UF:Herp:85211, ms‐id: 000606215). Functional considerations based on previously recorded high‐speed video footage of turtle feeding from the University of Vienna (see Videos S1–S5), alongside selected online video references from social media (Table S1). Phylogeny is after Crawford *et al*. (2015).

Given the scarcity of comparative anatomical studies on turtle chewing, we analysed previously recorded high‐speed video footage of turtle feeding from the University of Vienna (see online Supporting Information, Videos S1–S5), alongside online videos of at least one representative species from each of the 14 turtle families (see Table S1). Note that this overview only serves as a preliminary consideration (more detailed information is provided in the legend to Fig. 7). We hope that it may spark interest in XROMM studies of turtle feeding, as these are facilitated by the ease of marker implantation on both upper and lower jaw beaks, with the caveat that surgical intervention may be necessary for marking hyobranchial structures. Our *in vivo* feeding observations and video recordings support the notion that various turtles retract the mandible during the final stage of the power stroke, potentially facilitating the cutting and grinding of food between the beak structures of the upper and lower jaws. Contrary to prior assumptions (Iordansky, 1996), the application of longitudinal jaw excursions appears to be less universal, or at least less pronounced in certain turtles, than previously believed.

In addition to our behavioural considerations, we examined the jaw joint anatomy through μCT scans from MorphoSource.org as well as private scans and compiled our findings in Fig. 7. Our results suggest the presence of both hinge‐like jaw joints facilitating arcuate movements and joints with divergent anatomies allowing additional longitudinal (antero‐posterior) movements in both hidden‐necked and side‐necked turtles. Interestingly, the anatomy of jaw joints permitting longitudinal movements appears to differ between these two groups. Hidden‐necked turtles that use longitudinal movements universally exhibit a gliding‐saddle type of jaw joint (for more details, see Figs 5F and 7). The presence of longitudinal chewing and similar jaw joint shapes in some species across all superfamilies suggests it might depict an ancestral trait in hidden‐necked turtles, possibly lost in certain taxa. Conversely, the sole known side‐necked turtle exhibiting longitudinal chewing, the yellow‐spotted river turtle (*Podocnemis unifilis*), possesses a ball‐plane‐like jaw joint akin to that of sirenid salamanders (Schwarz *et al*., 2020c, 2021); compare with Fig. 5D. This morphological disparity, coupled with the unique position of *Podocnemis unifilis* among arcuate chewing side‐necked turtles, suggests a potential evolutionary divergence in jaw joint functionality within this group. Thus, it seems that side‐necked turtles have independently evolved an anatomical solution to enable longitudinal chewing. However, a detailed study of a larger number of species is needed to confirm this assumption.

The emergence of either longitudinal or arcuate chewing within phylogenetic lineages poses an intriguing conundrum. While phylogeny and habitat preference do not clarify jaw joint evolution, dietary habits appear to be a key factor. All types of predominantly carnivorous as well as certain durophagous species generally seem to possess hinge‐like jaw joints and engage in arcuate chewing. By contrast, species that consume a substantial proportion of high‐fibre plant material (high‐fibre herbivory) exhibit jaw joints that enable longitudinal movements, hence pseudomastication. Notably, terrestrial herbivorous turtles, which depend most strongly on plant material, show the most pronounced longitudinal lower jaw deflections, likely aiding in efficiently cutting and grinding plant materials. Although relying on different anatomical jaw joints, among hidden‐necked and side‐necked turtles, high‐fibre herbivorous species use pseudomastication to process their food.

Whether the combination of pseudomastication and high‐fibre herbivory were ancestral or evolved independently within turtles remains a problematic question. However, some studies suggest a herbivorous ancestry in turtles (Lee, 1996; Werneburg & Maier, 2019), which may indicate the ancestral presence of gliding jaw joints and pseudomastication to facilitate effective food breakdown. If pseudomastication and herbivory were ancestral to turtles, this would suggest that lineages adopting less fibre‐rich diets may have substantially reduced or lost their capacity for pseudomastication. Gliding‐saddle jaw joints are only present among hidden‐necked turtles. The exclusive presence of such jaw joints fits the argument that flat articulating surfaces opposing the quadrate are ancestral to hidden‐necked turtles (Evers *et al*., 2023). Conversely, side‐necked turtles may have ancestrally shifted to a more carnivorous diet, leading to the loss of gliding‐saddle jaw joints and pseudomastication. Nevertheless, the primarily herbivorous *Podocnemis unifilis* presents an intriguing example of convergent evolution, as it possesses a distinct anatomical feature – a ball‐plane jaw joint – that enables longitudinal jaw gliding and, consequently, pseudomastication.

Regardless, the functional importance of the combination of pseudomastication and beaks, which results in cutting and grinding motions, appears universal among turtles to enable the consumption of high‐fibre plant materials. The association between longitudinal jaw motion (*via* gliding jaw joints), beaks, and herbivory has long been established (Hotton *et al*., 1986; King, 1994), although this connection was previously challenged (Iordansky, 1996; Rybczynski & Reisz, 2001). While a more detailed and nuanced examination of turtle diet and functional anatomy is essential for definitive conclusions, our findings suggest a connection between high‐fibre herbivory and the presence of gliding jaw joints and longitudinal chewing beaks in turtles.

#### (c) Sauropsids: birds (Aves)

Birds are unique in that they often possess highly efficient gizzards (chewing stomachs), which enable pronounced food breakdown *via* gastric milling that, in certain species, rivals the effectivity of mammalian rumination (chewing the cud) (Moore, 1999). Food processing in birds is generally regarded as taking place predominantly after ingestion, with most breakdown occurring during the post‐oral phase through processes such as passage through the crop, where food is moistened and softened, and subsequent gastric milling within the gizzard (Schwenk, 2000b; Reilly *et al*., 2001; Schwenk & Rubega, 2005; Rico‐Guevara *et al*., 2019; Heiss *et al*., 2023). However, many birds of prey (raptors) utilise their talons to kill, dismember, or puncture prey (appendage‐use processing). In addition, they employ their pointed beaks to tear apart the prey, facilitating ingestion and swallowing (grasp‐tearing). The lack of teeth in birds, in combination with the previously discussed adaptations, has, apart from some divergent interpretations (Bhattacharyya, 2013), led to the commonly held perspective that birds do not chew.

However, relatively recent results from XROMM studies (Dawson *et al*., 2011) and observations from online video references suggest that certain types of dimensionally complex chewing may have evolved in birds (see Table S1). A multitude of anatomical studies prompt the idea that the highly kinetic nature of the bird skull allows for surprisingly complex movements of the upper and lower beak (see below). These movements turn the beak into a multi‐functional tool whose mechanically most demanding task often appears to be feeding. Still, detailed feeding studies focussing on the distinct stages of the feeding cycle or the 3D beak kinematics of various avian species are lacking. In the following, an anatomical background is provided, which, in combination with our observations and literature review, will help answer whether birds process their food with their beaks.

Although the basic construction of the skull is similar in all birds, the vast variability in size and morphology comes with a high diversity in diet and feeding types. Bird diets include seeds, plants, nectar, insects, molluscs, fishes, amphibians, eggs, small mammals, and other birds. As a result, birds employ a wide array of feeding strategies, including pecking, seed cracking, filter feeding, probing, ballistic feeding, as well as both surface and subsurface aquatic feeding, among many others. Many of these require some form of intraoral food processing, such as separating digestible and indigestible parts (e.g. husking of seeds or extracting snails from their shells). These processing behaviours may require complex three‐dimensional jaw movements, which are achieved mainly by a combination of three kinetic features of the avian skull (explained in more detail below): (A) intracranial motions or ‘cranial kinesis’, (B) lower jaw movements *via* suspensory motions, and (C) coupled movements of the lower and upper jaw.

##### (i) Avian ‘cranial kinesis’: complex movements of the upper beak

Birds show distinct types of intracranial motions, which share the same underlying mechanism in both Palaeognathae (from Ancient Greek *palaiós* ‘old’, and *gnáthos* ‘jaw’) and Neognathae (from Ancient Greek *néos* ‘new’, and *gnáthos* ‘jaw’). The quadrate bones perform a forward rotation (*via* streptostyly), and the jugal bars and pterygoid–palatinum complex transmit this movement to the upper jaw, which induces the elevation of (parts of) the upper beak (Bout & Zweers, 2001). Intracranial motions might also work reversely, producing a downward rotation of the upper beak below the resting position (e.g. in woodpeckers) (Lyons, Baeckens & Van Wassenbergh, 2023).

The three most common types of intracranial motions in birds are prokinesis, rhynchokinesis, and a form of amphikinesis (Zusi, 1984). Note, however, that there is ambiguity in the use of these terms: the description of these types of intracranial motions in birds may differ from how they are used for other vertebrates. Thus, it has been suggested that the adjective ‘avian’ be added to avoid confusion (Bock, 1964; Zusi, 1984). In avian prokinesis, the most common type of intracranial motions in birds, the upper beak rotates around a craniofacial hinge between the upper beak and neurocranium. Avian prokinesis has been described in, e.g. crows (Bock, 1964; Gussekloo *et al*., 2001), finches (Mielke & Van Wassenbergh, 2022), chickens (Van Den Heuvel, 1991), and sparrows (Hoese & Westneat, 1996). Some birds show rhynchokinesis, which involves bending along the dorsal bar of the upper jaw. The bending zone can be narrow or wide and be positioned at different locations along the upper beak (‘proximal’, ‘central’, ‘distal’, ‘double’, and ‘extensive’ rhynchokinesis) (Zusi, 1984). Rhynchokinesis has been described in, e.g. hummingbirds (Zusi, 1984), shorebirds (Estrella & Masero, 2007), and palaeognathous birds (Gussekloo *et al*., 2001; Gussekloo & Bout, 2005). Avian amphikinesis has only been described in rails (Zusi, 1984). It is a combination of rotation around the craniofacial hinge and bending near the tip of the beak, the latter being the result of proximally extended narial openings and flexibility in the dorsal, ventral, and lateral bars (Zusi, 1984). Instead of using amphikinesis as an additional, redundant term for this avian‐specific condition, Bock (1964) suggested using this term for skulls with any combination of two kinetic hinges. However, this broader definition did not prevail in research on bird skull kinematics and today researchers continue mostly to refer to the more specific definition of avian amphikinesis as given by Zusi (1984).

Even though the elevation of the upper beak usually contributes much less to total gape than that of the lower beak, intracranial motions may yield some potential functional advantages for feeding in birds, which remain controversial. Only for avian rhynchokinesis have functional advantages for feeding behaviour been described: in long‐billed probing shorebirds, (distal) rhynchokinesis allows the bird to displace only a small volume of substrate when opening the bill while catching prey (Zweers & Gerritsen, 1996), and in hummingbirds, distal rhynchokinesis enhances feeding efficiency by improving intra‐oral nectar transport (Rico‐Guevara *et al*., 2024). Bock (1964) proposed that the main benefit of avian intracranial motions is an increased closing speed of the beak. This would be particularly important for birds that feed on fast active prey but would also aid in efficient pecking (Van Gennip & Berkhoudt, 1992), seed handling (Mielke & Van Wassenbergh, 2022), and aquatic feeding (Dawson *et al*., 2011). Furthermore, less energy is needed for lower jaw depression while the upper beak is elevated (Nuijens & Bout, 1998; Bout & Zweers, 2001). Other functional advantages of avian intracranial motions that have been discussed are the maintenance of the primary axis of orientation (line of vision) while approaching prey and shock‐absorbing mechanisms (Bock, 1964). However, it has alternatively been proposed that avian intracranial motions might not be an adaptive trait but rather a consequence of increased eye size (Bout & Zweers, 2001) and increased encephalisation (Wilken *et al*., 2025) in birds. Large eyes reduce the bony bars in the lateral aspect of the skull, enabling the transfer of quadrate movement to the upper beak. The enlargement of the braincase was associated with alterations in the spatial arrangement of jaw muscle vectors and reorganisation of the palatal structures, collectively providing the anatomical foundation required for active intracranial motions. As a result, movement of the upper beak became fundamental to the mechanics of bird feeding, playing a crucial role regardless of the specific feeding strategy used.

##### (ii) Avian suspensory motions: complex movements of the lower beak

In addition to intracranial motions, the three‐dimensional movement of the lower beak is a characteristic feature of dimensionally complex feeding behaviours in birds. Suspended from the skull *via* the mobile quadrate bones (streptostyly), the lower jaw lacks a fixed connection to the cranium and can potentially move with multiple DOF. Complex three‐dimensional mandible movements have been described mainly in granivorous songbirds (Ziswiler, 1965; Nuijens & Zweers, 1997; Van Der Meij & Bout, 2006; Mielke & Van Wassenbergh, 2022), but presumably also play a vital role in other birds conducting complex food manipulations, e.g. parrots (Demery, Chappell & Martin, 2011). However, depending on the morphology and the presence or absence of specific ligaments, the DOF of the mandible may be constrained depending on the species. For example, the greenfinch (*Chloris chloris*), which lacks a postorbital ligament that connects the postorbital process on the cranium with the mandible, exhibits significantly more mediolateral mandibular movements during seed husking compared to other finches that possess the postorbital ligament (Nuijens & Zweers, 1997; Van Der Meij & Bout, 2006). In addition to the three‐dimensional movements of the lower jaw as a rigid body, some birds exhibit movements within the mandible itself (intraramal motions). Intraramal motions can occur either *via* bending of the mandible, e.g. in pelicans (Meyers & Myers, 2005), hummingbirds (Yanega & Rubega, 2004), and mallards (*Anas platyrhynchos*) (Dawson *et al*., 2011), or *via* actual intraramal articulations, as seen in nightjars (Bühler, 1970). Regardless of the mechanism, avian intraramal motions enable widening the beak gape, which facilitates obtaining prey items (Bühler, 1970; Yanega & Rubega, 2004; Meyers & Myers, 2005).

In terms of food processing, three‐dimensional movements of the lower beak and intraramal motions can allow for more precise handling of food items of varied sizes, shapes, and hardness.

##### (iii) Coupled ‘cranial kinesis’: linked movements of the upper and lower beak

Since no muscle attaches to the upper jaw that would directly cause upper beak elevation, the only way to raise the upper beak is through force transmission *via* the jugal and pterygoid–palatinum complex (avian cranial kinesis). The source of that force is usually a forward rotation of the quadrate bones. Since lower jaw depression appears to trigger this quadrate rotation and thus upper beak elevation, such coupled intracranial motions are often called ‘coupled cranial kinesis’. Indeed, the primary mandible opener muscle, the m. depressor mandibulae, alone can cause mandible depression and upper jaw elevation simultaneously (Zusi, 1967). However, the specific mechanism of that functional coupling is still not fully resolved. Bock (1964) suggested a coupling mechanism that works *via* the postorbital ligament: the ligament becomes stiff when stretched due to the initial mandible opening, which locks the lower jaw and prevents further depression. Unlocking can only occur *via* a forward rotation of the quadrate bones, which unloads the ligament and causes upper beak elevation. With this mechanism, the mandible could not (entirely) depress without elevating the upper beak. This mechanism, however, could not be confirmed in some species with a postorbital ligament. Chickens (*Gallus gallus domesticus*) and white‐throated sparrows (*Zonotrichia albicollis*), for example, have been shown to move their upper and lower beak partly independently despite possessing a postorbital ligament (Van Den Heuvel, 1991; Hoese & Westneat, 1996). These independent movements might result from the postorbital ligament producing only slight resistance to lower jaw depression (Nuijens & Bout, 1998; Bout & Zweers, 2001). Furthermore, such coupled skull motions have been shown in birds after their postorbital ligament has been cut (Zusi, 1967). Also, birds without a postorbital ligament can still show (partly) coupled beak movement (Mielke & Van Wassenbergh, 2022). Other mechanisms may cause a correlated movement of the upper and lower beak (Bock, 1964; Van Gennip & Berkhoudt, 1992), but they probably differ among species. Given the frequently varying degree of correlation between the movements of the upper and lower beak, coupling is likely rather facultative than obligate in many species (Dawson *et al*., 2011; Mielke and Van Wassenbergh, 2022). Further research is needed to understand better the underlying mechanisms of avian coupled and uncoupled skull motions.

##### (iv) Mandibular processing in Aves

It is generally believed that birds do not chew, possibly because of the common perception that chewing necessitates the presence of teeth or because detailed studies on palaeognathous birds have not found evidence of chewing behaviours (Tomlinson, 2000; Gussekloo & Bout, 2005). Nevertheless, X‐ray analyses seem to indicate the potential presence of cyclic, mandibular processing behaviours in mallard ducks (Anatidae) (Dawson *et al*., 2011; see also https://youtu.be/aoJAyS6iNsk). However, the data recorded in this XROMM study do not permit definitive conclusions on whether the ducks cut or grind the small pellets used in this experiment. Although many bird species lack teeth and possess relatively fragile beaks that likely limit the extent of food breakdown during such movements, mechanical interactions between the beak and food can facilitate the processing of larger prey items commonly consumed, such as gastropods, amphibians, and arthropods.

Moreover, granivorous birds like domestic canaries (Fringillidae) exhibit dimensionally complex beak movements during various stages of seed processing (Mielke & Van Wassenbergh, 2022). These dimensionally complex mandibular motions facilitate seed husking, wherein the mandibles and tongue are utilised to position and orient the seeds for successive bites and manipulations until the husk is removed. Given the functional morphology of the skull, these behaviours qualify as cranio‐suspensory chewing.

Finally, numerous frugivorous birds exhibit diverse mandible–food interactions. Reports indicate that frugivorous birds either grasp entire fruits or engage in ‘piecemeal feeding’, wherein they bite or tear off fruit parts (Moermond & Denslow, 1985). Piecemeal feeding predominantly involves mandibular biting and links ingestion and processing without following a cyclic pattern, thus not meeting the criteria for chewing. Conversely, whole fruit consumers often engage in ‘mandibulation’ – manipulating and processing fruits in their bills by rotating or moving them swiftly with their mandibles (Moermond, 1983; Moermond & Denslow, 1985). While many birds mandibulate fruits primarily to facilitate their transport to the oesophagus (referred to as ‘gulpers’), certain taxa, like tanagers and emberizid finches, mandibulate some food items for over 30 s, resulting in flattening, softening, crushing, or fragmentation, to extract juice from the pulp for ingestion (Moermond, 1983). Birds engaging extensively in such food breakdown processes are termed ‘mashers’ (Moermond & Denslow, 1985; Levey, 1987). Although these behaviours have only rarely been labelled as chewing or ‘mastication’ (Bhattacharyya, 2013), like seed husking in granivorous birds, they align with the chewing definition proposed herein. Considering the functional morphology underlying these mashing mandibulation behaviours, they meet the criteria for cranio‐suspensory chewing.

In summary, birds generally display various forms of skull‐internal motions that often combine into cranio‐suspensory motions, which are sometimes linked to intraramal motions. These intricate movements facilitate complex mandibular mechanics in many avian species and play a crucial role in how the occlusal surfaces of their beaks interact. However, the evolution of the avian crop and the gizzard with its gastric processing has significantly enhanced postoral food processing capabilities, rivalling mammalian mastication. Consequently, birds may have abandoned mandibular processing behaviours such as chewing during their early evolution. Nonetheless, certain dietary preferences and feeding strategies, such as granivory and frugivory, seem to have warranted the inclusion of mandibular processing to supplement gastric milling. Therefore, given their phylogenetic position, some birds seem to have secondarily re‐evolved mandibular processing behaviours, such as bites and cranio‐suspensory chewing.

#### (d) Sauropsids: crocodilians (Crocodylia)

At present, crocodilians are recognised as comprising 28 species distributed among three families: true crocodiles (Crocodylidae), alligators and caimans (Alligatoridae), and gharial and false gharial (Gavialidae). Related to their unprecedented ontogenetic gigantism, in which some adult forms grow to ~15,000 times the weight of their hatchlings (Webb *et al*., 1983; Britton, Whitaker & Whitaker, 2012), many crocodylians undergo drastic ontogenetic dietary shifts. Hatchlings predominantly prey on insects and other small invertebrates (Dodson, 1975; Grenard, 1991). As individuals grow, they progress to consuming fish, crustaceans, amphibians, and small reptiles (Dodson, 1975; Erickson, Lappin & Vliet, 2003; Gignac & Erickson, 2015). In some medium‐sized species, the diet ultimately broadens to include birds and small mammals (Delany & Abercrombie, 1986; Salisbury & Frey, 2000; Erickson *et al*., 2012). Large‐bodied species can feed on mammalian game and armoured prey like turtles (Dodson, 1975; Erickson *et al*., 2003). This ontogenetic dietary diversity suggests diverse feeding behaviours, including processing behaviours to kill and prepare food for digestion.

While chewing or intraoral food‐processing behaviours are rarely discussed as such in crocodilians, diverse biting behaviours and the form and function of the jaw joint have been studied in detail for the spectacled caiman (*Caiman crocodilus*) (Cleuren & De Vree, 1992; Cleuren, De Vree & Schwenk, 2000; Reilly *et al*., 2001). The results of these studies suggest that it applies one or multiple “inertial bites”, “repositioning and killing/crushing bites”, and “transport bites” (Cleuren *et al*., 2000). The number of bites depends on the type and size of the food. Feeding on larger prey usually involves a larger number of bites, with up to 100 repositioning and killing/crushing bites (Cleuren & De Vree, 1992). These bites start with the elevation of the neck and head, followed by rapid upward movement of the hyolingual apparatus to disengage the prey from the teeth. Fast closing thrusts the head forward while retracting the tongue and hyoid, allowing the prey to be caught in a more advantageous position for further processing or swallowing. Considering the functional anatomy of the feeding apparatus with its hinge‐like jaw joint and pronounced cranial elevation (unicranial motions), it seems plausible to assume that these cyclical mandibular killing and crushing bites represent arcuate chewing combined with pronounced unicranial motions (functionally ambignathic).

During chewing, prey may be killed, crushed, punctured, and/or reduced in size. This behaviour can also be interspersed with transport movements to reposition the prey into an area better suited for further processing or may be briefly paused as the prey is grasped between the teeth (Cleuren & De Vree, 1992). Through direct observations and the use of online video resources (Table S1), it seems that chewing‐like behaviours are predominantly exhibited by certain brevirostrine or early developmental stages of longirostrine species when they consume smaller prey items, such as fish, that fit within the oral cavity. By contrast, older more longirostrine individuals appear to limit their processing to a singular or a few transport bites, ceasing once the food is positioned for swallowing. Consequently, it may be posited that with the evolution of slender snouts, facilitating rapid grasping or ‘snap feeding’, longirostrine species have lost the capacity for pronounced chewing behaviours or that their ratchet‐like, ingestive biting with slender pointed teeth does already process the food enough to discard further chewing of small, harmless, and comparatively easily digestible fish prey. On the other hand, more brevirostrine species may engage in chewing when their prey fits within the mouth and requires processing. For prey items that are either too small or too large, most crocodilians seem either to swallow food directly or initially employ ‘simple’ ambiarcuate bites or ingestive bites. However, further research is warranted to elucidate the relationship between snout morphology, prey type, and processing behaviour.

Additionally, some crocodilians may forego chewing when consuming particular prey items, relying instead on using their gizzard for gastric processing as an effective digestive strategy, often facilitated by the extensive use of gastroliths (or gizzard stones) (Corbet, 1960). It has been shown that specimens with gizzard stones have significantly higher gut assimilation efficiencies than those without (Davenport *et al*., 1990). Additionally, crocodiles frequently employ the ‘death‐roll’ technique (Fish *et al*., 2007; Drumheller, Darlington & Vliet, 2019), similar to the rotational feeding observed in caecilians and salamanders. Crocodilian rotational feeding behaviours are well known for killing and dismembering prey to facilitate swallowing and digestion.

In conclusion, certain brevirostrine and subadult longirostrine crocodilians seem to chew some of their food. When cyclic mandibular processing is present, it resembles arcuate chewing, and there appears to be no considerable kinetic potential in the crocodilian cranium. However, crocodilian prey‐handling and processing behaviours are more complex than described here, and the occurrence of chewing may depend on the species, the type of prey, and the ontogenetic stage of the individual crocodile. Based on our observations and a limited number of studies, it appears that the ancestral behaviour in crocodiles may have included a functionally ambignathic form of arcuate chewing. At the same time, both gastric processing, involving gizzards, and semi‐alimentary processing, such as rotational feeding, might have largely substituted for oropharyngeal processing in certain crocodilians.

#### (e) Synapsids: mammals (Mammalia)

The mammalian skull is characterised by the absence of intracranial and suspensory motions, leading to limited internal skull movements primarily centred around mandibular motions *via* the jaw joint apart from the auditory ossicles. The simplification from multiple skull‐internal motions to a single form in mammals might initially suggest diminished mandibular or occlusal mobility and a trend toward simpler chewing behaviours. Yet, this assumption is misleading, as mammals demonstrate remarkably complex and intricate chewing mechanics.

The enduring perception of mammals as the sole ‘chewers’, or the only chewers to display dimensionally complex chewing behaviours, can likely be attributed to several key factors, including: (*i*) the conspicuous complexity of mammalian chewing behaviours, which are often readily observable; (*ii*) the accessibility of mammalian specimens, given that many mammals inhabit nearby terrestrial environments, facilitating easier observation and study compared to non‐mammals; (*iii*) the tendency for many mammals to possess relatively larger body sizes than non‐mammals, which can enhance their visibility and facilitate data collection; (*iv*) the familiarity of mammalian behaviours, as many of their observable actions are relatable to human experiences, thus fostering greater engagement and understanding among researchers and observers alike. Rectifying this oversimplified and evolutionarily inaccurate perspective is one of the primary objectives of this review. The subsequent discussion delves into the diverse functional morphology of mammals and sheds light on the evolution of mammalian chewing behaviours.

Among various distinguishing traits, mammals are characterised by the development and diversification of specialised teeth capable of precise occlusion, along with well‐developed and distinct masticatory muscles and muscular tongues. These features, alongside their unique evolutionary jaw joint, seem to facilitate the nuanced consideration of the diverse and intricate mammalian chewing behaviours, often referred to as mastication. Mastication stands as the predominant food‐processing behaviour for almost all mammals. Nevertheless, this terminological homogeneity does not accurately represent the complexity and diversity of masticatory movements in mammals. Mammals exhibit a craniomandibular diversity that has influenced the evolution of different approaches to food processing in relation to diet. In the first classifications, mammals were typically grouped into three masticatory types. These categories highlight striking adaptations of the masticatory apparatus identified by their discernible specialisations in the skull, teeth, and mandible morphology, resulting in distinctive masticatory movements. The initial three groups recognised correspond to the carnivores, herbivores (ungulates), and rodents (von Teutleben, 1873; Lubosch, 1907; Turnbull, 1970).

##### (i) Carnivoran mammals

Carnivores typically exhibit a relatively hinge‐like jaw joint that is dorsoventrally aligned with the tooth row, a large temporalis relative to the other masticatory muscles, and carnassial teeth with a large shearing surface focused on a small area. These features emphasise predominantly arcuate dorsoventral motions during the masticatory cycle and facilitate a powerful bite force at the molars and large gape (Smith & Savage, 1959; Crompton & Hiiemae, 1969). Studies of cineradiography synchronised with EMG data performed on the cat *Felis catus* exemplify the clade Carnivora. In addition to typical carnivoran masticatory movements, this species displays slight anteroposterior and transverse components of the masticatory cycle along with unilateral chewing (Gorniak & Gans, 1980), suggesting the presence of a moveable interramal joint and more than hinge‐like jaw joint motions. Based on its masticatory morphology, it is hypothesised that those movements also happen in the raccoon (*Procyon lotor*) (Gorniak, 1986). The jaw adduction motor pattern of carnivores, distinguished by early symmetric activation of the zygomaticomandibularis (or vertical parts of the deep masseter) and synchronous activation of triplets I (working‐side temporalis and balancing‐side superficial masseter and medial pterygoid) and II (balancing‐side temporalis and working‐side superficial masseter and medial pterygoid), has been primarily observed in the cat (Weijs, 1994). Note that the working side is where the chewing occurs, while the balancing side is the opposite side, which helps stabilise and balance the jaw to maintain alignment and ensure smooth, coordinated movements. The ‘carnivore symmetric pattern’ is also observed in the ferret (*Mustela putorius furo*) and kinkajou (*Potos flavus*) (Davis & Williams, 2017), potentially representing a functional adaptation for concentrating vertical jaw movements essential for vertical shearing specialisation. However, the late activity of the balancing side of the zygomaticomandibularis muscle in the kinkajou introduces some degree of variability associated with the morphology of the jaw system and the function of masticatory motor patterns. This variation may be linked to the production of transverse movements during mastication, essential for grinding plant foods (Davis & Williams, 2017). Therefore, carnivorous mammals appear to employ a mastication pattern largely resembling arcuate chewing. At the same time, certain species display greater dimensional complexity, allowing for longitudinal and transverse mandibular excursions that are potentially linked to dietary adaptations.

##### (ii) Herbivorous mammals

Herbivores generally show a free‐sliding jaw joint positioned above the level of the tooth row, with the masseter and pterygoid muscles being relatively larger than the temporalis. Their square and ridged teeth are optimised for grinding food. These features emphasise predominantly mediolateral motions during the masticatory cycle and facilitate bite force distribution along the tooth row at low gape (Smith & Savage, 1959; Crompton & Hiiemae, 1969). Herbivores are typified by the clades Artiodactyla and Perissodactyla, and experiments using cineradiography and EMG were performed with goats (*Capra hircus*) (De Vree & Gans, 1976) and pigs (*Sus scrofa*) (Herring & Scapino, 1973; Montuelle *et al*., 2018; Olson *et al*., 2021), showing that they chew strictly unilaterally for the former and both unilaterally and with alternative bilateral chewing for the latter. The jaw‐closing motor pattern in herbivores is characterised by an asynchrony of triplets I and II (and sometimes between the working and balancing sides of the deep masseter), as well as a delayed activation of the working side of the lateral pterygoid (Weijs, 1994; Williams *et al*., 2007). This ‘transverse pattern’ has been identified in the goat, the alpaca (*Lama pacos*), and the horse (*Equus ferus*), where triplet I acts as a fast closer of the mandible, and triplet II may be activated to contribute to the power stroke. There are marked differences in the triplet pattern of the horse compared to other large herbivores (Williams *et al*., 2007; Williams, 2019). In non‐ungulate herbivores, such as the rock hyrax (*Procavia capensis*) (Janis, 1979), masticatory movements resemble those observed in other ungulate herbivores. Finally, the masticatory motor patterns of herbivorous marsupials are highly diversified and distinct: the koala (*Phascolarctos cinereus*) shares a pattern similar to the alpaca (Crompton *et al*., 2008; Crompton, Owerkowicz & Skinner, 2010), while the masticatory motor pattern of the southern hairy‐nosed wombat (*Lasiorhinus latifrons*) (Crompton, 2011) and the red kangaroo (*Osphranter rufus*) as well as the tammar wallaby (*Notamacropus eugenii*) (Crompton *et al*., 2008) are highly derived among mammals, suggesting that these herbivorous mammalian patterns can be further refined (Vinyard *et al*., 2011) or may require splitting. Therefore, it appears that herbivorous mammals employ a mastication pattern mainly relying on mediolateral (transverse chewing), while certain species evolved derived chewing mechanics.

##### (iii) Rodent mammals

Rodents are usually characterised by a gliding‐saddle or ‘gutter‐shaped’ jaw joint facilitating antero‐posterior movements. The joint is positioned above the level of the tooth row, and the relative size and complexity of the masseter muscle are greater than those of the rest of the jaw muscles. The dentition is also peculiar, with the presence of a single pair of well‐developed incisors completely separated from the cheek teeth by a pronounced diastema. These features allow the jaw of rodents to operate in two separate ways *via* anterior shift of the jaw for gnawing at the incisors and posterior shift to ensure masticatory functions at the cheek teeth (Turnbull, 1970). Rodents represent the most speciose mammalian clade, exhibiting remarkable diversity in their masticatory apparatus. Three primary jaw morphotypes – sciuromorph, hystricomorph, and myomorph – have long been classified (Brandt, 1855), typified respectively by the Eastern grey squirrel (*Sciurus carolinensis*), domesticated guinea pig (*Cavia porcellus*), and brown rat (*Rattus norvegicus*) (Cox & Jeffery, 2011). Functionally, these distinctions correspond to specialisations for gnawing (squirrel), chewing (guinea pig), and generalist feeding (rat) (Cox *et al*., 2012). Rodent mastication is marked primarily by anteroposterior and modestly transverse jaw movements (Crompton & Hiiemae, 1969). Additionally, many rodents seem to exhibit either alternate bilateral chewing, in which food is processed on one side of the mouth at a time, while the jaws occlude simultaneously, or ‘true’ simultaneous bilateral chewing as observed in the rat (Hiiemäe & Ardran, 1968), Desmarest's hutia (*Capromys pilorides*), and the Hispaniolan hutia (*Plagiodontia aedium*) (Woods, 1976). Notably, the South African springhare (*Pedetes capensis*) stands out as the only rodent reported to solely employ simultaneous bilateral chewing (Offermans & De Vree, 1990). The guinea pig (Byrd, 1981), woodchuck (*Marmota monax*) (Druzinsky, 1995), alpine marmot (*Marmota marmota*), and mountain beaver (*Aplodontia rufa*) (Druzinsky, 1995; Stefen, Ibe & Fischer, 2011) are examples of rodents exhibiting intricate masticatory behaviours combining both alternative bilateral and some unilateral chewing. Finally, some rodents predominantly engage in unilateral chewing during mastication, as is the case in the nutria or coypu (*Myocastor coypus*) (Woods, 1976) and the mountain beaver (Druzinsky, 1995). The latter uses a modified approach by engaging in several unilateral chewing cycles before switching sides and repeating this behaviour until the completion of mastication. Two types of masticatory motor patterns are recognised in rodents: the ‘rodent symmetric’ and ‘rodent alternate’ patterns (Weijs, 1994). The former corresponds to the symmetric activity of both sides of each muscle, typical of the springhare (Offermans & de Vree, 1993) and the rat (Weijs & Dantuma, 1975). It initiates with the activation of the temporalis, followed by synchronous activation of the masseter and medial pterygoid muscles, and concludes with the bilateral activation of the lateral pterygoids. This activation pattern enhances bilateral antero‐posterior (also referred to as longitudinal, proal, or propalinal) translation movements. The latter entails a significant reduction of triplet I, enabling the sole activity of triplet II, where the temporalis fires first, followed by the masseter and pterygoid muscles. This pattern is observed in the golden hamster (*Messocricetus auratus*) (Gorniak, 1977), the guinea pig (Byrd, 1981), the alpine marmot, and the beaver (Druzinsky, 1995) and results in the muscles alternating between the working and balancing side during the power stroke of the masticatory cycle. Due to the extensive diversity in rodent masticatory morphology, the potential for discovering additional motor patterns exists, albeit limited, as not all anatomical differences necessarily translate into functional distinctions (Druzinsky, 1995) and placental mammals generally demonstrate conservation of motor patterns (Vinyard *et al*., 2011). Consequently, rodents employ a mastication pattern mainly featuring longitudinal mandible excursions.

##### (iv) Lagomorph mammals

Lagomorphs are the sole known mammals featuring an intracranial joint (Bramble, 1989; Wood‐Bailey, Cox & Sharp, 2022). Their masticatory apparatus represents an intermediate between herbivores (ungulates) and rodents. Similar to ungulates, the condyle is positioned well above the tooth row, the masseter muscle is simpler than in rodents and dominated by the superficial masseter, and the molars feature deep transverse ridges. Like rodents, they possess a gliding‐saddle jaw joint and well‐developed incisors that come together *via* an antero‐posterior shift of the mandible (Smith & Savage, 1959; Weijs & Dantuma, 1980). Cineradiographic data on the rabbit (*Oryctolagus cuniculus*) reveal a masticatory pattern characterised by unilateral chewing with a significant transverse component, resembling ungulates. Additionally, rabbits exhibit gnawing behaviour typical of rodents involving antero‐posterior and vertical cyclic movements of the jaw (Ardran, Kemp & Ride, 1958; Weijs & Dantuma, 1980; Schwartz *et al*., 1989). Moreover, the intracranial joint may play a role in facilitating intracranial movements during feeding, although this possibility remains largely unexplored, given that the joint is more commonly linked to the animals' saltatory locomotion. EMG data suggests that the lagomorph masticatory motor pattern is similar to the ‘transverse pattern’ of ungulate herbivores (Weijs & Dantuma, 1980; Weijs, 1994). Consequently, lagomorphs employ a peculiar form of mastication in which they feature significant transverse as well as longitudinal mandible excursions, which may be influenced by intracranial motions.

##### (v) Ancestral and generalist mammals

An additional category, which corresponds to generalised forms of masticatory architecture and movements in mammals, is typically associated with the ancestral condition of the masticatory musculature present in early mammals (Crompton, 1963; Crompton & Hiiemae, 1969; Turnbull, 1970). Generalists possess a relatively unspecialised masticatory morphology compared to the above groups. Within this group, the jaw joint is positioned above the level of the tooth row, and the jaw musculature is characterised by the dominance of the temporalis and deep masseter muscles, which serve as the primary sources of bite force generation. While contributing to bite force, the superficial masseter and pterygoid muscles simultaneously generate transverse movements (Crompton & Hiiemae, 1969; Turnbull, 1970). The molars have prominent and sharp cusps to puncture and shear food such as insects or fruits and are usually (but not always) tribosphenic. Jaw movements involve a degree of mandibular rotation along the longitudinal axis (long‐axis rotation) during vertical and transverse motions for puncturing and shearing, respectively (Oron & Crompton, 1985). Ancestral mammalian jaw movements are typified by cineradiographic studies of feeding American opossums (*Didelphis marsupialis*) (Crompton & Hiiemae, 1970; Hiiemae & Crompton, 1971), which show unilateral shearing chewing as well as hemimandibular rotation along the longitudinal and vertical axes as also documented in the gray short‐tailed opossum (*Monodelphis domestica*) (Bhullar *et al*., 2019) and the Virginia opossum (*Didelphis virginiana*) (Stilson *et al*., 2023). Additional mediolateral and anteroposterior translations along the jaw articulation have been demonstrated, particularly for the latter.

Other clades such as Chiroptera, Afrosoricida, and Primates are associated with generalist forms. For example, the little brown bat (*Myotis lucifugus*) (Kallen & Gans, 1972) and possibly the Indian flying fox (*Pteropus giganteus*) (Greet & De Vree, 1984) exhibit unilateral chewing as well as vertical and longitudinal mandibular rotation. Unilateral chewing and long‐axis rotation but not vertical rotation (yaw) have been observed in the tenrec (*Tenrec ecaudatus*) (Oron & Crompton, 1985), the common tree shrew (*Tupaia glis*), and the brown greater galago (*Otolemur crassicaudatus*) (Hiiemae & Kay, 1973). Finally, neither vertical nor longitudinal mandibular rotation (long‐axis rotation) are found in the crab‐eating macaque (*Macaca fascicularis*), the olive baboon (*Papio anubis*), the chimpanzee (*Pan troglodytes*), the squirrel monkey (*Saimiri* sp.), or the spider monkey (*Ateles* sp.) as their interramal joint is fused (Hiiemae & Kay, 1973; Luschei & Goodwin, 1974; Hylander, Johnson & Crompton, 1987; Wall, 1999) but their movements remain highly similar to those of other generalists (Hiiemae & Kay, 1973; Hiiemae, 1978; Hylander & Crompton, 1986). The jaw‐closing motor pattern of the generalists is characterised by an initial symmetrical activation of both sides of the zygomaticomandibularis followed by an asynchronous activation of triplet I, then triplet II (Weijs, 1994). This ‘ancestral pattern’ is evidenced by data on the tenrec (Oron & Crompton, 1985), the American opossum (Hylander & Crompton, 1986), the little brown bat (Kallen & Gans, 1972), and the Indian flying fox (De Gueldre & De Vree, 1988, 1989). The pattern is modified in the Belanger's treeshrew (*Tupaia belangeri*) (Vinyard *et al*., 2005), as well as in strepsirrhine primates such as the brown greater galago, the ring‐tailed lemur (*Lemur catta*), and Verreaux's sifaka (*Propithecus verreauxi*) (Hylander *et al*., 2011). Additionally, various anthropoid primates (Vinyard *et al*., 2005, 2008, 2019) also deviate from this pattern as the synchronous activation of both sides of the temporalis muscle does not occur and significant interspecific variation in motor patterns is observed (Vinyard *et al*., 2008).

Consequently, generalist mammals often employ a mastication pattern featuring arcuate open–close movements of the whole mandible combined with vertical‐ and longitudinal‐axis rotation of the individual hemimandibles. Recent detailed studies suggest that the ancestral condition among mammals may have included five DOF along the jaw joint: pitch, yaw, roll, surge, and sway (Bhullar *et al*., 2019; Stilson *et al*., 2023). This indicates that, in addition to the commonly observed generalist pattern, mammals may have ancestrally utilised hemimandibular translations along the longitudinal and transverse axes, resulting in antero‐posterior and mediolateral movements, respectively. This pattern, however, was repeatedly simplified with the rise of fused mandibles, which hinder more complex movements of the individual hemimandibles.

Interestingly, certain mammals forego mastication entirely because their primary diet does not necessitate mechanical breakdown. Baleen whales (Mysticetes), for instance, are filter feeders that extract small prey such as krill and small fish from the water. Likewise, myrmecophagous mammals that consume minuscule prey like termites and ants have either developed alternative food‐processing strategies or eliminated oropharyngeal processing altogether.

##### (vi) Myrmecophagous mammals

Termite and ant‐eating (myrmecophagous) mammals exhibit distinctive features such as flat and elongated jaws. The jaw joint is flattened and positioned at the level of the dorsal edge of the dentary. In this group, teeth are either extremely reduced or completely absent, accompanied by a considerable reduction in jaw musculature. These characteristics seem to suggest that mastication does not occur in myrmecophagous mammals, even if some degree of mandibular movement, such as slight anteroposterior and transverse movements and rotation along the longitudinal axis of the jaw, is possible during mouth opening (Naples, 1999; Ferreira‐Cardoso *et al*., 2020). While there are no cineradiographic or EMG data on feeding in this group, direct observations of the short‐beaked echidna (*Tachyglossus aculeatus*) suggest that they engage in tongue–palate interactions in which they use spiny pads on their tongue to grind food against keratinous palatal teeth (Griffiths, 1965, 1968). The absence of teeth and the presence of whole prey in the stomach content of the lesser anteater (*Tamandua tetradactyla*) indicate the absence of mastication (Lubin, Montgomery & Young, 1977; Montgomery, 1985). This assumption likely extends to edentulous pangolins such as the Chinese pangolin (*Manis pentadactyla*) and the Malayan pangolin (*Manis javanica*), which are thought to employ a combination of gizzard‐like gastric processing (Krause & Leeson, 1974) and gastric digestion of chitin through chitinase activity (Cheng *et al*., 2023) to facilitate digestion, respectively. Myrecophagous armadillos (Dasypodidae), the aardvark (*Orycteropus afer*), the aardwolf (*Proteles cristatus*), and the numbat (*Myrmecobius fasciatus*), retain teeth (Reiss, 2000) and, thus, may only chew occasionally when consuming larger food, as seen in armadillos (Smith & Redford, 1990). It is highly probable that myrmecophagous mammals depend predominantly on a combination of intra‐alimentary digestion, facilitated by oral and gastric secretions and enhanced by intestinal microbiota (Teullet *et al*., 2023) and gastric processing if gizzard‐like structures are present, for food processing.

In summary, mammals are frequently considered the archetypal – and often exclusive – ‘chewers’, with mastication widely regarded as a uniquely mammalian trait. Yet, as previously emphasised, chewing and mastication are distinct concepts that should not be conflated. Although mastication probably represents the ancestral or even synapomorphic food‐processing strategy in mammals, a number of species forgo oropharyngeal processing altogether, and instead have evolved alternative intraoral mechanisms such as tongue–palate interactions or an almost exclusive dependence on gastric processing or chemical digestion, as exemplified by toothless myrmecophagous and microphagous taxa. Furthermore, a subset of mammalian chewing behaviours does not align with the broadly accepted criteria of mastication – typically defined as involving precise occlusion, saliva‐mediated bolus formation, and jaw movements more complex than simple arcuate motions – nor with the novel definition advanced herein, which confines mastication to dimensionally complex chewing *via* the TMJ. As detailed earlier, these criteria are neither universally expressed nor predominant among mammals. For instance, many carnivores chiefly utilise arcuate jaw motions to shear food into manageable‐sized fragments.

The study of mammalian masticatory systems historically emphasised major feeding types, given their crucial role in the evolutionary radiation of mammals. The early establishment of masticatory groups relied on direct observations and inferred movement reconstructions derived from craniomandibular and dental anatomy. Subsequently, these groups underwent refinement with experimental data from *in vivo* studies of masticatory movements. This process established the basis for understanding masticatory movements in major mammalian groups but also contributed to the over‐generalisation of masticatory processes of these groups, primarily due to the limited number of species studied (Gans, Vree & Gorniak, 1978). Interestingly, the diversity in masticatory musculature suggests that many more ‘masticatory groups’ may exist (Ercoli *et al*., 2023), echoing the complexity of masticatory movements and motor patterns briefly reviewed here. Consequently, there appears to be no universal mammalian masticatory pattern. Instead, it might be beneficial to use terms such as carnivore, herbivore, rodent, lagomorph, and ancestral or generalist type before ‘mastication’ to specify the specific pattern of mandibular motions being discussed.

## VII. EVOLUTION OF VERTEBRATE FOOD PROCESSING

**Fig. 8:**
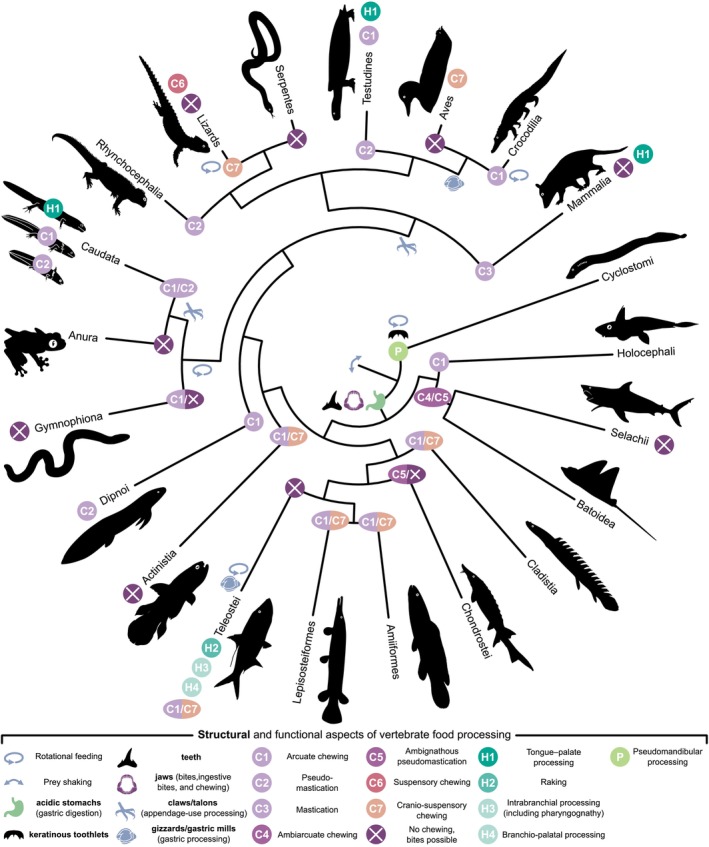
Phylogeny of vertebrate food processing. Commonly accepted evolutionary tree of vertebrates, with turtles positioned as the sister group to archosaurs, collectively referred to as “Archelosauria” (Iwabe *et al*., 2005; Crawford *et al*., 2012, 2015; Field *et al*., 2014). Cyclostomes lack both jaws and teeth for chewing but possess keratinous toothlets (Huysseune & Sire, 1998; Smith & Coates, 1998) for pseudomandibular processing, a form of oropharyngeal food processing. Real teeth and jaws evolved in gnathostomes (Donoghue & Rücklin, 2016), and with that, chewing, bites, and ingestive bites. Highly acidic stomachs aiding chemical digestion also seem to have evolved in gnathostomes (Koelz, 1992; Stumpp *et al*., 2015). Furthermore, outside of amniotes, claw‐shaped terminal phalanges seem to have evolved in the ancestors of both frogs and salamanders, known collectively as batrachians (Liem, 1970; Blotto *et al*., 2020). This figure provides a synthesis of the most likely ancestral feeding behaviours for each vertebrate group, as derived from comparative considerations of functional morphology and oropharyngeal food processing presented in Sections IV., VI.. While the current scarcity of data precludes the application of robust statistical approaches, such as ancestral state reconstructions, our detailed comparative considerations of the functional morphology of key taxa enable reconstructing the major transitions during the evolution of vertebrate food‐processing behaviours. The most likely ancestral state is illustrated at the corners of the lines near each branching point, whereas the processing behaviours indicated next to each group's name or symbol represent the range of known behavioural diversity diverging from the presumed ancestral condition. When a slash separates two feeding behaviours, this denotes uncertainty about the (ancestral) feeding mode and highlights the need for additional research.

In this section, we examine key evolutionary transitions, both firmly supported and hypothetical, arising from the integration of our preceding comparative review of vertebrate food processing with established phylogenetic frameworks. Figure 8 presents a synthesis of the principal findings. While some aspects remain speculative and inconclusive, they offer a vital foundation for targeted, in‐depth research seeking to address these unresolved questions.

Most non‐teleost vertebrates process food using their mandibles. These behaviours may be non‐cyclic and non‐rhythmic, resembling simple bites that help kill, crush, slice, or puncture food, as seen in many snakes or certain sharks. Alternatively, bites may occur in a continuous, rhythmic manner, resembling ingestive bites or chewing. Ingestive bites are often employed by species that generally utilise oropharyngeal food processing, particularly when handling food that exceeds the capacity of their oral cavity (e.g. common during crocodile and salamander feeding) or as the sole form of processing in species lacking tongues or tongue‐replacement strategies for precise oropharyngeal food transport and orientation (e.g. certain snakes).

Cyclic oropharyngeal food processing such as chewing can generally be considered to require tongues capable of precise food transport. Tongues may either be of the functional ‘hydrodynamic type’, as seen in many aquatic vertebrates that use induced water flows *via* hyobranchial‐ or (hyo‐)lingual motions to transport food intraorally (Liem, 1991; Michel *et al*., 2015), or the functional–anatomical ‘terrestrial type’ of various tetrapods, which is primarily used to make direct contact with the food to reposition it intraorally. It is important to note that in certain species, prominent fleshy tongues may serve as functional–anatomical tongues for terrestrial feeding, yet can be repurposed for indirect hydrodynamic transport during aquatic feeding. Regardless of the type, tongues offer the ability to position food between the occlusal surfaces of the jaws and other hard structures within the mouth. This mechanical interaction reduces the risk of food falling out or prey escaping and is considered a direct facilitator of repeated and pronounced mandibular processing (chewing).

The gnathostome outgroup behaviour, exemplified by cyclostomes, appears to functionally resemble chewing. However, in the absence of jaws, this behaviour does not constitute true chewing but pseudomandibular processing, which may comprise pseudobites and pseudochewing.

Chewing, defined as cyclic and rhythmic mandibular processing, likely evolved with the emergence of jaws in gnathostomes as previously implied (Richard *et al*., 2023). Chimeras, with their modified skulls and autostylic jaw suspension, appear to exhibit a form of arcuate chewing, whereas elasmobranchs (sharks and rays), possessing a feeding apparatus more akin to extinct, early‐diverging chondrichthyans (with hyostylic jaw suspension), use ambignathic chewing. Owing to limited evidence, it remains uncertain whether chondrichthyans ancestrally relied on dimensionally complex (ambignathic pseudomastication) or simple open–close movements (ambiarcuate chewing). However, considering that jaws are thought to have arisen from relatively flexible and mobile pharyngeal arches, it appears likely that ambignathic pseudomastication may constitute the ancestral condition among gnathostomes.

In osteichthyans, the jaws were ancestrally fixed to the cranium through autostylic jaw suspension, thus likely limiting ambignathic chewing. In early actinopterygians, which became amphistylic, arcuate or cranio‐suspensory chewing may have developed. More research is necessary to verify the presence of suspensory or intracranial movements during chewing in extant early‐diverging actinopterygians. Interestingly, similar to elasmobranchs, some ray‐finned fish, particularly chondrosteans, have re‐evolved hyostylic jaw suspensions and may be capable of ambiarcuate chewing. However, detailed observations and functional analyses of their feeding behaviours, including food processing, remain scarce. Indeed, the benthic and planktivorous feeding habits of sturgeons and paddlefish, respectively, may indicate that chondrosteans have completely abandoned oropharyngeal food processing.

Teleosts have uniquely developed anatomical adaptations to enable food processing across three processing centres. The emergence of these diverse processing strategies seems linked to the increasing complexity of their highly kinetic skulls (intracranial motions) and ultimately the innovation of jaw protrusion, enhancing their capacity for powerful suction feeding. The evolution of often relatively delicate, protrusible jaws, *via* intracranial and suspensory motions, involved a mechanical trade‐off with the ability to exert strong bites. Consequently, the ancestral teleost feeding behaviour likely included either predatory microphagous particulate feeding (*via* suction feeding) or filter feeding, lacking forms of oropharyngeal food processing.

Following the initial loss of mandible‐based processing due to their initial microphagous or filter feeding strategy, teleosts likely encountered opportunities to prey upon larger and more challenging‐to‐digest food items. However, exploiting such prey often requires mechanical processing prior to swallowing. This evolutionary constraint likely spurred the development of new oropharyngeal structures and, ultimately, the emergence of novel, specialised food‐processing strategies within this group. These include hyoideal processing (raking), characterised by hyoid arch interaction with the palate; intrabranchial processing, involving interactions between branchial arch elements; and branchio‐palatal processing, in which the branchial arch operates against the palate. Regardless, some teleost groups (e.g. pufferfish, triggerfish, parrotfish, trahiras) have secondarily lost or reduced jaw protrusion mechanisms and re‐adapted mandibular processing strategies. Conversely, certain early‐diverging teleost lineages have retained digestive strategies devoid of oropharyngeal processing. The strategy of relying exclusively on chemical digestion has independently re‐evolved in several species that consume small, easily digestible prey requiring no oropharyngeal processing. Additionally, species that feed on tough, nutrient‐poor diets lacking teeth have developed gastric milling capabilities using gizzards (Arnette *et al*., 2024).

The other group of osteichthyans, the sarcopterygians, underwent a marked reduction in skull complexity, leading to decreased intracranial mobility and reinforced cranial structures. While the nature of oropharyngeal food processing in coelacanths remains poorly understood, it is plausible that they engage in either arcuate chewing or cranio‐suspensory chewing facilitated by their moveable suspension and intracranial joint. Lungfish, another early‐branching sarcopterygian clade, have evolved predominantly immobile skulls and are generally believed to have ancestrally employed arcuate chewing. Notably, the Australian lungfish (*Neoceratodus forsteri*) demonstrates dimensionally complex, mandible‐based chewing (pseudomastication), potentially representing the earliest known instance of autostylic pseudomastication in vertebrates. Given that *Neoceratodus* is often regarded closely to reflect the anatomical condition of early‐diverging lungfish, one might argue that pseudomastication constitutes the ancestral processing behaviour within this group. However, osteological evidence from the jaw joint appears to support arcuate chewing as their ancestral processing behaviour (Miles, 1977; Zhu & Yu, 2002).

Among extant tetrapods, lissamphibians (frogs, salamanders, and caecilians) are particularly interesting because they may offer insights into the early evolution of tetrapods during the transition from aquatic to terrestrial life (Schwarz *et al*., 2023). Caecilians present intriguing and challenging study cases due to their status as the sister taxon to frogs and salamanders and their specialised anatomy for a burrowing (fossorial) lifestyle. This lifestyle complicates the study of their feeding behaviour. However, limited research suggests that caecilians may have initially used arcuate chewing or employed powerful rotational feeding to compensate for the absence of oropharyngeal food processing. Frogs appear to have ancestrally discarded oropharyngeal food processing, which aligns with their dietary habit of gulping small invertebrate prey using their large mouths. Yet, further research is needed to explore potential chewing behaviours in predatory tadpoles and the early evolution of frog feeding mechanisms. Salamanders appear unique among lissamphibians in that they regularly exhibit oropharyngeal food processing. Three primary forms of food processing, seemingly linked to specific ontogenetic morphotypes exist. Considering the distinctive feeding behaviours, developmental morphotypes, and phylogenetic placement of studied salamanders, it seems plausible that salamanders ancestrally employed either arcuate chewing or pseudomastication (functionally masticatory behaviours).

Amniotes, which include sauropsids and synapsid mammals, present a compelling case study due to the longstanding view that chewing and mastication are exclusive to mammals. However, our considerations reveal that functionally masticatory behaviours including true mammalian mastication – characterised as dimensionally complex mandibular food processing *via* the jaw joint – seem to have evolved independently at least three times among amniotes, specifically among rhynchocephalians, turtles, and mammals. Intriguingly, functional masticatory behaviours might as well be argued to have been ancestral to amniotes, possibly arising early within stem‐amniotes. However, thorough palaeontological investigations are needed to determine whether jaw joints and teeth capable of dimensionally complex chewing were present in stem representatives of turtles, rhynchocephalians, and their ancestors dating back to the synapsid–sauropsid split, or potentially beyond. Beyond conjecture, compelling evidence indicates that the tuatara, the sole living representative of the early‐diverging rhynchocephalian lineage, exhibits pseudomastication that may rival the general dimensional complexity of true mammalian mastication.

Lepidosaurs, comprising snakes and lizards, have re‐evolved pronounced kinetic potential in their skulls, allowing intracranial motions that markedly increase cranial flexibility. This morphological adaptation enables some species, particularly snakes, to consume prey exceeding their own head size. Consequently, their food‐processing repertoire has undergone substantial modification. Several snake species have evolved alternative feeding strategies, including discrete crushing or slicing bites. Cyclic mandibular processing behaviours, such as the distinctive ‘pterygoid walk’ or mandibular sawing, facilitate a dual role (food ingestion and processing) and thus, resemble ingestive biting mechanisms. Notably, snakes seem to lack pure cyclic oropharyngeal processing, potentially due to the loss of tongues capable of repositioning food within the oral cavity between chewing cycles or because their prey frequently exceeds the size constraints that would enable alternative oropharyngeal food transport mechanisms. Lizards, on the other hand, possess a more ‘classical’ tongue anatomy that facilitates intraoral food transport, and many species use chewing behaviours. The ancestral condition among lizards likely involved various ‘kinetic’ skull‐internal motions between elements of the cranial and suspensory parts, thus potentially constituting cranio‐suspensory chewing. However, due to changes in cranial anatomy, some species have lost certain types of skull‐internal motions. As a result, some lineages have evolved to rely solely on suspensory chewing or have completely abolished oropharyngeal processing.

Turtles represent the second group of amniotes that exhibit functional masticatory behaviours. Various herbivorous turtle taxa employ dimensionally complex chewing that combines arcuate mandible movements with longitudinal retraction and protraction, resulting in a dimensionally complex cropping or grinding behaviour. Lineages that transitioned to different diets seem to have significantly reduced or lost their ability to perform longitudinal mandibular movements. While the combination of herbivory and pseudomastication appears to have been ancestral to turtles, side‐necked turtles seem to have initially shifted to a more carnivorous diet and consequently lost their gliding jaw joint and ability to use pseudomastication. However, among the predominantly arcuate‐chewing side‐necked turtles, at least one species has convergently evolved an alternative anatomical solution to permitting longitudinal mandibular movements and thus enabling pseudomastication.

Archosaurs, encompassing birds and crocodiles, have evolved a remarkable digestive adaptation, the gizzard, which acts as a ‘chewing stomach’ that largely shifts mechanical processing from the oropharyngeal to the gastric phase. Birds seemingly completed this evolutionary shift early on, leading most species not to rely on oropharyngeal food processing. However, some bird species have secondarily reintroduced mandibular processing behaviours, such as biting and chewing. Given their peculiar kinetic skulls, these birds employ cranio‐suspensory chewing. While crocodiles are generally regarded as non‐chewers, some extant, more brevirostrine taxa, as well as certain longirostrine taxa during early developmental stages, appear to engage in functionally ambignathic arcuate chewing. Regardless, in most modern crocodiles, gastric processing involving gizzards, alongside semi‐alimentary behaviours like rotational feeding (‘death roll’), seems to have largely replaced oropharyngeal processing. Further investigation is necessary to clarify how variations in cranial and rostral morphology (brevirostrine/longirostrine), diet, and ontogenetic stage affect the occurrence and diversity of chewing behaviours across crocodilians.

Mammals, often regarded as the quintessential or archetypal chewers, constitute the third amniote group to demonstrate functional masticatory behaviour. Although definitions of mastication can vary considerably, most specialists agree that true mastication is a mammalian trait. However, mammals display remarkable diversity not only in their masticatory mechanics but also in a range of alternative oropharyngeal food‐processing behaviours. While mastication appears to have been the ancestral mode of mammalian food processing, some species have entirely relinquished mandibular processing, evolving alternative intraoral strategies such as tongue–palate interaction or relying chiefly on chemical digestion – a pattern particularly evident in myrmecophagous and microphagous taxa. Moreover, a number of mammalian chewing behaviours that previously surpassed conventional definitions of mastication even fail adequately to meet the revised criteria proposed here, which limit mastication to dimensionally complex jaw movements mediated by the TMJ. In fact, functional divergence within mammalian mastication can be at least as great as that observed between mammalian mastication and pseudomastication in non‐mammalian vertebrates. For instance, while some (hyper)carnivorous mammals are largely restricted to simple arcuate jaw movement, animals like the tuatara and sirenid salamanders exhibit highly complex mandibular dynamics during pseudomastication paralleling the more complex ancestral behaviour of mammalian mastication.

## VIII. CONCLUSIONS

(1) Our findings suggest that chewing, defined as cyclic oropharyngeal food processing using mandibular arch‐derived jaws, emerged with the rise of jaws in gnathostomes and provide evidence for dimensionally complex chewing involving movements of both the upper and lower jaws (i.e. ambignathic pseudomastication) being the ancestral condition. This hypothesis aligns with the probable relatively flexible nature of the earliest gnathostome jaws, derived from the mandibular arch (first pharyngeal arch), which were likely not initially fused to or rigidly connected with the skull.
(2) In osteichthyans, jaws became more securely attached to the skull *via* amphistylic jaw suspensions, potentially largely limiting chewing to the lower jaw movements, at least initially. However, the cranium became highly kinetic, allowing for intracranial and/or suspensory motions. While extant early‐branching actinopterygians apparently predominantly employ more arcuate forms of chewing, cranio‐suspensory chewing cannot be dismissed as an ancestral condition.
(3) Teleosts, the most numerically diverse group of vertebrates, also display the functionally most varied array of oropharyngeal food‐processing adaptations. They have evolved unique processing solutions across all three known processing centres of the oropharyngeal cavity, including mandibular, hyoid, and branchial arch derivatives.
(4) Early sarcopterygians possessed a distinctive intracranial joint, a feature retained in coelacanths and fossil dipnotetrapodomorpha (rhipidistians) that likely facilitated cranio‐suspensory chewing. Subsequently, the intracranial joint was lost in both crown‐group lungfish and stem tetrapods, resulting in the loss of their capacity for intracranial motion. Furthermore, both crown lungfish and tetrapods evolved autostylic jaw suspensions, with jaws becoming directly anchored to the cranium, which likely initially constrained their feeding behaviour to simple arcuate chewing; regardless, pronounced intracranial mobility reappeared independently in certain squamates and birds.
(5) We have demonstrated here that both mastication and pseudomastication, which are present in lungfishes, salamanders, turtles, the tuatara, and mammals, functionally resemble dimensionally complex mandible‐based chewing *via* the jaw joint (i.e. functionally masticatory behaviours). This finding challenges the assumption that dimensionally complex (functionally masticatory) behaviours are unique to mammals and demonstrates that they evolved independently multiple times beyond the emergence of the TMJ. The occurrence of these behaviours in the tuatara, turtles, and mammals further raises the question of whether similar processing strategies were present in early amniotes.

## AUTHOR CONTRIBUTIONS

Conceptualization: D. S.; Data curation: D. S.; Formal Analysis: D. S.; Funding acquisition: D. S., N. K.; Investigation: D. S., M. M., S. H., A. H., P. L., L. D. C., N. K.; Methodology: D. S., S. H.; Project administration: D. S.; Resources: D. S., S. H., A. H., P. L., N. K.; Supervision: D. S.; Validation: D. S., M. M., S. H., A. H., P. L., L. D. C., N. K.; Visualization: D. S.; Writing – original draft: D. S.; Writing – review & editing: D. S., M. M., S. H., A. H., P. L., L. D. C., N. K.

## DECLARATION ON THE USE OF AI

Certain sections of this manuscript were revised for grammar and style using AI‐based tools, including Grammarly (San Francisco, CA, USA) as well as via a locally hosted version (LibreChat) of ChatGPT (OpenAI, San Francisco, CA, USA). All recommendations generated by these tools were thoroughly assessed for accuracy and appropriateness before being incorporated. The authors accept sole responsibility for the manuscript's intellectual content and interpretations.

## Supporting information


**Table S1.** Functional morphology of oropharyngeal food processing in selected vertebrates.
**Appendix S1.** Rules and considerations for novel systematic terminology.
**Fig. S1.** Overview of three‐dimensional (3D) movements along the axes *X*, *Y* and *Z* and how they relate to the six degrees of freedom and the anatomical terms of location in relation to cranial movements in quadrupeds and most fishes.
**Table S2.** Terminology of three dimensional (3D) mandibular movements.

**Video S1.** *Cuora mouhotii* feeding.

**Video S2.** *Testudo hermanni* feeding.

**Video S3.** *Geochelone carbonaria* feeding.

**Video S4.** *Manouria emys* feeding.

**Video S5.** *Sternotherus odoratus* feeding.

## Data Availability

The data that supports the findings of this study are available in the supplementary material of this article.
