# Supplementary material for: Beyond mammals: the evolution of chewing and other forms of oropharyngeal food processing in vertebrates
Source: Biol Rev Camb Philos Soc. 2026 Jan 20;101(3):1406–62. doi: 10.1002/brv.70129 (PMC13149792; doi:10.1002/brv.70129)
Supplement: Supplementary file 1 — Table S1. Functional morphology of oropharyngeal food processing in selected vertebrates. Appendix S1. Rules and considerations for novel systematic terminology. Fig. S1. Overview of three‐dimensional (3D) movements along the axes X, Y and Z and how they relate to the six degrees of freedom and the anatomical terms of location in relation to cranial movements in quadrupeds and most fishes. Table S2. Terminology of three dimensional (3D) mandibular movements. [file BRV-101-1406-s001.docx]

**Table S1.** Functional morphology of oropharyngeal food processing in selected vertebrates.

| **Systematics** | | | | **Functional morphological traits** | | **Medium** | **Processing behaviour** | **References** |
| --- | --- | --- | --- | --- | --- | --- | --- | --- |
| **Cyclostomata** | **Myxinoidea** | **Myxiniformes**  (hagfish) | ***Myxine glutinosa*** | *non-gnathostome: no mandibular jaws* | Water | | PMP | https://youtu.be/_8FVpj0p-iU |
|  | **Petromyzontida** | **Petromyzontiformes** (lampreys) | ***Ichthyomyzon unicuspis*** | *non-gnathostome: no mandibular jaws* | Water | | PMP | https://youtu.be/AzZao6SVMyc https://youtu.be/3UCe2YqO45k |
| **Chondrichthyes** | **Holocephali** | **Chimaeridae** (short-nosed chimaeras) | ***Hydrolagus colliei*** | *autostylic jaw suspension. hinge-like jaw joint* | Water | | AC | https://youtu.be/ZMxDrzbk92U  https://youtu.be/BntGkQs90BQ  (MorphoSource: UF:Fish: #unknown, ms-id: 000582573) |
| **Teleostei** | **Osteoglossomorpha** | Hiodontiformes | ***Hiodon tergisus*** | *hyobranchial processing centre, no protrusible jaws* | Water | | R | (MorphoSource: UF:Fish:137015, ms-id: 000051250)  (Sanford & Lauder, 1989; Hilton, 2001) |
|  |  | Osteoglossiformes | ***Arapaima gigas*** | *hyobranchial processing centre, no protrusible jaws* | Water | | R | (MorphoSource: UF:Fish:33107, ms-id: 000051346)  (Sanford & Lauder, 1989; Hilton, 2001) |
|  |  |  | ***Heterotis niloticus*** | *hyobranchial processing centre, no protrusible jaws* | Water | | R | (MorphoSource: UF:Fish:32897, ms-id: 000051345)  (Sanford & Lauder, 1989; Hilton, 2001) |
|  |  |  | ***Osteoglossum bicirrhosum*** | *hyobranchial processing centre, no protrusible jaws* | Water | | R | (MorphoSource: UF:Fish:189007, ms-id: 000051293)  (Sanford & Lauder, 1989; Hilton, 2001) |
|  | **Elopomorpha** | Elopiformes | ***Elops saurus*** | *protrusible maxillary, no hyobranchial processing centre, hyobranchial filter apparatus* | Water | | NOPE | (MorphoSource: TCWC:Ichthyology:462.02, ms-id: 000056027)  (Vrba, 1968) |
|  |  | Albuliformes | ***Albula vulpes*** | *hyobranchial processing centre, no protrusible jaws* | Water | | R? | (MorphoSource: UF:Fish:207902, ms-id: 000051226)  (Hilton, 2001) |
|  |  |  | ***Megalops atlanticus*** | *protrusible maxillary, no hyobranchial processing centre, hyobranchial filter apparatus* | Water | | NOPE | (MorphoSource: UF:Fish:78923, ms-id: 000043555)  (Jud *et al*., 2011) |
|  | **Otocephala** | Clupeiformes | ***Clupea harengus*** | *protrusible maxillary, no hyobranchial processing centre, hyobranchial filter apparatus* | Water | | NOPE | (MorphoSource: UF:Fish:184063, ms-id: 000533036)  (Batty *et al*., 1986; Dalpadado, 2000) |
|  |  | Alepocephaliformes | ***Platytroctes apus*** | *protrusible maxillary, no hyobranchial processing centre, hyobranchial filter apparatus* | Water | | NOPE | (MorphoSource: UF:Fish:166458, ms-id: 000051412)  (Novotny, 2018) |
|  |  | Gonorynchiformes | ***Chanos chanos*** | *protrusible maxillary, no hyobranchial processing centre* | Water | | NOPE | (MorphoSource: UWFC:UW 040804, ms-id: 000070861)  (Taki *et al*., 1987) |
| **Lissamphibia** | **Apoda**  **(caecilians)** | **Ichthyophiidae** (Asiatic tailed caecilians) | ***Ichthyophis* sp.** | *(gliding?) hinge-like jaw joint, suspension not osseously fused, large retroarticular process* | Air | | B or AC? | (MorphoSource: UF:Herp:76734, ms-id: 000049706)  https://youtu.be/7FljvSPOxxI |
|  |  | **Typhlonectidae** (aquatic caecilians) | ***Typhlonectes natans*** | *(gliding?) hinge-like jaw joint, suspension not osseously fused, huge retroarticular process* | Water | | NOPE | (MorphoSource: UF:Herp:192436, ms-id: 000649718)  https://youtu.be/7KenL5H9KK4 |
|  | **Anura**  **(frogs)** | **Pipidae** (tongueless frogs) | ***Xenopus laevis*** | *saddle-like jaw joint, edentulous, unfused interramal joint, clawed hands and feet* | Water | | NOPE | (MorphoSource: CAS:SUA:2234, ms-id: 000167873)  https://youtu.be/wMVWekk-KFw https://youtu.be/K4Axmpcc5_U |
|  |  |  | ***Hymenochirus boettgeri*** | *saddle-like jaw joint, edentulous, unfused interramal joint, clawed hands and feet* | Water | | NOPE | (MorphoSource: UF:Herp:27563, ms-id: 000101128)  https://youtu.be/6vJZerkzsv4 |
| **Sauropsida** | **Rhynchocephalia**  **(“beak-heads”)** | **Sphenodontidae** ("wedge-teethed") | ***Sphenodon punctatus*** | *gliding saddle jaw joint* | Air | | PM | (MorphoSource: UF:Herp:11978, ms-id: 000011009)  https://youtu.be/1TUEb4zawOo https://youtu.be/ei9r7B9HzfY https://youtu.be/JOE5t4tDcKk |
|  | **Testudines**  **(turtles)** | **Chelidae**  (Austro-South American side-necked turtles) | ***Chelus fimbriata*** | *hinge-like jaw joint* | Water | | AC | (MorphoSource: UF:Herp:117469, ms-id: 000574622)  https://youtu.be/4OJjbqaGo_k&t=64 |
|  |  | **Pelomedusidae**  (African side-necked turtles) | ***Pelomedusa subrufa*** | *hinge-like jaw joint* | Water | | AC | (MorphoSource: UF:Herp:85211, ms-id: 000606215)  https://youtu.be/Q7EeBGsEWSM&t=307 |
|  |  | **Podocnemididae** | ***Podocnemis unifilis*** | *ball plane jaw joint* | Water | | PM | (MorphoSource: UF:Herp:191763, ms-id: 000605789)  https://youtu.be/0vH5w1kyfr4?si |
|  |  | **Carettochelyidae** | ***Carettochelys insculpta*** | *gliding saddle jaw joint* | Water | | PM | (MorphoSource: UF:Field#:49415, ms-id: 000615515)  https://youtu.be/YDgvovFuWMI |
|  |  | **Trionychidae**  (softshell turtles) | ***Pelodiscus sinensis*** | *hinge-like jaw joint* | Water | | AC | (MorphoSource: UF:Herp:52509, ms-id: 000423509)  https://youtu.be/PGhn2kTAVDw |
|  |  | **Emydidae**  (terrapins or marsh turtles) | ***Graptemys barbouri*** | *gliding saddle jaw joint* | Water | | PM | (MorphoSource: UF:Herp:61899, ms-id: 000046483)  https://youtu.be/wtJ-bTXwxY4 |
|  |  | **Platysternidae**  (big-headed turtle) | ***Platysternon megacephalum*** | *hinge-like jaw joint* | Water | | AC | (MorphoSource: UF:herp:191750, ms-id: 000574697)  https://youtu.be/jT1IHQ4_u9E&t=414 |
|  |  | **Geoemydidae**  (leaf turtles) | ***Cuora mouhotii*** | *gliding saddle jaw joint* | Air | | PM | µCT scan from (Blüml, 2019)  **Video S1** |
|  |  | **Testudinidae**  (tortoises) | ***Testudo hermanni*** | *gliding saddle jaw joint* | Air | | PM | (MVZ:Herp:238087, ms-id: 000385515)  **Video S2** |
|  |  |  | ***Geochelone* sp.** | *gliding saddle jaw joint* | Air | | PM | (MVZ:Herp:233519, ms-id: 000423820)  **Video S3** |
|  |  |  | ***Manouria emys*** | *gliding saddle jaw joint* | Air | | PM | (MorphoSource: UMMZ:herps:227759, ms-id: 000070107)  **Video S4** |
|  |  | **Cheloniidae**  (bony marine turtles) | ***Chelonia mydas*** | *gliding saddle jaw joint* | Water | | PM | (MorphoSource: UF:Herp:51413, ms-id: 000413162)  https://youtu.be/KP9XE5zw5qw |
|  |  | **Dermochelyidae**  (leathery marine turtles) | ***Dermochelys coriacea*** | *hinge-like jaw joint* | Water | | AC | (MorphoSource: UF:Herp:84769, ms-id: 000574728)  https://youtu.be/TCQniQ1Lpoc&t=185 |
|  |  | **Chelydridae**  (snapping turtles) | ***Chelydra serpentina*** | *hinge-like jaw joint* | Water | | AC | (MorphoSource: OUVC:10681, ms-id: 000076039)  https://youtu.be/LPZ1ieLg1X4 |
|  |  | **Dermatemydidae** | ***Dermatemys mawii*** | *gliding saddle jaw joint* | Water | | PM | (MorphoSource: UF:Herp:84769, ms-id: 000574728)  https://youtu.be/JARIsOzePIY |
|  |  | **Kinosternidae** | ***Claudius angustatus*** | *gliding saddle jaw joint* | Water | | PM | https://youtu.be/Z1V9IYxhunI&t=2 |
|  |  |  | ***Sternotherus minor*** | *gliding saddle jaw joint* | Water | | PM | https://youtu.be/nY1SRTpC1g4 |
|  |  |  | ***Sternotherus odoratus*** | *gliding saddle jaw joint* | Water | | PM | (MorphoSource: YPM:VZ:YPM HERR 019736.001, ms-id: 000072565)  **Video S5** |
|  | **Crocodilia**  **(crocodilians)** | **Alligatoridae**  (alligators and caimans) | ***Alligator mississippiensis*** | *hinge-like jaw joint* | Air | | AC | (MorphoSource: ouvc:11415, ms-id: 000072948)  https://youtu.be/92rHy4tmcVY |
|  |  | **Crocodylidae**  (true crocodiles) | ***Mecistops cataphractus*** | *hinge-like jaw joint* | Air | | AC | (MorphoSource: tmm:m:3529, ms-id: 000114916)  https://youtu.be/-MajaDNmwzM https://youtu.be/kzQRNJV_4Ew |
|  |  |  | ***Osteolaemus tetraspis*** | *hinge-like jaw joint* | Air | | AC?, B, IB | (MorphoSource: UF:Herp:33749, ms-id: 000395006)  https://youtu.be/6SpL_3sXRbE |
|  |  | **Gavialidae**  (‘gharials’) | ***Gavialis gangeticus*** | *hinge-like jaw joint* | Air | | AC?, B, IB | (MorphoSource: UF:Herp:33421, ms-id: 000491536)  https://youtu.be/HUdEIBMNVwk |
|  |  |  | ***Tomistoma schlegelii*** | *hinge-like jaw joint* | Air | | AC?, B, IB | (MorphoSource: UF:Herp:33422, ms-id: 000456286)  https://youtu.be/YvVv9yv3Q4g |

Modes of oropharyngeal food processing: AC, arcuate chewing; B, bites; IB, ingestive bites; PM, pseudomastication; NOPE, no oropharyngeal processing evident; PMP, pseudomandibular processing; R, raking; ? = uncertainty regarding the respective processing behaviour. Note that, to keep this table concise, we have omitted species or taxa whose feeding behaviours are already comprehensively described in the literature and thoroughly cited in our review. Additionally, where chewing has been identified, it is assumed that both bites and ingestive bites are included in the animal’s behavioural repertoire; as a result, these actions are not individually recorded.

**Appendix S1.** **Rules and considerations for novel systematic terminology**

(1) This novel terminology aims to avoid references to developmental embryonic precursor structures, understanding that the complex and interconnected ontogenetic development of the head’s skeletal elements could hinder its broad applicability. Instead, the framework focuses on general, morpho-functional traits.

(2) Wherever plausible, we use existing and well-established concepts and terms. In cases where these have evolved over time and have been used in more narrow or slightly different ways than initially described, we try to follow the well-established usage of concepts instead of reverting to their original description if possible. For example, the usage of mesokinesis is thus restricted to movements in the dermal skull roof that occur behind the eye and not for all movements within the skull roof, as initially described (Versluys, 1910, 1912)*.*

(3) When established terms and concepts are unintelligible, exclusionary, and/or not intuitive, thus preventing the broad adoption of the new framework, we provide simple, novel alternatives or try to reframe established terms.

(4) When multiple previously described terms characterise the same general morpho-functional trait, the best-established term is used.

(5) Where no, or no adequate vocabulary existed, we propose novel terminology.

(6) Terminology is purely of functional/morphological character and, hence, has no phylogenetic value and does not imply inheritance from a common ancestor. For example, ‘pseudomastication’ is used to refer to the dimensionally complex chewing of various non-mammalian vertebrates and ‘streptostyly’ is used for both squamates and birds, although these features evolved independently in these groups.

**
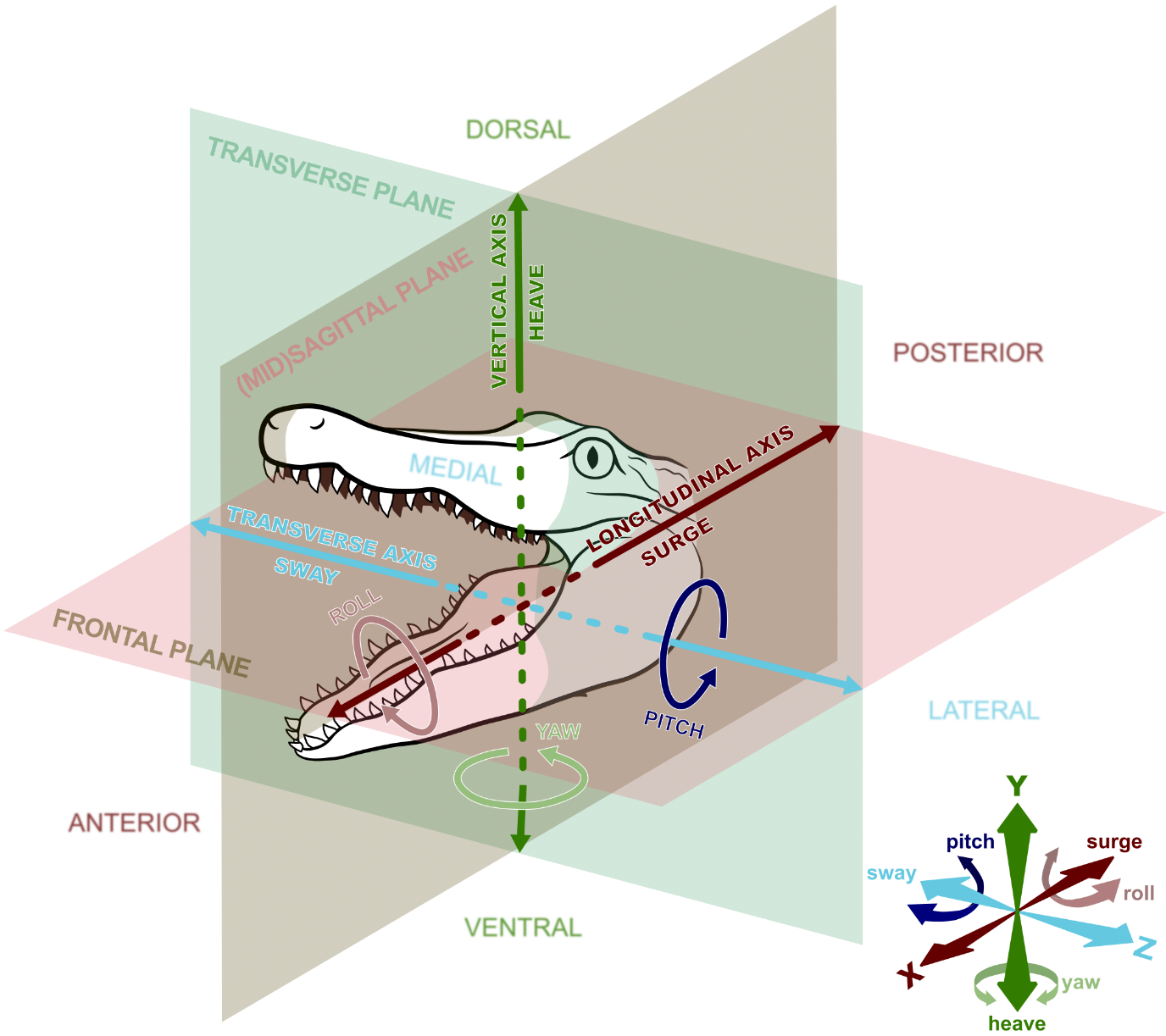
Fig. S1.** Overview of three-dimensional (3D) movements along the axes *X*, *Y*, and *Z* and how they relate to the six degrees of freedom and the anatomical terms of location in relation to cranial movements in quadrupeds and most fishes. Note that the axes and planes have various names, which may be familiar to varying degrees across different scientific disciplines. Some of these names for planes correspond to the names of certain axes. However, since each plane contains two axes, it would be incorrect to associate a plane with just one axis. To ensure clarity and simplicity, we have carefully chosen distinct terms for each. *Axes (alternative terminology and brief description)*: along *X* = *longitudinal axis* (anteroposterior, sagittal, fore-aft axis), the axis that runs from the front (anterior) to the back (posterior) of the body, rotational movements around this axis result in roll; along *Y* = *transverse axis* (horizontal, lateral, left–right axis), this axis runs from left to right across the body and movements around this axis result in pitch; along Z = *vertical axis* (dorsoventral, superior–inferior, up–down axis), this axis runs vertically from top (superior or dorsal) to bottom (inferior or ventral) and movements around this axis result in yaw. *Planes (alternative terminology and brief description)*: connecting *Y* and *Z* = *transaxial plane* (horizontal, axial, or transverse plane), this plane divides the body into anterior and posterior sections (or dorsal and ventral in humans) and points on this plane have a constant *X*-coordinate; connecting *X* and *Y* = *coronal plane* (frontal or vertical plane), the plane that divides the body into dorsal and ventral sections (or anterior and posterior in humans) and points on this plane have a constant *Z*-coordinate; connecting *X* and *Z* = *median plane* (lateral, [mid]sagittal, longitudinal plane) which divides the body into (equal) right and left sections and points on this plane have a constant *Y*-coordinate. *Brief description of the six degrees of freedom*: *surge* (longitudinal or fore–aft movements [pro- and retraction or retrusion]); *roll* ([long-axis] rotation around the longitudinal axis); *sway* (lateral or side-to-side translation); *pitch* (vertical rotational opening-closing jaw movements around the transverse axis); *heave* (vertical or up–down translation); *yaw* (left and right rotation around the vertical axis of the jaw or hemimandibles).

**Table S2.** Terminology of three-dimensional (3D) mandibular movements. This overview examines the full range of possible three-dimensional mandibular movements, encompassing six degrees of freedom (DOF) (see Fig. S1). Unlike studies focused on occlusion (as in dentistry), this analysis considers movements of the entire mandible or hemimandible. Traditional occlusion-focused studies often conflate rotations and translations due to their interconnected nature. For instance, vertical rotational movements of the mandible, or pitch, are typically associated with the elevation and depression of the teeth, which corresponds to heave. However, in the context of whole mandibular movements, pitch (rotation) is distinct from heave (translation). Note that wishboning – the medio-lateral or side-to-side movement of the hemimandibles relative to each other – results from a combination of interramal yaw and gliding motions (sway and surge) along the jaw joint. Alternatively, pronounced wishboning may necessitate disarticulation of the jaw joint’s articular surfaces. Understanding these distinctions is crucial for accurately describing the complex biomechanics of mandibular movements. The specialised, bold terms for jaw movements are preferred over the more general DOF as they clearly convey the relationship between anatomical directions, axes, and types of movements involved. The newly introduced terms for vertical- and transverse-axis motions are intended to create greater consistency and simplify the understanding of mandibular motions. Employing this terminology allows for a straightforward reference to all six degrees of mandibular motion as longitudinal, transverse, and vertical translations and rotations. This approach may be argued to be more universally applicable and easier to comprehend.

| **Degree of freedom** | **Description** | **Respective jaw movement terminology** | **Description** |
| --- | --- | --- | --- |
| Surge | Translation along the longitudinal axis | **Long(itudinal)-axis translation**: **longitudinal motion**, propalinal, proal, antero-posterior, pro- and retraction, pro- and retrusion, or fore-and-aft | Anterior and posterior gliding resulting in linear forth and back movements of the jaw or individual hemimandibles |
| Sway | Translation along the transverse axis | **Trans(verse)-axis translation**: **transverse motion**, medio-lateral, side-to-side, or left and right | Medial and lateral gliding resulting in linear side-to-side movements of the jaw or individual hemimandibles |
| Heave | Translation along the vertical axis | **Vert(ical)-axis translation**: **vertical motion**, elevation and depression, dorso-ventral, or superio-inferior | Dorsal and ventral gliding or displacement resulting in linear opening–closing movements of the jaw or individual hemimandibles |
| Roll | Rotation around the longitudinal axis | **Long(itudinal)-axis rotation** (‘mandibular roll’): eversion and inversion | Eversion and inversion rotation resulting in arcuate medio-lateral excursion movements of the jaw or individual hemimandibles |
| Pitch | Rotation around the transverse axis | **Trans(verse)-axis rotation**: arcuate, arcilineal, arcilinear, orthal, shearing | Dorsal and ventral rotation resulting in arcuate opening–closing movements of the jaw or individual hemimandibles |
| Yaw | Rotation around the vertical axis | **Vert(ical)-axis rotation**: left-and-right, medio-lateral, or bucco-lingual | Buccal and lingual rotation resulting in arcuate medio-lateral movements of the jaw or individual hemimandibles |

**Video S1.** *Cuora mouhotii* feeding. Note the retraction of the lower jaw during the power stroke phase of biting and its subsequent protraction to its anterior position during jaw opening. Video recorded at the Department of Comparative Anatomy and Morphology, Institute of Zoology, University of Vienna, Vienna, Austria.

**Video S2.** *Testudo hermanni* feeding. Note the pronounced retraction of the lower jaw during the power stroke phase of biting and its subsequent protraction to its anterior position during jaw opening. Video recorded at the Department of Comparative Anatomy and Morphology, Institute of Zoology, University of Vienna, Vienna, Austria.

**Video S3.** *Geochelone carbonaria* feeding. Note the retraction of the lower jaw during the power stroke phase of biting and its subsequent protraction to its anterior position during jaw opening. Video recorded at the Department of Comparative Anatomy and Morphology, Institute of Zoology, University of Vienna, Vienna, Austria.

**Video S4.** *Manouria emys* feeding. Note the pronounced retraction of the lower jaw during the power stroke phase of biting and its subsequent protraction to its anterior position during jaw opening. Video recorded at the Department of Comparative Anatomy and Morphology, Institute of Zoology, University of Vienna, Vienna, Austria.

**Video S5.** *Sternotherus odoratus* feeding. Note the slight retraction of the lower jaw during the power stroke phase of biting and its subsequent protraction to its anterior position during jaw opening. Video recorded at the Department of Comparative Anatomy and Morphology, Institute of Zoology, University of Vienna, Vienna, Austria.
